# Antibacterial and Multi-Target Immunomodulatory Mechanisms of Marine *Nocardia rhizosphereae* Metabolites: Experimental and Network Pharmacology

**DOI:** 10.3390/microorganisms14102179

**Published:** 2026-09-28

**Authors:** Tsolanku Sidney Maliehe, Wanda Shekwa, Ofentse Jacob Pooe, Peter Masoko

**Affiliations:** 1 Department of Biochemistry, Genetic and Microbiology, University of KwaZulu Natal, Private Bag X54001, Durban 4000, South Africa; pooeo@ukzn.ac.za; 2 Department of Biochemistry, Microbiology and Biotechnology, University of Limpopo, Private Bag X1106, Polokwane 0727, South Africa; wandashekwa@gmail.com (W.S.); peter.masoko@ul.ac.za (P.M.)

**Keywords:** *Nocardia rhizosphereae*, antibacterial activity, antibacterial mechanism, network pharmacology, molecular docking

## Abstract

The constant increase in multidrug resistance has necessitated bioprospecting of marine actinomycetes for potent antibacterial and immunomodulatory activities. The aim of this study was to investigate the antibacterial and potential host-immunomodulatory mechanisms of secondary metabolites from marine *Nocardia rhizosphereae*. The extract of *N. rhizosphereae* was characterised using Gas Chromatography–Mass Spectrometry (GC-MS), and its minimum inhibitory concentration (MIC) was determined by a microdilution assay. Network pharmacology analysis, Gene Ontology (GO) and Kyoto Encyclopaedia of Genes and Genomes (KEGG) pathway enrichment analyses were performed using ShinyGO to elucidate potential host-immunomodulatory mechanisms against bacterial infections. Molecular docking was done using Attracting Cavities on SwissDock. GC–MS spectra revealed 33 compounds, of which 11, including 2-pentanone, 4-amino-4-methyl (50.11%) and 2-hexanol, 2-methyl (25.4%), were reported in extracts with antibacterial activities. The extract exhibited MIC values ranging from 19.55 to 312.5 µg/mL. Key hub targets included ESR1, HSP90AA1 and PPARG. GO enrichment revealed 457 molecular functions, while KEGG analysis illustrated 156 pathways. The best docking poses ranged between –4.99 and −8.69 kcal/mol. In conclusion, the metabolites showed potential antibacterial activity through disruption of bacterial cell-envelope integrity and potential modulation of multiple host-associated molecular targets and signalling pathways.

## 1. Introduction

Bacterial infections remain a major global public health challenge. Although most of these can be treated symptomatically or cured by antimicrobial therapy, the rapid emergence of antimicrobial resistance (AMR) among pathogens, with the recent occurrence of panantibiotic-resistant infections, reduces the effectiveness of existing standard antibiotics [1,2]. This is driven by factors such as the misuse and overuse of antibiotics both in human and veterinary medicine [3,4]. Consequently, globally, bacterial AMR is directly responsible for more than 1.2 million deaths and is associated with over 4.95 million deaths annually [5]. Furthermore, the emergence of MDR pathogens not only exacerbates the complexity of infections but also extensively contributes to increased healthcare costs and poorer clinical treatment outcomes [6]. Therefore, the alarming rise in the AMR crisis highlights the urgent need for novel antimicrobial agents with distinct multi-target mechanisms.

Pathogenic organisms can evade or suppress normal host immune mechanisms through the expression of specific virulence factors, thereby contributing substantially to pathogenicity and disease progression. Consequently, the outcome of a bacterial infection is determined not only by the ability of an antibacterial compound to eliminate or suppress the pathogen but also by the capacity of the host to mount an effective and appropriately regulated immune response [7]. Although conventional antibacterial agents that directly target pathogens remain essential for the management of bacterial infections, complementary therapeutic approaches are increasingly needed, particularly in the context of antimicrobial resistance (AMR). One such approach is host-directed therapy (HDT), which aims to enhance protective immune responses by interfering with host mechanisms exploited by pathogens for replication or persistence, stimulating host defence mechanisms, and modulating host pathways that are dysregulated during infection [8]. Unlike conventional anti-infective therapies that primarily exert selective pressure on pathogens, HDT targets host processes and may therefore be less susceptible to the development of pathogen-derived therapeutic resistance. Accordingly, compounds that combine direct antibacterial activity with beneficial modulation of host immune responses may provide an important therapeutic advantage. Such agents could potentially suppress bacterial proliferation while simultaneously enhancing or appropriately regulating host defence pathways, thereby contributing to more effective control of bacterial infections.

Actinobacteria are high G + C content Gram-positive bacteria, grouped under the Actinomycetota phylum, a clade intermediary between bacteria and fungi. They are well known for producing metabolites that directly inhibit bacterial growth through mechanisms involving disruption of cell-wall synthesis, inhibition of protein and nucleic acid synthesis, interference with essential metabolic pathways, and damage to bacterial membranes [9]. Thus, terrestrial actinomycetes have historically been widely bioprospected for bioactive secondary metabolites, which account for approximately two-thirds of clinically relevant antibiotics. Historic breakthroughs include the discovery of erythromycin, neomycin, kanamycin, and lincomycin, which marked a milestone in the fight against infectious diseases [10,11]. However, over the past two decades, the number of novel antibiotics discovered has been declining. The decline is linked to genetic modifications among species during evolution, resulting in the production of structurally similar compounds to those previously characterised [12,13]. This has, thus, shifted research to bioprospecting promising rare actinomycetes from less-tapped environments such as marine environments.

Marine environments represent an especially promising source of actinomycete-derived bioactive metabolites. Marine actinomycetes inhabit unique environments characterised by high salinity and nutrient limitation, which promote diverse metabolic pathways and the expression of specialised biosynthetic gene clusters (BGCs) [14]. These adaptations enable the production of structurally diverse secondary metabolites with potent biological activities, including antibacterial effects [15]. Despite this, relatively little work on potent secondary metabolites from marine actinomycetes has been reported owing to their low culturability [16]. For example, Chen et al. [17] and Huang et al. [18] have reported a new tricyclic polyene macrolactam, Weddellamycin, from marine *Streptomyces* sp. DSS69, Eravacycline (Xerava^®^), a fluorocycline which is a semisynthetic derivative of tetracycline from *Streptomyces* and Glycoabyssomicin A, a novel abyssomicin variant from *Streptomyces koyangensis* SCSIO5802, which exhibit potent broad-spectrum antimicrobial activities, underscoring their pharmaceutical potential.

*Nocardia rhizosphereae*, a filamentous Actinobacterium of the order Corynebacteriales, is one of the underexplored actinobacterial species within the genus *Nocardia* [19]. Although, like other members of the genus *Nocardia*, *N. rhizosphereae* might possess considerable biosynthetic potential for the production of bioactive metabolites, the antibacterial mechanisms of its metabolites remain insufficiently characterised, particularly regarding their ability to simultaneously affect bacterial pathogens and host immune responses. In this study, *N. rhizosphereae* was previously isolated from marine sediments and screened for the production of antibacterial compounds.

The interaction between antibacterial compounds and the host immune system is complex and may involve multiple molecular targets and interconnected signalling pathways. Network pharmacology, an integrative approach combining systems biology and bioinformatics, enables effective evaluation of “multi—target and multi—pathway effects” of bioactive compounds, intuitively elucidating their mechanisms of action [20]. By integrating antibacterial compounds with their predicted host targets, we can elucidate mechanisms by which antibacterial compounds influence host biological processes and disease-associated pathways during infection. This approach is particularly relevant as it enables the elucidation of mechanisms by which a single or a combination of metabolites may interact with multiple targets, modulating different biological processes and disease-associated pathways during infection [21,22,23].

This study aimed to elucidate the potential dual antibacterial and host-immunomodulatory mechanisms of selected *Nocardia rhizosphereae*-derived metabolites through integrated experimental and network pharmacology. The antibacterial mechanism of *N. rhizosphereae*-derived secondary metabolites was evaluated by assessing their effects on the release of intracellular cellular constituents by the test bacteria. Network pharmacology was subsequently employed to investigate the host-immunomodulatory mechanisms and signalling pathways potentially influenced by the selected metabolites.

## 2. Materials and Methods

### 2.1. Resuscitation

The representative strain, *N. rhizosphereae*, was previously isolated from the marine sediment sample collected from Isphingo Beach (29.8578° S, 31.0342° E) in Durban, KwaZulu-Natal Province, South Africa, using Actinomycetes Isolation Agar. It was resuscitated on Luria–Bertani (LB) medium (10 g/L tryptone, 5 g/L yeast extract, 10 g/L sodium chloride) at 37 °C, 150 rpm for 24 h. Both media were obtained from Sigma (Merck KGaA, Darmstadt, Germany).

### 2.2. Solvent-Based Extraction of Secondary Metabolites

The selected isolate was cultured in production medium to obtain secondary metabolites. A standardised seed culture (1 × 10^8^ CFU/mL) was used to inoculate 250 mL of production medium, followed by incubation at 30 °C and 150 rpm for 7 days. The culture broth was centrifuged (5000 rpm for 30 min) to obtain a cell-free supernatant, from which secondary metabolites were extracted using ethyl acetate (1:1, *v*/*v*). The extraction solvent was selected based on its polarity and its immiscibility with water. Thereafter, the solvent was evaporated, and the crude extract was reconstituted in 70% acetone to a final concentration of 10 mg/mL [24].

### 2.3. Chemical Characterisation of Volatile Compounds of the Extract

The chemical composition of the extract was analysed by Gas Chromatography–Mass Spectrometry (GC–MS) using a Trace GC Ultra gas chromatograph coupled to a DSQ mass spectrometer (Thermo Scientific, Waltham, MA, USA) and fitted with an HP-5 MS capillary column (30 m × 0.25 mm × 0.25 μm) (Agilent Technologies, Santa Clara, CA, USA). The oven temperature was held at 40 °C for 3 min, increased at 5 °C/min to 105 °C, then at 6 °C/min to 220 °C, and held for 10 min. The injector temperature was maintained at 250 °C. Helium served as the carrier gas at a flow rate of 1.0 mL/min with a split ratio of 10:1. The mass spectrometer was operated at an ionisation energy of 70 eV and an ion source temperature of 250 °C [25].

### 2.4. Evaluation of Antibacterial Activity of the Extract

#### 2.4.1. Agar Well Diffusion

Antibacterial activity of the extract was evaluated using the agar well diffusion assay. Test bacterial inocula (*Staphylococcus aureus* ATCC 25925, *Pseudomonas aeruginosa* ATCC 27853, *Streptococcus pyogenes* (clinical isolate), methicillin-resistant *Staphylococcus aureus* (MRSA) ATCC 43300, and *Escherichia coli* ATCC 25922) were standardised to 0.5 McFarland (≈1.5 × 10^8^ CFU/mL), swabbed onto the Mueller–Hinton agar plates, and 40 µL of the extract was added to each well. Plates were incubated at 37 °C for 24 h. Antibacterial activity was determined by measuring the zones of inhibition [26].

#### 2.4.2. Minimum Inhibitory Concentration (MIC) of the Extract

The minimum inhibitory concentration (MIC) of the extract was determined using the 96-well broth microdilution method according to Clinical and Laboratory Standards Institute (CLSI) guidelines [27]. Serially diluted extract was incubated with standardised test bacterial suspensions (1 × 10^8^ CFU/mL). Ciprofloxacin (20 µg/mL) and 70% acetone served as the positive and negative controls, respectively. After incubation at 37 °C for 24 h, iodonitrotetrazolium violet (INT) Sigma (Merck KGaA, Darmstadt, Germany) was added, and the MIC was recorded as the lowest extract concentration that inhibited visible bacterial growth [28].

### 2.5. Effect of the Secondary Metabolites on the Cell Release Constituents

Bacterial cell membrane integrity was evaluated by measuring the leakage of intracellular nucleic acids and proteins. Briefly, test bacterial suspensions were treated with secondary metabolites at a concentration of 2× MIC and incubated at 37 °C and 150 rpm for 4 h, with untreated cells serving as the control. After centrifugation (8000 rpm, 10 min), the absorbance of the supernatants was measured at 260 and 280 nm to quantify nucleic acid and protein leakage, respectively [29].

### 2.6. Drug-Likeness Properties of the Compounds

GC–MS–identified compounds were screened for reported antibacterial activity and evaluated for drug-likeness using the SwissADME web tool (https://www.swissadme.ch/index.php accessed on 17 September 2026) [30]. The Simplified Molecular Input Line Entry System (SMILES) strings were retrieved from the PubChem database (https://pubchem.ncbi.nlm.nih.gov/) 12 November 2025 [31] and submitted to SwissADME web tool (https://www.swissadme.ch/) on the 13 November 2025 for prediction of physicochemical properties and drug-likeness [32]. Drug-likeness was assessed according to Lipinski’s rule of five [33], with compounds selected for further analysis based on the following criteria: molecular weight ≤ 500 Da, cLogP ≤ 5, hydrogen bond acceptors ≤ 10, hydrogen bond donors ≤ 5, and rotatable bonds ≤ 10.

### 2.7. Network Pharmacology Study

Potential protein targets of the selected drug-like compounds were predicted using SwissTargetPrediction web tool (http://www.swisstargetprediction.ch/) [34], accessed on 5 January 2026 and the Search Tool for Interactions of Chemicals (STITCH) database v5.0 (https://stitch-db.org/) for chemical-protein interactions. Bacterial infection-related targets were retrieved from GeneCards v6.1 (https://www.genecards.org/) [35] and DisGeNET v26.3 (https://www.disgenet.org/) on 10 January 2026 using the keyword “bacterial infection,” and duplicate entries were removed. Venn analysis (http://bioinformatics.psb.ugent.be/webtools/Venn/), developed by the UGent Venn webtool was accessed on 5 March 2026 to identify overlapping targets between compounds and disease-associated genes. Protein–protein interactions were analysed using the STRING database (v12.0; https://string-db.org/, accessed on 10 March 2026) [36], and the resulting network was visualised in Cytoscape v3.10.4. Hub genes were identified based on degree centrality using the CytoHubba plugin v1.6 [37,38], with highly connected nodes considered key antibacterial targets. Gene Ontology (GO) and Kyoto Encyclopaedia of Genes and Genomes (KEGG) pathway enrichment analyses were performed using ShinyGO v0.86.0 (https://bioinformatics.sdstate.edu/go/) [39] on 10 May 2026. The co-expression characteristics of the top ten hub genes with other functionally related genes were analysed to assess their potential biological functions using GeneMANIA v3.5.3 (http://pages.genemania.org/) [40], accessed on 12 July 2026.

### 2.8. Molecular Docking Analysis

The three-dimensional (3D) structures of the active antibacterial compounds were retrieved from the PubChem database (https://pubchem.ncbi.nlm.nih.gov/) [31] on the 13 August 2026 and prepared for docking using the SwissDock online tool (https://www.swissdock.ch/) [41]. Protein structures of ESR1 (PDB ID: 1XP1), HSP90AA1 (PDB ID: 5CF0), PPARG (PDB ID: 2P4Y), SIRT1 (PDB ID: 4ZZJ), PTGS2 (PDB ID: 1CX2), RELA (PDB ID: 4KV4), MMP9 (PDB ID: 1L6J), TLR4 (PDB ID: 3FXI), NFE2L2 (PDB ID: 2FLU) and APP (PDB ID: 1MWP) were obtained from the Protein Data Bank (PDB) (http://www.rcsb.org/) [42] on 15 August 2026 and similarly prepared using SwissDock. Molecular docking was then performed using SwissDock to evaluate binding affinities. The resulting interactions were analysed using Biovia Discovery Studio 2025 Client [43].

### 2.9. Statistical Analysis

To ensure accuracy and reliability, all experiments were performed in triplicate, and the results are reported as mean values with corresponding standard deviations. Statistical analysis of the data was conducted using analysis of variance (ANOVA), followed by Tukey’s post hoc test to assess the significance of observed differences; results with a *p*-value ≤ 0.05 were regarded as statistically significant.

## 3. Results and Discussions

### 3.1. Analysis of Secondary Metabolites

GC-MS analysis identified 33 compounds in the bacterial ethyl acetate extract. The major compound was 2-pentanone, 4-amino-4-methyl (50.11%), followed by 2-hexanol, 2-methyl (25.4%), phenol, 4,6-di(1,1-dimethylethyl)-2-methyl (4.13%), and pentadecanoic acid (2.85%). The least abundant compounds were pentanone, 4-hydroxy-4-methyl, and 3-ethyl-3-methylheptane, each at 0.09% (Table 1). Among these, eleven compounds have been reported to exhibit antibacterial activity. However, due to resource limitations, GC-MS was employed, despite its inherent limitation in detecting predominantly non-volatile and thermally labile compounds. Therefore, the chemical diversity of the extract may have been underestimated, as the use of complementary techniques such as liquid chromatography–mass spectrometry could have enabled the detection and characterisation of additional non-volatile and thermally labile metabolites.

### 3.2. Antibacterial Activity of the Extract

The extract demonstrated significant inhibitory ability against the selected pathogens, with zones of inhibition ranging from 14 to 23 mm (Table 2). Among the tested pathogens, *S. pyogenes* was the most susceptible strain, with the extract displaying the lowest MIC value of 19.55 µg/mL. *E. coli* and *P. aeruginosa* were the most resistant bacterial strains, with an MIC value of 78.13 µg/mL (Table 2). Moreover, the extract exhibited stronger inhibitory effects against the Gram-positive bacteria (except for *MRSA*), in comparison to the Gram-negatives in both assays. The reduced susceptibility of Gram-negative bacteria is consistent with previous reports [55,56,57] and was attributed to the presence of an outer membrane rich in lipopolysaccharides, which restricted the penetration of antibacterial compounds [58]. The profound antibacterial activity demonstrated by the extract was attributed to the identified antibacterial secondary metabolites (Table 1). In conclusion, the observed antibacterial activity results in this study highlighted the extract as a promising source of antibacterial agents.

### 3.3. Proteins and Nucleic Acid Cell Release

The cytoplasmic contents are vital elements for microbial growth, and their leakage can lead to cell inhibition and possibly cell death [59]. Figure 1 illustrates the effect of the extract on the integrity of the cell membrane. The OD_280_ and OD_260_ values of the extract-treated bacterial strains ranged from 0.10 to 0.43 and 0.03 to 0.44, respectively. It was noted that the OD_280_ and OD_260_ values of all treated bacterial strains were significantly (*p* < 0.05) higher than those of the negative controls, indicative of a significant release of cytoplasmic contents (proteins and nucleic acids). This further implied that the extract disrupted cell membrane integrity, consequently initiating bacterial inhibition [60].

### 3.4. Drug-Likeness of the Antibacterial Compounds

The drug-likeness of the antibacterial compounds was evaluated by determining their physicochemical properties. All the identified antibacterial compounds, except Actinomycin C2, were found to satisfy most of the drug-likeness parameters proposed by Lipinski’s Rule of Five (molecular weight ≤ 500 Da, cLogP ≤ 5, hydrogen bond acceptors ≤ 10, hydrogen bond donors ≤ 5, and rotatable bonds ≤ 10) (Table 3), implying that these compounds have a high probability of being orally bioavailable and may serve as promising candidates for antibacterial drug discovery and development [46,61]. Only the 10 compounds that did not violate Lipinski’s Rule of Five were selected for subsequent analyses, including network pharmacology and molecular docking.

### 3.5. Network Pharmacology

Natural extracts may contain compounds that modulate the host’s immune system to aid in defence against bacterial infections, hence the choice of host targets. The compound targets were found to be 227 in total, whereas the genes implicated in bacterial infection were 1391 (Tables S1 and S2 and Figure 2). About 60 targets were found to be common between the predicted targets of the bioactive compounds and bacterial infection-associated genes (Figure 2A). The PPI network consisted of 60 target nodes and 323 edges (Figure 2B). It revealed that these targets are highly interconnected, indicating coordinated regulation of biological processes involved in host responses to bacterial infection [62]. The compound–target network (Figure 2C) further demonstrated that the identified metabolites exhibit multi-target pharmacological characteristics, with individual compounds interacting with multiple proteins. Hub gene analysis highlighted oestrogen receptor 1 (ESR1), Heat Shock Protein 90 Alpha Family Class A Member 1 (HSP90AA1), peroxisome proliferator-activated receptor gamma (PPARG), Sirtuin 1 (SIRT1), prostaglandin endoperoxide synthase 2 (PTGS2), RELA Proto-Oncogene, NF-κB Subunit (RELA), Matrix Metallopeptidase 9 (MMP9), Toll-like receptor 4 (TLR4), Nuclear Factor, Erythroid 2-Like 2 (NFE2L2) and Amyloid Precursor Protein (APP) as key regulatory proteins (Figure 2D). Each node is scaled according to its degree of connectivity, with nodes displaying higher degree values (represented by the colour scale) appearing larger, indicating their greater significance in the network. ESR1 and AR, a transcriptional regulator, are recognised as modulators of innate and adaptive immune responses and host–pathogen interactions [63,64]. HSP90AA1 stabilises numerous client proteins, including EGFR and ERBB2, involved in immune responses [65]. Moreover, an increase in HSP90AA1 can result in a reduction in bacterial adhesion and invasion, especially in human intestinal cells [62]. PPARG regulates macrophage activation and has been implicated in the modulation of anti-inflammatory responses and mitigation of oxidative stress due to bacterial infections [65]. SIRT1 is a critical host defence enzyme that orchestrates immune responses, cellular metabolism, and autophagy during bacterial infections. It suppresses excessive inflammation through modulation of NF-κB signalling [66]. PTGS2 (COX–2) mediates inflammation induced by infection [67]. RELA (nuclear factor kappa-B (NF-κB) p65) regulates transcription of inflammatory cytokines during bacterial infection [68]. MMP9 is a matrix metalloproteinase that contributes to tissue remodelling and leukocyte migration. It plays a critical role in hosts’ response to infections caused by bacterial pathogens [69,70]. TLR4 is primarily activated by bacterial lipopolysaccharide (LPS), consequently initiating activation of the NF-κB signalling pathway and innate immune responses [71,72]. NFE2L2 controls antioxidant defence mechanisms that protect host tissues from oxidative damage during infection [73]. Based on these insights, it was concluded that the compounds have the potential to interact with diverse targets, consequently mediating simultaneous modulation of different pathways, including inflammatory, immune, and oxidative stress pathways, rather than a single molecular target.

### 3.6. GO Biological Processes

The GO biological process enrichment analysis revealed 1000 processes. It revealed that the intersecting target genes were predominantly involved in cellular response to chemical stimulus and response to chemicals (Figure 3A, Table S1). During bacterial infection, host cells encounter numerous chemical stimuli, including bacterial pathogen-associated molecular patterns (PAMPs) such as lipopolysaccharide (LPS), bacterial toxins, and oxidative stress molecules. Recognition of these stimuli activates intracellular signalling pathways such as TLR, NOD-like receptor, NF-κB, and TNF signalling, leading to the production of antimicrobial molecules and inflammatory cytokines [74,75]. Therefore, the *N. rhizosphereae*-derived secondary metabolites may modulate multiple biological processes as defence mechanisms against excessive inflammation and oxidative damage caused by bacterial infections.

### 3.7. GO Cellular Component Enrichment

The GO cellular component enrichment analysis revealed 197 components. The most significantly predicted enriched component was localised to the cell surface with 20 targets, including TLR4 and HSP90AA1 (Figure 3B, Table S2). The enrichment of the cell surface suggested that the *N. rhizosphereae*-derived secondary metabolites may regulate the earliest stages of host–pathogen interactions by modulating receptor activation and inflammatory signalling. Such modulation could reduce excessive inflammatory responses [76]. The prominent representation of membrane-associated and extracellular components highlighted the potential importance of pathogen recognition, receptor signalling, and intercellular communication in mediating the host response to bacterial infection [77].

### 3.8. GO Molecular Function Enrichment

The GO molecular function enrichment analysis revealed 457 functions. The most significantly enriched term was identical protein binding, with 31 targets including hub genes HSP90AA1, RELA, PTGS2, ESR1, SIRT1, MMP9, PPARG, TLR4 (Figure 3C and Table S3). Many predicted proteins were shown to interact with copies of themselves (homodimerisation or oligomerisation). This process is essential for the activation of numerous signalling proteins involved in innate immunity [78]. Therefore, the secondary metabolites may interfere with these protein interactions, thereby suppressing bacterial-induced inflammatory signalling. The involvement of enzyme binding, protein homodimerisation, protein dimerisation, and kinase binding functions further supports a potential role for the secondary metabolites in modulating signalling complex assembly and phosphorylation cascades involved in inflammatory cytokine production [79,80].

### 3.9. KEGG Pathway Enrichment Analysis

In the KEGG pathway analysis, 156 KEGG terms were acquired (Table S4), and the top 10 terms were visualised based on the *p*-value (Figure 3D). The KEGG pathway enrichment analysis identified several significantly enriched pathways associated with immune and inflammatory responses. The most significantly enriched pathways included NOD-like receptor signalling (11 targets), followed by pathways in cancer with 16 targets and NF-κB with seven targets. The enrichment of NOD-like receptor signalling and NF-κB implied that these actinomycete-derived metabolites may modulate host inflammatory responses initiated following bacterial recognition. Since excessive activation of these pathways contributes to cytokine overproduction and tissue damage during bacterial infections, their regulation may complement the direct antibacterial effects of these antibacterial metabolites by limiting immunopathology while preserving host defence [74,81]. Although primarily associated with tumour generation, enrichment of the pathways in cancer regulates cell survival, inflammation, oxidative stress, and immune responses that are essential for host defence against bacterial infections [71,82]. Therefore, several enriched cancer-related pathways may primarily reflect the involvement of shared signalling molecules such as NF-κB, PI3K-Akt, MAPK, and apoptosis, which are central regulators of inflammation and immunity. Overall, the *N. rhizosphereae*-derived secondary metabolites have the potential to exert immunomodulatory action through interference with multiple pathways [83].

### 3.10. NF-κB Pathway

The NF-κB pathway is a host signalling pathway that many bacterial pathogens exploit to induce excessive inflammation, tissue damage, and immune dysregulation [68]. In this study, the KEGG pathway enrichment analysis identified the NF-κB signalling pathway as a major target of the predicted antibacterial compounds derived from actinomycetes. Several significant genes, highlighted in red, including TLR4, IL1B, IL1RAP, RIPK1, BIRC2 (cIAP1), BIRC3 (cIAP2), MALT1, NFκB1 (p50), RELA (p65), XIAP and PTGS2 (COX-2) were identified within the pathway (Figure 4). These genes participate in pathogen recognition, inflammatory signalling, apoptosis, and regulation of innate immunity, suggesting that the predicted targets of the actinomycete-derived metabolites are closely associated with the host response to bacterial infection [81]. Therefore, modulation of this pathway by actinomycete-derived metabolites may provide therapeutic antibacterial treatment by dampening excessive inflammatory responses while preserving protective innate immunity. Together with their demonstrated direct antibacterial activity, these findings suggested that the actinomycete-derived metabolites possess a dual mode of action, combining antimicrobial effects with host-directed immunomodulation [84]. This highlights their promise as candidates for the treatment of multidrug-resistant bacterial infections.

### 3.11. Functional Network

GeneMANIA analysis revealed that the query genes formed a highly interconnected antibacterial response network associated with host defence against bacterial infection (Figure 5). The majority of interactions were supported by physical interactions (pink, 56.90%), co-expression (purple, 22.64%) and predicted functional associations (orange, 18.54%), indicating that these proteins cooperate closely to coordinate the host’s defence against bacterial pathogens. Fewer interactions were based on shared pathways (blue, 1.93%). TLR4 constituted the central bacterial recognition receptor, initiating innate immune signalling that culminated in NF-κB activation through RELA and subsequent induction of PTGS2 (COX-2) expression, thereby promoting the production of pro-inflammatory mediators during bacterial infection [85,86,87]. Simultaneously, PPARG, NFE2L2 and SIRT1 constituted an antioxidant regulatory module that modulates oxidative stress generated during bacterial killing while preserving antimicrobial defence [88,89,90]. The addition of functionally related genes such as MYD88 innate immune signal transduction adaptor (MYD88), lymphocyte antigen 96 (LY96), macrophage receptor with collagenous structure (MARCO), kelch like ECH associated protein 1 (KEAP1), MAF bZIP transcription factor G (MAFG), versican (VCAN), NADPH oxidase 4 (NOX4), and PPARG coactivator 1 alpha (PPARGC1A), further reinforced the enrichment of innate immunity, inflammatory signalling, oxidative stress, and tissue repair, which are key biological processes activated during bacterial infection [91,92,93].

### 3.12. Docking

The top 10 core targets were selected for molecular docking with 10 potential antibacterial compounds to predict the probability of intermolecular interactions. Based on the literature, binding energies above −5 kcal/mol were classified as unfavourable interactions; those ranging from −5 to −7 kcal/mol were considered indicative of moderate binding affinity, whereas values ≤ −7 kcal/mol were interpreted as indicative of strong binding [94,95]. The majority of the actinomycete-derived metabolites exhibited favourable negative docking scores (ranging from –4.99 to −8.69 kcal/mol) against the selected targets, suggesting potentially stable ligand–protein interactions. Among the investigated metabolites, n-nonadecanol demonstrated the most favourable interaction with ESR1 (−8.69 kcal/mol), followed by strong predicted interactions with SIRT1 (−8.46 kcal/mol), HSP90AA1 (−8.26 kcal/mol) and NFE2L2 (−8.16 kcal/mol). Pentadecanoic acid also showed strong predicted interactions with SIRT1 and ESR1 (both −8.22 kcal/mol), while 2,6,11-trimethyldodecane demonstrated favourable interactions with ESR1 (−7.77 kcal/mol) and SIRT1 (−7.39 kcal/mol) (Figure 6). Moreover, the interaction analysis revealed different bonding patterns between these compounds and active amino acid side chains inside the binding pockets. The bonds included, among others, conventional hydrogen bonding, mutual polar contact, carbon–hydrogen bond, unfavourable donor–donor, pi-sulfur, pi-pi stacked, pi-alkyl and covalent interactions (Figure 7 and Figure S1). Overall, the docking results suggested that the actinomycete-derived metabolites may exert the multi-target characteristics predicted by the network-pharmacology analysis [65].

## 4. Conclusions

The *N. rhizosphereae*-derived secondary metabolites exhibited notable antibacterial activity, with low MIC values. They potentially exerted their antibacterial activity by disrupting cell membranes, resulting in loss of cytoplasmic contents. Moreover, network pharmacology, GO, and KEGG enrichments suggested that the compounds not only exert antibacterial activity directly by inhibiting bacteria but also through regulating host immunity via multi-target interactions and multi-pathway processes. However, future investigations should emphasise the experimental validation of the multi-target immunomodulatory mechanisms of these compounds.

## Figures and Tables

**Figure 1:**
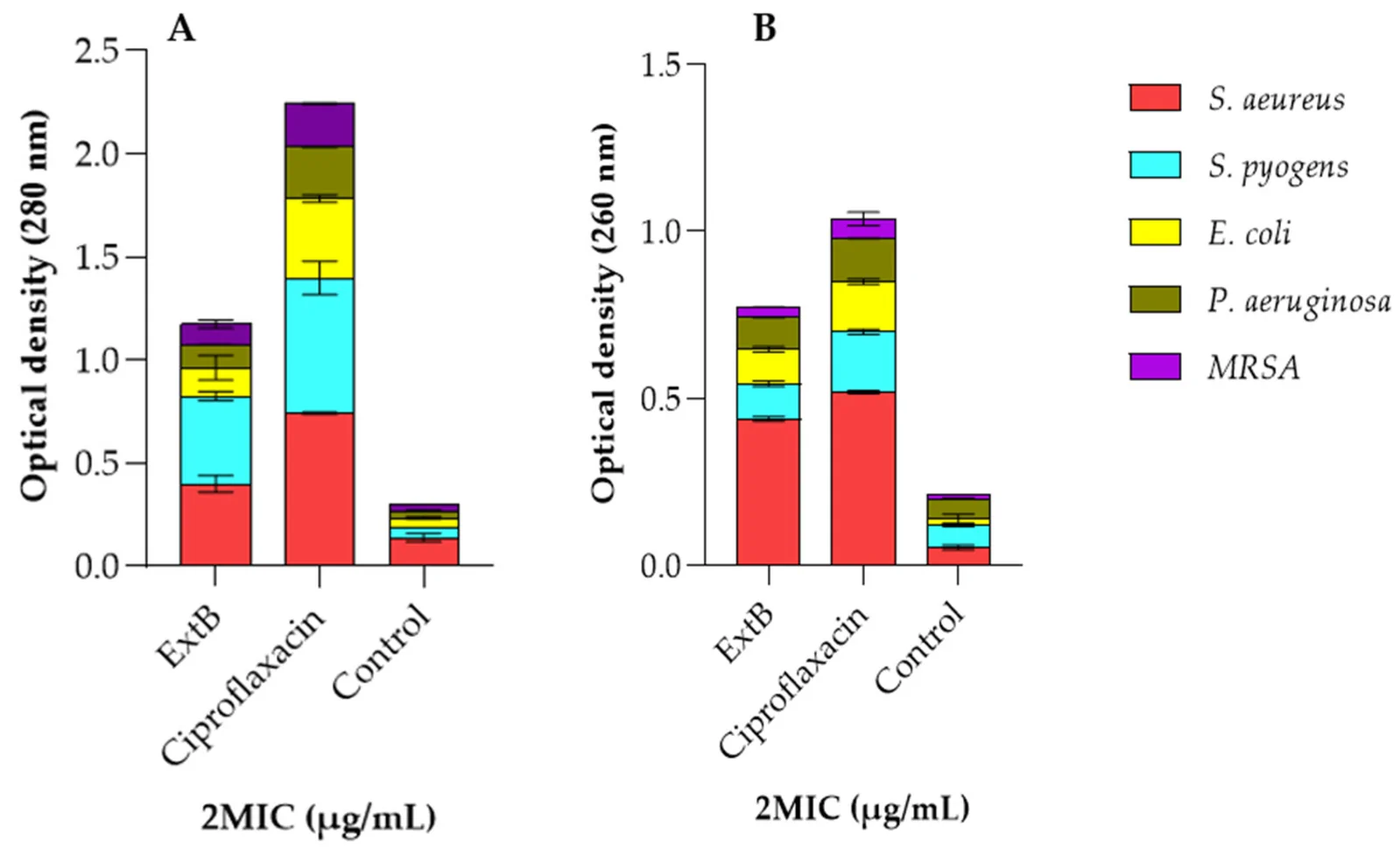
The effect of the extract on the release of 280 nm (**A**) and 260 nm (**B**) absorbing materials from bacterial strains.

**Figure 2:**
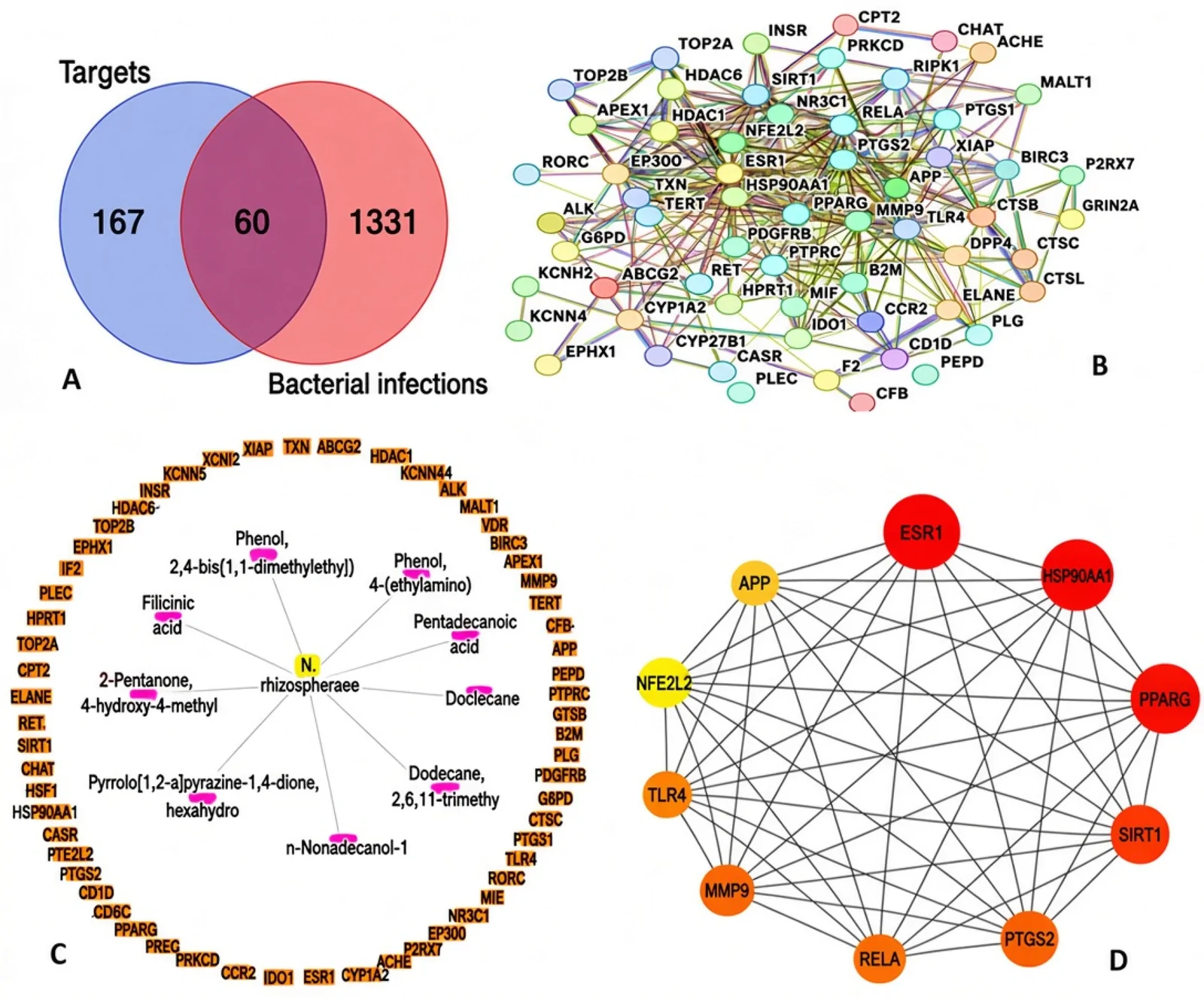
Network pharmacology results. (**A**) Venn diagram of targets versus bacterial infection targets; (**B**) PPI network of intersecting targets. Each node represents a protein, and the edge length shows the strength of the relationship between the targets. (**C**) Compound-intersecting target network. The yellow dot at the centre represents *N. rhizosphereae* (source of the compounds), the purple shapes illustrate the 10 identified antibacterial compounds, and the peach rectangles represent potential therapeutic targets. (**D**) Top ten hub targets. Node colour intensity represents degree value, with red indicating higher degree (more connections) and yellow indicating lower degree. The size of each node is proportional to its network connectivity. The lines (edges) denote interactions between the targets.

**Figure 3:**
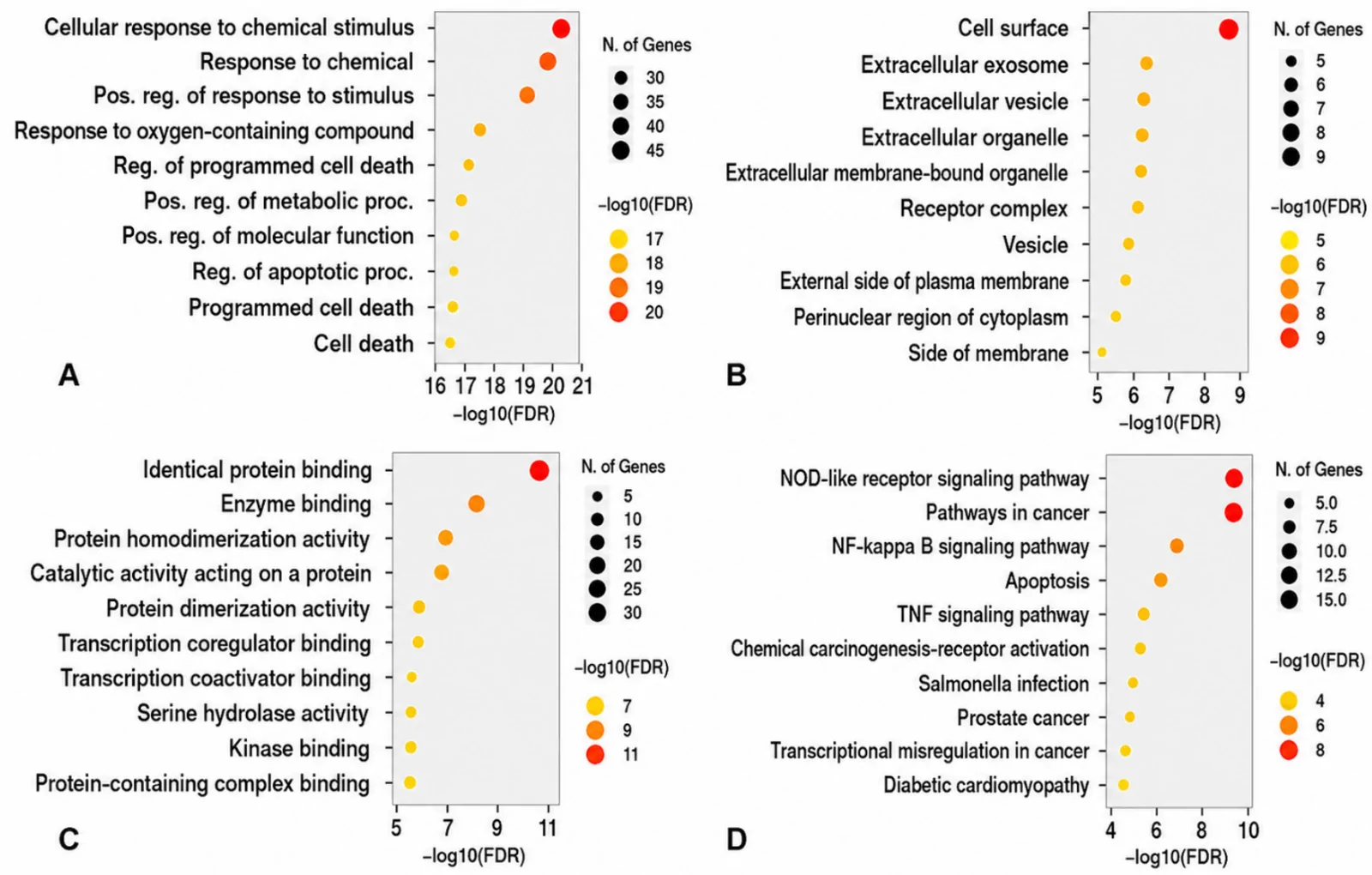
The GO and KEGG pathway enrichment analysis; (**A**) GO biological process enrichment, (**B**) GO cellular component enrichment, (**C**) GO molecular function enrichment and (**D**) KEGG pathway enrichment.

**Figure 4:**
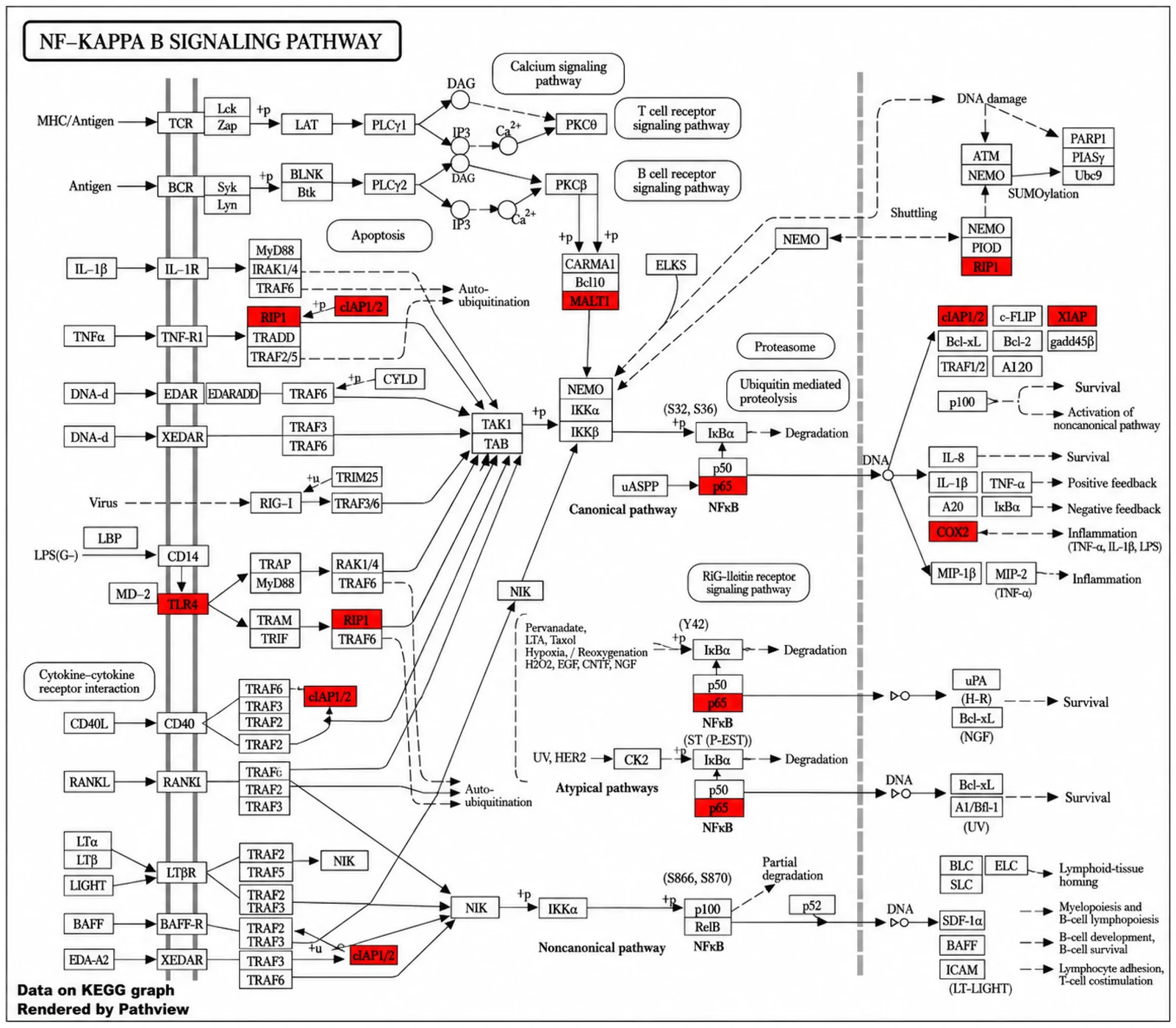
NF-κB signalling pathway. The genes highlighted in red represent potential molecular targets through which the compounds may exert their effects. The solid black arrows show the primary NF-κB activation routes and the dotted/grey lines are not the core NF-κB activation routes.

**Figure 5:**
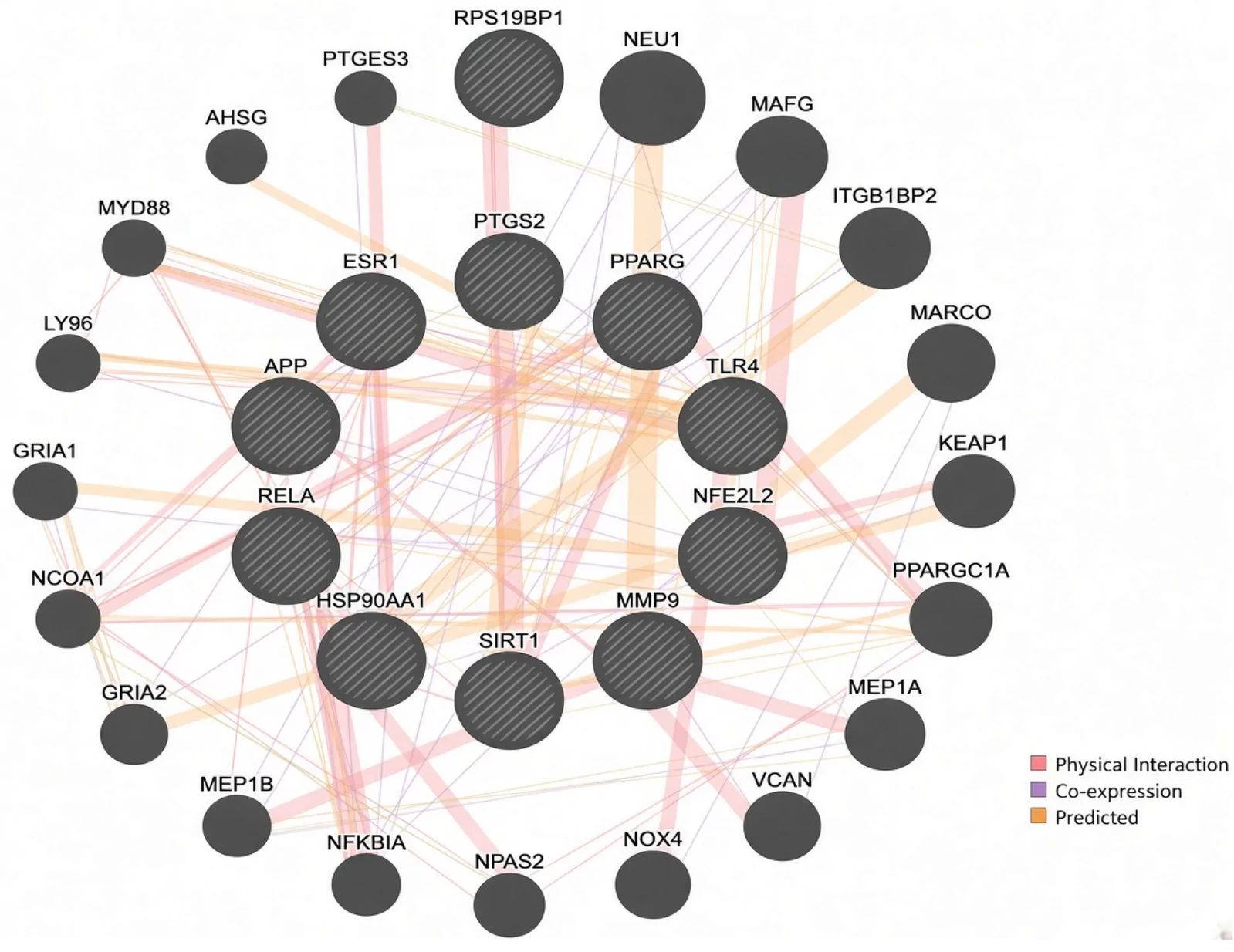
The co-expression network of common targets for compounds against bacterial infections. Circular nodes represent genes, and coloured edges represent different types of gene–gene interactions. Striped black nodes denote the submitted query genes, while solid black nodes represent genes functionally associated with the query genes. Larger nodes indicate genes with a higher number of interactions (greater connectivity) within the network.

**Figure 6:**
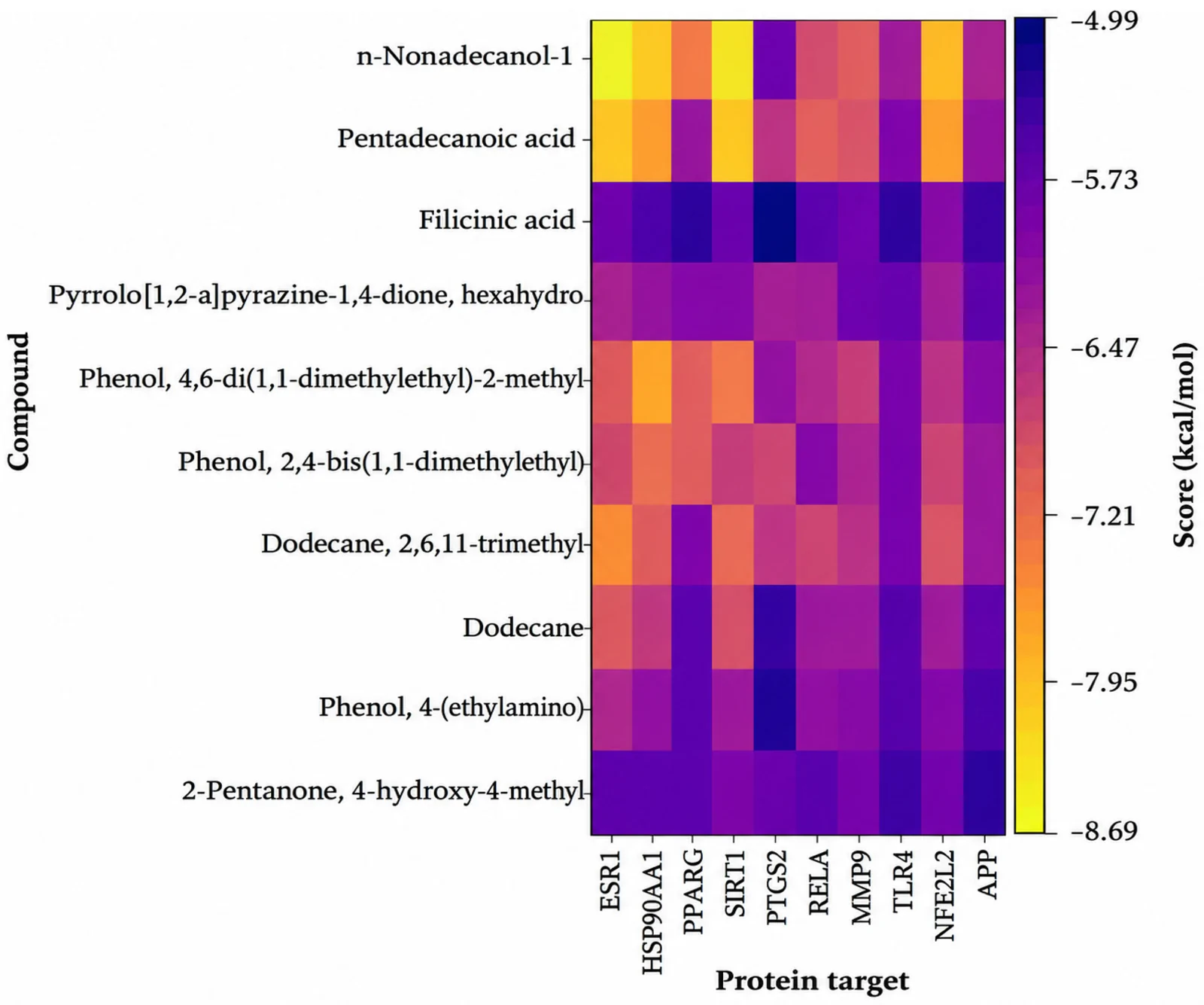
This heatmap summarises the molecular docking binding affinities (kcal/mol) of 10 ligands against 10 protein targets. The colour scale ranges from dark maroon (weaker binding; around −4.99 kcal/mol) to yellow (stronger binding; around −8.69 kcal/mol).

**Figure 7:**
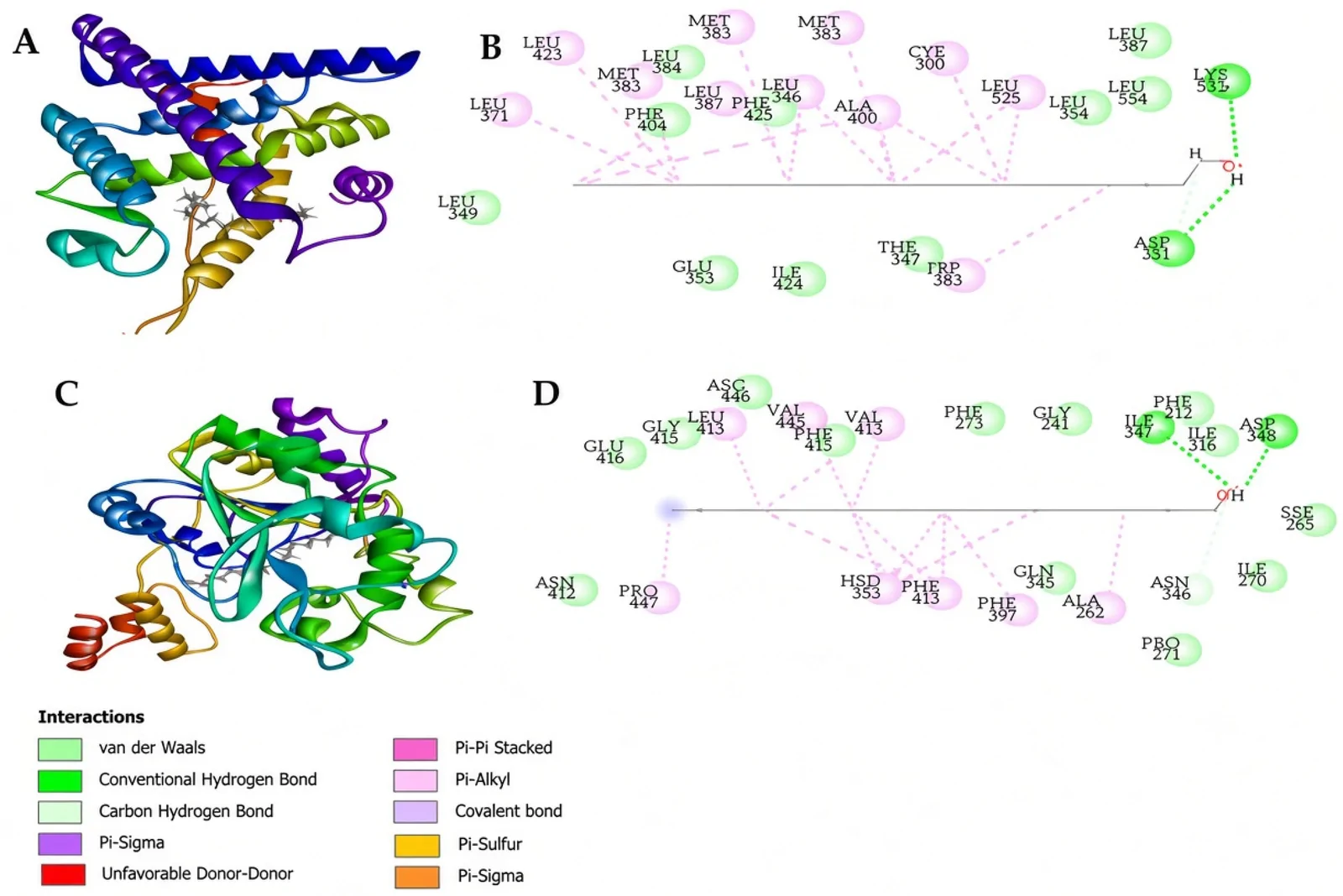
Visualisation analysis of the three- and two-dimensional (2 and 3D) structures of the docked complexes. (**A**) n-nonadecanol-ERS1 (3D), (**B**) n-nonadecanol-ERS1 (2D), (**C**) n-nonadecanol-SIRT1 (3D), and (**D**) n-nonadecanol-SIRT1 (2D).

**Table 1:** GC-MS identified compounds.

Peak No.	Name	Formula	Area (%)	Activity	Reference
1	2-Hexanol, 2-methyl-	C_7_H_16_O	0.91	–	
2	2-Pentanone, 4-hydroxy-4-methyl	C_6_H_12_O_2_	0.09	Antibacterial activity	[44]
3	3-Octanamine	C_8_H_19_N	0.27	–	
4	Oxirane, tetramethyl-	C_6_H_12_O	0.56	–	
5	2-Hexanol, 2-methyl-	C_7_H_16_O	25.40	–	
6	2-Pentanone, 4-amino-4-methyl-	C_6_H_13_NO	50.11	–	
7	Phenol, 4-(ethylamino)	C_8_H_11_NO	1.42	Antimicrobial activity	[45]
8	1,3-Methano-5bH-cyclobuta[cd]pentalen-5b-ol	C_10_H_14_O	1.49	–	
9	4-Piperidinone, 2,2,6,6-tetramethyl-	C_9_H_17_NO	0.44	–	
10	n-PROPYL NONYL ETHER	C_12_H_26_O	0.58	–	
11	Dodecane	C_12_H_26_		Antimicrobial activity	[46]
12	Dodecane, 2,6,11-trimethyl	C_15_H_32_	0.17	Antibacterial activity	[47]
13	Nonane, 5-butyl-	C_13_H_28_	0.12	–	
14	Benzene acetic acid, hexyl ester	C_14_H_20_O_2_	0.73	–	
15	1-Undecene, 4-methyl-	C_12_H_24_	0.28	–	
16	Phenol, 2,4-bis(1,1-dimethylethyl)	C_14_H_22_O	0.17	Antimicrobial activity	[48]
17	3-Ethyl-3-methylheptane		0.09		
18	Phenol, 4,6-di(1,1-dimethylethyl)-2-methyl	C_15_H_24_O	4.13	Antimicrobial activity	[49]
19	Benzene, (1-butyloctyl)-	C_18_H_30_	0.09	–	
20	Hexadecane, 4-methyl-	C_17_H_36_	0.22	–	
21	3-Methyl-1,4-diazabicyclo[4.3.0]nonan-2,5-dione N-acetyl-	C_10_H_14_N_2_O_3_	0.81	–	
22	Decane, 4-ethyl-	C_12_H_26_	0.22	–	
23	Pyrrolo[1,2-a]pyrazine-1,4-dione, hexahydro	C_7_H_10_N_2_O_2_	0.49	Antimicrobial activity	[50]
24	Filicinic acid	C_8_H_10_O_3_	1.74	Antimicrobial activity	[51]
25	Sulfurous acid, hexyl octyl ester	C_14_H_30_O_3_S	0.15	–	
26	7,9-Di-tert-butyl-1-oxaspiro(4,5)deca-6,9-diene-2,8-dione	C_17_H_24_O_3_	0.40	–	
27	Actinomycin C2	C_63_H_88_N_12_O_16_	1.86	Antibacterial activity	[52]
28	L-Proline, N-valeryl-, octadecyl ester	C_28_H_53_NO_3_	1.72	–	
29	Pentadecanoic acid	C_15_H_30_O_2_	2.85	Antibacterial activity	[53]
30	9-Eicosene, (E)-	C_20_H_40_	0.52	–	
31	2,3-Dimethyldodecane	C_14_H_30_	0.32	–	
32	n-Nonadecanol-1	C_19_H_40_O	1.33	Antimicrobial activity	[54]
33	2-Allyl-3-hydroxy-2-methylsuccinic acid, 1-ethyl	C_10_H_16_O_5_	0.19	–	

Key: – denotes no reported antimicrobial activity.

**Table 2:** Antibacterial activity of the *N. rhizosphereae*-derived extract.

Pathogen	Extract	
	Agar Well Diffusion (Zone of Inhibition (mm)	MIC (µg/mL)
*E. coli*	16 ± 0.3 ^a^	78.13
*P. aeruginosa*	14 ± 1.6 ^a^	78.13
*S. pyogenes*	18 ± 2.0 ^a,b^	39.06
*S. aureus*	23 ± 0.4 ^b^	19.55
*MRSA*	14 ± 0.8 ^a^	312.5

Inhibition zone diameter index: (≤10 mm) weak activity, (11–14 mm) moderate activity, (≥16 mm) strong activity. Different superscript letters (a, b) denote statistical significance at *p* < 0.05.

**Table 3:** Drug-likeness of the compounds.

Compound	Number of Violations of Lipinski’s Rules	Drug-Likeness
2-Pentanone, 4-hydroxy-4-methyl	0	Yes
Phenol, 4-(ethylamino)	0	Yes
Dodecane	1 violation: MLOGP > 4.15	Yes
Dodecane, 2,6,11-trimethyl-	1 violation: MLOGP > 4.15	Yes
Phenol, 2,4-bis(1,1-dimethylethyl)-	0	Yes
Phenol, 4,6-di(1,1-dimethylethyl)-2-methyl	0	Yes
Pyrrolo[1,2-a]pyrazine-1,4-dione, hexahydro	0	Yes
Filicinic acid	0	Yes
Actinomycin C2	>1	No
Pentadecanoic acid	0	Yes
n-Nonadecanol-1	1 violation: MLOGP > 4.15	Yes

## Data Availability

The original contributions presented in this study are included in the article/Supplementary Material. Further inquiries can be directed to the corresponding author.

## Supplementary Materials

The following supporting information can be downloaded at https://www.mdpi.com/article/10.3390/microorganisms14102179/s1, Figure S1: The formed interactions between the compounds and the key targets; Table S1: GO enrichment biological processes; Table S2: GO enrichment cellular components; Table S3: GO enrichment molecular function; Table S4: KEGG pathway enrichment.

## Abbreviations

The following abbreviations are used in this manuscript:

*N. rhizosphereae*	*Nocardia rhizosphereae*
GC–MS	Gas Chromatography–Mass Spectrometry
PPI	Protein–Protein Interaction
GO	Gene Ontology
KEGG	Kyoto Encyclopedia of Genes and Genomes
PDB	Protein Data Bank
STRING	Search Tool for the Retrieval of Interacting Genes/Proteins
ESR1	Estrogen Receptor 1
HSP90AA1	Heat Shock Protein 90 Alpha Family Class A Member 1
PPARG	Peroxisome proliferator-activated receptor gamma
SIRT1	Sirtuin 1
PTGS2	Prostaglandin-Endoperoxide Synthase 2
RELA	Proto-Oncogene, NF-κB Subunit
MMP9	Matrix Metallopeptidase 9
TLR4	Toll-like receptor 4
NFE2L2	Nuclear Factor, Erythroid 2-Like 2
APP	Amyloid Precursor Protein
