# Supplementary material for: Antibacterial and Multi-Target Immunomodulatory Mechanisms of Marine Nocardia rhizosphereae Metabolites: Experimental and Network Pharmacology
Source: Microorganisms. 2026 Sep 28;14(10):2179. doi: 10.3390/microorganisms14102179 (PMC13707996; doi:10.3390/microorganisms14102179)
Supplement: Supplementary file 1 [file microorganisms-14-02179-s001.zip › microorganisms-4547063-supplementary.pdf]

Table S1. GO enrichment biological processes.

Number of genes	Pathway
39	GO:0070887 Cellular response to chemical stimulus
47	GO:0042221 Response to chemical
39	GO:0048584 Positive regulation of response to stimulus
32	GO:1901700 Response to oxygen-containing compound
31	GO:0043067 Regulation of programmed cell death
42	GO:0009893 Positive regulation of metabolic process
30	GO:0042981 Regulation of apoptotic process
26	GO:0044093 Positive regulation of molecular function
33	GO:0008219 Cell death
33	GO:0012501 Programmed cell death
43	GO:0006950 Response to stress
32	GO:0006915 Apoptotic process
31	GO:0010647 Positive regulation of cell communication
31	GO:0023056 Positive regulation of signalling
38	GO:0051239 Regulation of multicellular organismal process
31	GO:0051240 Positive regulation of multicellular organismal process
43	GO:0048583 Regulation of response to stimulus
39	GO:0010604 Positive regulation of macromolecule metabolic process
32	GO:0008283 Cell population proliferation
26	GO:0032101 Regulation of response to external stimulus
29	GO:0065009 Regulation of molecular function
28	GO:0009967 Positive regulation of signal transduction
25	GO:1901701 Cellular response to oxygen-containing compound
36	GO:0031325 Positive regulation of cellular metabolic process
33	GO:0009605 Response to external stimulus
28	GO:0042127 Regulation of cell population proliferation
22	GO:0033993 Response to lipid
33	GO:0009891 Positive regulation of biosynthetic process
21	GO:0051050 Positive regulation of transport
19	GO:0031401 Positive regulation of protein modification process
23	GO:0051247 Positive regulation of protein metabolic process
36	GO:0023051 Regulation of signalling
36	GO:0010646 Regulation of cell communication
34	GO:0009966 Regulation of signal transduction
24	GO:0002684 Positive regulation of immune system process
34	GO:0002376 Immune system process
32	GO:0031328 Positive regulation of cellular biosynthetic process
22	GO:0031399 Regulation of protein modification process
16	GO:0050727 Regulation of inflammatory response

25	GO:0080134 Regulation of response to stress
19	GO:0032103 Positive regulation of response to external stimulus
26	GO:0042592 Homeostatic process
23	GO:0040011 Locomotion
31	GO:0010557 Positive regulation of macromolecule biosynthetic process
17	GO:0043065 Positive regulation of apoptotic process
23	GO:0010628 Positive regulation of gene expression
17	GO:0010562 Positive regulation of phosphorus metabolic process
27	GO:0044419 Biological process involved in interspecies interaction between organisms
17	GO:0045937 Positive regulation of phosphate metabolic process
25	GO:0051049 Regulation of transport
15	GO:2001233 Regulation of apoptotic signalling pathway
17	GO:0043068 Positive regulation of programmed cell death
20	GO:0006954 Inflammatory response
16	GO:0001934 Positive regulation of protein phosphorylation
27	GO:1902531 Regulation of intracellular signal transduction
16	GO:0042327 Positive regulation of phosphorylation
17	GO:0097190 Apoptotic signalling pathway
27	GO:0006952 Defence response
17	GO:0071396 Cellular response to lipid
18	GO:0002252 Immune effector process
19	GO:0031347 Regulation of defence response
26	GO:0032879 Regulation of localization
19	GO:0050778 Positive regulation of immune response
20	GO:0050790 Regulation of catalytic activity
20	GO:1901698 Response to nitrogen compound
16	GO:0001819 Positive regulation of cytokine production
27	GO:0006955 Immune response
20	GO:0009628 Response to abiotic stimulus
19	GO:0008284 Positive regulation of cell population proliferation
25	GO:0141124 Intracellular signalling cassette
25	GO:0002682 Regulation of immune system process
20	GO:1902533 Positive regulation of intracellular signal transduction
15	GO:1903131 Mononuclear cell differentiation
25	GO:0051246 Regulation of protein metabolic process
18	GO:0014070 Response to organic cyclic compound
30	GO:0035556 Intracellular signal transduction
34	GO:0048731 System development
19	GO:0040012 Regulation of locomotion
22	GO:0016477 Cell migration
18	GO:0019220 Regulation of phosphate metabolic process
18	GO:0051174 Regulation of phosphorus metabolic process
18	GO:0030334 Regulation of cell migration
29	GO:0065008 Regulation of biological quality

35	GO:0030154 Cell differentiation
35	GO:0048869 Cellular developmental process
12	GO:0062197 Cellular response to chemical stress
35	GO:0006810 Transport
11	GO:0051222 Positive regulation of protein transport
18	GO:0030097 Hemopoiesis
21	GO:0009719 Response to endogenous stimulus
20	GO:0050776 Regulation of immune response
18	GO:2000145 Regulation of cell motility
28	GO:0048468 Cell development
16	GO:0001932 Regulation of protein phosphorylation
15	GO:0002521 Leukocyte differentiation
13	GO:0043269 Regulation of monoatomic ion transport
17	GO:0001817 Regulation of cytokine production
17	GO:0001816 Cytokine production
23	GO:0033554 Cellular response to stress
9	GO:2001235 Positive regulation of apoptotic signalling pathway
12	GO:0002460 Adaptive immune response based on somatic recombination of immune receptors built from immunoglobulin superfamily domains
21	GO:0045595 Regulation of cell differentiation
7	GO:0070301 Cellular response to hydrogen peroxide
16	GO:0042325 Regulation of phosphorylation
20	GO:2000026 Regulation of multicellular organismal development
28	GO:0048513 Animal organ development
14	GO:0031349 Positive regulation of defence response
22	GO:0048870 Cell motility
10	GO:0072593 Reactive oxygen species metabolic process
12	GO:0030522 Intracellular receptor signalling pathway
14	GO:0040017 Positive regulation of locomotion
13	GO:0002443 Leukocyte mediated immunity
12	GO:0006979 Response to oxidative stress
15	GO:0009617 Response to bacterium
14	GO:0043408 Regulation of mapk cascade
23	GO:0045935 Positive regulation of nucleobase-containing compound metabolic process
11	GO:1904951 Positive regulation of establishment of protein localization
26	GO:0009056 Catabolic process
16	GO:0009725 Response to hormone
14	GO:0002250 Adaptive immune response
25	GO:0050793 Regulation of developmental process
13	GO:0070201 Regulation of establishment of protein localization
18	GO:0071495 Cellular response to endogenous stimulus
13	GO:0071407 Cellular response to organic cyclic compound
12	GO:0051223 Regulation of protein transport
14	GO:0043085 Positive regulation of catalytic activity
21	GO:0048585 Negative regulation of response to stimulus

18	GO:0006468 Protein phosphorylation
21	GO:0051707 Response to other organism
21	GO:0043207 Response to external biotic stimulus
12	GO:0043410 Positive regulation of MAPK cascade
13	GO:0030335 Positive regulation of cell migration
17	GO:0045321 Leukocyte activation
18	GO:0001775 Cell activation
21	GO:0009607 Response to biotic stimulus
14	GO:0018193 Peptidyl-amino acid modification
11	GO:0002237 Response to molecule of bacterial origin
17	GO:0051241 Negative regulation of multicellular organismal process
14	GO:0006897 Endocytosis
13	GO:2000147 Positive regulation of cell motility
8	GO:0034614 Cellular response to reactive oxygen species
24	GO:0006796 Phosphate-containing compound metabolic process
8	GO:0048660 Regulation of smooth muscle cell proliferation
24	GO:0006793 Phosphorus metabolic process
26	GO:0009892 Negative regulation of metabolic process
7	GO:0042542 Response to hydrogen peroxide
12	GO:1903829 Positive regulation of protein localization
25	GO:0010605 Negative regulation of macromolecule metabolic process
17	GO:0010629 Negative regulation of gene expression
7	GO:0048661 Positive regulation of smooth muscle cell proliferation
14	GO:0000165 Mapk cascade
8	GO:0048659 Smooth muscle cell proliferation
18	GO:0016310 Phosphorylation
13	GO:0032787 Monocarboxylic acid metabolic process
9	GO:0009755 Hormone-mediated signaling pathway
13	GO:0032870 Cellular response to hormone stimulus
10	GO:0002699 Positive regulation of immune effector process
15	GO:0046649 Lymphocyte activation
15	GO:0098657 Import into cell
10	GO:0007249 Canonical NF-Kappab signal transduction
7	GO:0032963 Collagen metabolic process
8	GO:0002821 Positive regulation of adaptive immune response
6	GO:0030225 Macrophage differentiation
13	GO:1901699 Cellular response to nitrogen compound
11	GO:0034762 Regulation of transmembrane transport
15	GO:0043069 Negative regulation of programmed cell death
18	GO:0006629 Lipid metabolic process
8	GO:0002456 T cell mediated immunity
14	GO:0019725 Cellular homeostasis
25	GO:0007166 Cell surface receptor signalling pathway
9	GO:0034599 Cellular response to oxidative stress

22	GO:0010558 Negative regulation of macromolecule biosynthetic process
9	GO:0033674 Positive regulation of kinase activity
25	GO:0036211 Protein modification process
10	GO:0046651 Lymphocyte proliferation
8	GO:0141193 Nuclear receptor-mediated signalling pathway
11	GO:0030099 Myeloid cell differentiation
10	GO:0032496 Response to lipopolysaccharide
13	GO:0070848 Response to growth factor
9	GO:0002440 Production of molecular mediator of immune response
13	GO:1902532 Negative regulation of intracellular signal transduction
10	GO:0032943 Mononuclear cell proliferation
22	GO:0031327 Negative regulation of cellular biosynthetic process
8	GO:0034764 Positive regulation of transmembrane transport
8	GO:0000302 Response to reactive oxygen species
22	GO:0009890 Negative regulation of biosynthetic process
13	GO:0050865 Regulation of cell activation
23	GO:0031324 Negative regulation of cellular metabolic process
12	GO:0022407 Regulation of cell-cell adhesion
8	GO:0043270 Positive regulation of monoatomic ion transport
14	GO:0032880 Regulation of protein localization
14	GO:0043066 Negative regulation of apoptotic process
8	GO:0033002 Muscle cell proliferation
10	GO:0070661 Leukocyte proliferation
10	GO:0002449 Lymphocyte mediated immunity
8	GO:0001909 Leukocyte mediated cytotoxicity
7	GO:0050714 Positive regulation of protein secretion
25	GO:0043412 Macromolecule modification
9	GO:0043122 Regulation of canonical NF-Kappab signal transduction
9	GO:0051347 Positive regulation of transferase activity
12	GO:0002253 Activation of immune response
9	GO:0034765 Regulation of monoatomic ion transmembrane transport
12	GO:0007610 Behaviour
11	GO:0098771 Inorganic ion homeostasis
7	GO:2000377 Regulation of reactive oxygen species metabolic process
23	GO:0009653 Anatomical structure morphogenesis
12	GO:0071363 Cellular response to growth factor stimulus
17	GO:0098542 Defence response to other organism
10	GO:0009410 Response to xenobiotic stimulus
6	GO:0001913 T cell mediated cytotoxicity
6	GO:1900182 Positive regulation of protein localization to nucleus
8	GO:0002700 Regulation of production of molecular mediator of immune response
18	GO:0006508 Proteolysis
10	GO:0009314 Response to radiation
6	GO:0150076 Neuroinflammatory response

13	GO:0048871 Multicellular organismal-level homeostasis
8	GO:2001234 Negative regulation of apoptotic signalling pathway
7	GO:0002824 Positive regulation of adaptive immune response based on somatic recombination of immune receptors built from immunoglobulin superfamily domains
10	GO:0006816 Calcium ion transport
14	GO:0060341 Regulation of cellular localization
10	GO:0002696 Positive regulation of leukocyte activation
7	GO:0002367 Cytokine production involved in immune response
7	GO:0002718 Regulation of cytokine production involved in immune response
8	GO:0045860 Positive regulation of protein kinase activity
18	GO:1902680 Positive regulation of RNA biosynthetic process
9	GO:0044403 Biological process involved in symbiotic interaction
13	GO:0060284 Regulation of cell development
9	GO:0048545 Response to steroid hormone
16	GO:0051094 Positive regulation of developmental process
28	GO:0019219 Regulation of nucleobase-containing compound metabolic process
7	GO:2000116 Regulation of cysteine-type endopeptidase activity
18	GO:0022008 Neurogenesis
14	GO:0048878 Chemical homeostasis
7	GO:0002702 Positive regulation of production of molecular mediator of immune response
7	GO:0018105 Peptidyl-serine phosphorylation
7	GO:0050729 Positive regulation of inflammatory response
10	GO:0002697 Regulation of immune effector process
8	GO:0002819 Regulation of adaptive immune response
10	GO:0050867 Positive regulation of cell activation
11	GO:0055080 Monoatomic cation homeostasis
9	GO:0042063 Gliogenesis
10	GO:1903037 Regulation of leukocyte cell-cell adhesion
7	GO:0015718 Monocarboxylic acid transport
13	GO:0045597 Positive regulation of cell differentiation
11	GO:0050801 Monoatomic ion homeostasis
8	GO:0010001 Glial cell differentiation
9	GO:1903039 Positive regulation of leukocyte cell-cell adhesion
6	GO:0033559 Unsaturated fatty acid metabolic process
7	GO:0018209 Peptidyl-serine modification
13	GO:0019752 Carboxylic acid metabolic process
13	GO:0030155 Regulation of cell adhesion
9	GO:0010959 Regulation of metal ion transport
19	GO:0009888 Tissue development
14	GO:0035295 Tube development
13	GO:0043436 Oxoacid metabolic process
6	GO:0002720 Positive regulation of cytokine production involved in immune response
13	GO:0006082 Organic acid metabolic process
4	GO:0060765 Regulation of androgen receptor signalling pathway

6	GO:0030518 Nuclear receptor-mediated steroid hormone signaling pathway
6	GO:0032760 Positive regulation of tumour necrosis factor production
18	GO:0051254 Positive regulation of rna metabolic process
10	GO:0007159 Leukocyte cell-cell adhesion
6	GO:0006690 Icosanoid metabolic process
8	GO:1903532 Positive regulation of secretion by cell
6	GO:0071887 Leukocyte apoptotic process
6	GO:1903557 Positive regulation of tumour necrosis factor superfamily cytokine production
5	GO:0033138 Positive regulation of peptidyl-serine phosphorylation
5	GO:2000379 Positive regulation of reactive oxygen species metabolic process
6	GO:0090398 Cellular senescence
15	GO:0006811 Monoatomic ion transport
16	GO:0023057 Negative regulation of signalling
16	GO:0010648 Negative regulation of cell communication
4	GO:0001516 Prostaglandin biosynthetic process
4	GO:0046457 Prostanoid biosynthetic process
14	GO:0051130 Positive regulation of cellular component organization
11	GO:0002694 Regulation of leukocyte activation
8	GO:0006874 Intracellular calcium ion homeostasis
9	GO:0051251 Positive regulation of lymphocyte activation
17	GO:0045893 Positive regulation of DNA-templated transcription
21	GO:0007399 Nervous system development
7	GO:0050920 Regulation of chemotaxis
6	GO:0034767 Positive regulation of monoatomic ion transmembrane transport
8	GO:0001906 Cell killing
8	GO:0009416 Response to light stimulus
8	GO:0051047 Positive regulation of secretion
8	GO:0062012 Regulation of small molecule metabolic process
15	GO:0140546 Defence response to symbiont
7	GO:0052548 Regulation of endopeptidase activity
10	GO:0002757 Immune response-activating signalling pathway
15	GO:0007267 Cell-cell signalling
9	GO:0022409 Positive regulation of cell-cell adhesion
20	GO:0003008 System process
9	GO:0006869 Lipid transport
15	GO:0009968 Negative regulation of signal transduction
6	GO:0002709 Regulation of t cell mediated immunity
5	GO:0034605 Cellular response to heat
4	GO:0035872 Nucleotide-binding domain leucine rich repeat containing receptor signalling pathway
6	GO:0043401 Steroid hormone receptor signalling pathway
9	GO:0043549 Regulation of kinase activity
4	GO:0051968 Positive regulation of synaptic transmission glutamatergic
6	GO:1900180 Regulation of protein localization to nucleus

9	GO:0032102 Negative regulation of response to external stimulus
12	GO:0035239 Tube morphogenesis
8	GO:0055074 Calcium ion homeostasis
6	GO:0062013 Positive regulation of small molecule metabolic process
7	GO:0051091 Positive regulation of DNA-binding transcription factor activity
5	GO:0032370 Positive regulation of lipid transport
20	GO:0051128 Regulation of cellular component organization
12	GO:0030001 Metal ion transport
10	GO:0030162 Regulation of proteolysis
6	GO:0009411 Response to UV
5	GO:1902893 Regulation of mirna transcription
8	GO:0010038 Response to metal ion
16	GO:0055085 Transmembrane transport
17	GO:0044281 Small molecule metabolic process
10	GO:0045785 Positive regulation of cell adhesion
5	GO:0061614 Mirna transcription
7	GO:0002822 Regulation of adaptive immune response based on somatic recombination of immune receptors built from immunoglobulin superfamily domains
5	GO:0032731 Positive regulation of interleukin-1 beta production
10	GO:0044283 Small molecule biosynthetic process
4	GO:0042307 Positive regulation of protein import into nucleus
7	GO:0097191 Extrinsic apoptotic signalling pathway
7	GO:0052547 Regulation of peptidase activity
10	GO:0051249 Regulation of lymphocyte activation
9	GO:0010720 Positive regulation of cell development
13	GO:0006812 Monoatomic cation transport
10	GO:0044092 Negative regulation of molecular function
7	GO:0006909 Phagocytosis
9	GO:0002833 Positive regulation of response to biotic stimulus
11	GO:0051248 Negative regulation of protein metabolic process
4	GO:0061081 Positive regulation of myeloid leukocyte cytokine production involved in immune response
4	GO:0150077 Regulation of neuroinflammatory response
7	GO:0018108 Peptidyl-tyrosine phosphorylation
7	GO:1901136 Carbohydrate derivative catabolic process
7	GO:0018212 Peptidyl-tyrosine modification
7	GO:0051924 Regulation of calcium ion transport
7	GO:0002573 Myeloid leukocyte differentiation
12	GO:0140352 Export from cell
5	GO:0033273 Response to vitamin
10	GO:0002831 Regulation of response to biotic stimulus
6	GO:0019362 Pyridine nucleotide metabolic process
6	GO:0046496 Nicotinamide nucleotide metabolic process
4	GO:0006476 Protein deacetylation
3	GO:0014004 Microglia differentiation

11	GO:0008610 Lipid biosynthetic process
9	GO:0006935 Chemotaxis
12	GO:0051093 Negative regulation of developmental process
9	GO:0010876 Lipid localization
13	GO:0098609 Cell-cell adhesion
6	GO:2001236 Regulation of extrinsic apoptotic signalling pathway
9	GO:0042330 Taxis
8	GO:0051090 Regulation of DNA-binding transcription factor activity
5	GO:2000628 Regulation of mirna metabolic process
9	GO:0031667 Response to nutrient levels
6	GO:0006109 Regulation of carbohydrate metabolic process
5	GO:0033135 Regulation of peptidyl-serine phosphorylation
6	GO:0072524 Pyridine-containing compound metabolic process
5	GO:0032637 Interleukin-8 production
5	GO:0032677 Regulation of interleukin-8 production
5	GO:0032732 Positive regulation of interleukin-1 production
7	GO:0050708 Regulation of protein secretion
9	GO:0051338 Regulation of transferase activity
6	GO:0048771 Tissue remodelling
5	GO:0002711 Positive regulation of t cell mediated immunity
12	GO:0007167 Enzyme-linked receptor protein signaling pathway
4	GO:0030574 Collagen catabolic process
4	GO:0045429 Positive regulation of nitric oxide biosynthetic process
7	GO:0002274 Myeloid leukocyte activation
7	GO:1904062 Regulation of monoatomic cation transmembrane transport
6	GO:0007565 Female pregnancy
4	GO:1904407 Positive regulation of nitric oxide metabolic process
5	GO:1905954 Positive regulation of lipid localization
4	GO:0006636 Unsaturated fatty acid biosynthetic process
4	GO:0006693 Prostaglandin metabolic process
4	GO:0050856 Regulation of t cell receptor signalling pathway
7	GO:0042113 B cell activation
11	GO:0099536 Synaptic signalling
9	GO:0030003 Intracellular monoatomic cation homeostasis
4	GO:0006692 Prostanoid metabolic process
4	GO:0030521 Androgen receptor signalling pathway
8	GO:0006631 Fatty acid metabolic process
9	GO:0006873 Intracellular monoatomic ion homeostasis
5	GO:0001676 Long-chain fatty acid metabolic process
4	GO:0035601 Protein deacylation
9	GO:0045088 Regulation of innate immune response
5	GO:2001243 Negative regulation of intrinsic apoptotic signaling pathway
5	GO:0035249 Synaptic transmission glutamatergic
4	GO:0006110 Regulation of glycolytic process

4	GO:0030811 Regulation of nucleotide catabolic process
4	GO:0033121 Regulation of purine nucleotide catabolic process
4	GO:0050922 Negative regulation of chemotaxis
6	GO:0051897 Positive regulation of phosphatidylinositol 3-kinase/protein kinase b signal transduction
7	GO:0001666 Response to hypoxia
10	GO:0042110 T cell activation
8	GO:0045859 Regulation of protein kinase activity
9	GO:0044057 Regulation of system process
5	GO:0046822 Regulation of nucleocytoplasmic transport
12	GO:1901652 Response to peptide
7	GO:0051402 Neuron apoptotic process
5	GO:0010586 Mirna metabolic process
6	GO:0032640 Tumour necrosis factor production
6	GO:0032680 Regulation of tumour necrosis factor production
6	GO:0070374 Positive regulation of erk1 and erk2 cascade
3	GO:0071318 Cellular response to ATP
23	GO:0051641 Cellular localization
4	GO:0060135 Maternal process involved in female pregnancy
4	GO:0061082 Myeloid leukocyte cytokine production
7	GO:0036293 Response to decreased oxygen levels
5	GO:0043405 Regulation of map kinase activity
12	GO:0044255 Cellular lipid metabolic process
24	GO:0051252 Regulation of RNA metabolic process
6	GO:0071706 Tumour necrosis factor superfamily cytokine production
6	GO:1903555 Regulation of tumour necrosis factor superfamily cytokine production
5	GO:0015908 Fatty acid transport
4	GO:0019369 Arachidonate metabolic process
3	GO:0032310 Prostaglandin secretion
4	GO:0042306 Regulation of protein import into nucleus
9	GO:1903530 Regulation of secretion by cell
23	GO:2001141 Regulation of RNA biosynthetic process
10	GO:0055082 Intracellular chemical homeostasis
12	GO:0007417 Central nervous system development
5	GO:0009408 Response to heat
5	GO:0051928 Positive regulation of calcium ion transport
6	GO:0044703 Multi-organism reproductive process
4	GO:0046456 Icosanoid biosynthetic process
8	GO:0050900 Leukocyte migration
9	GO:0010817 Regulation of hormone levels
2	GO:2000471 Regulation of hematopoietic stem cell migration
2	GO:2000473 Positive regulation of hematopoietic stem cell migration
6	GO:0044706 Multi-multicellular organism process
4	GO:1902895 Positive regulation of mirna transcription
7	GO:0002703 Regulation of leukocyte mediated immunity

5	GO:1904064 Positive regulation of cation transmembrane transport
7	GO:0034504 Protein localization to nucleus
7	GO:0097193 Intrinsic apoptotic signalling pathway
6	GO:0009612 Response to mechanical stimulus
12	GO:0009894 Regulation of catabolic process
13	GO:0045944 Positive regulation of transcription by RNA polymerase ii
5	GO:0009154 Purine ribonucleotide catabolic process
5	GO:0032611 Interleukin-1 beta production
5	GO:0032651 Regulation of interleukin-1 beta production
5	GO:0042116 Macrophage activation
4	GO:0043030 Regulation of macrophage activation
4	GO:2001238 Positive regulation of extrinsic apoptotic signalling pathway
3	GO:0036120 Cellular response to platelet-derived growth factor stimulus
12	GO:0030163 Protein catabolic process
4	GO:0043470 Regulation of carbohydrate catabolic process
4	GO:0046824 Positive regulation of nucleocytoplasmic transport
9	GO:0003013 Circulatory system process
21	GO:0033036 Macromolecule localization
8	GO:0030098 Lymphocyte differentiation
15	GO:0007155 Cell adhesion
7	GO:0019216 Regulation of lipid metabolic process
8	GO:0045089 Positive regulation of innate immune response
7	GO:0030217 T cell differentiation
6	GO:1901654 Response to ketone
14	GO:0009057 Macromolecule catabolic process
5	GO:0032368 Regulation of lipid transport
3	GO:0070424 Regulation of nucleotide-binding domain leucine rich repeat containing receptor signalling pathway
7	GO:0070482 Response to oxygen levels
6	GO:0072330 Monocarboxylic acid biosynthetic process
4	GO:0045058 T cell selection
6	GO:0071383 Cellular response to steroid hormone stimulus
5	GO:0009261 Ribonucleotide catabolic process
6	GO:0043123 Positive regulation of canonical NF-Kappab signal transduction
5	GO:0051702 Biological process involved in interaction with symbiont
7	GO:0071214 Cellular response to abiotic stimulus
7	GO:0104004 Cellular response to environmental stimulus
8	GO:0007005 Mitochondrion organization
4	GO:0045428 Regulation of nitric oxide biosynthetic process
4	GO:2000630 Positive regulation of mirna metabolic process
3	GO:0036119 Response to platelet-derived growth factor
5	GO:0006195 Purine nucleotide catabolic process
4	GO:0001916 Positive regulation of t cell mediated cytotoxicity
4	GO:0033143 Regulation of intracellular steroid hormone receptor signalling pathway
4	GO:0098732 Macromolecule deacylation

7	GO:0048872 Homeostasis of number of cells
12	GO:0072359 Circulatory system development
4	GO:0043627 Response to estrogen
4	GO:0050854 Regulation of antigen receptor-mediated signalling pathway
4	GO:0080164 Regulation of nitric oxide metabolic process
7	GO:0050870 Positive regulation of t cell activation
5	GO:0071456 Cellular response to hypoxia
3	GO:0030813 Positive regulation of nucleotide catabolic process
3	GO:0033123 Positive regulation of purine nucleotide catabolic process
3	GO:0045821 Positive regulation of glycolytic process
10	GO:0007268 Chemical synaptic transmission
5	GO:0043281 Regulation of cysteine-type endopeptidase activity involved in apoptotic process
10	GO:0098916 Anterograde trans-synaptic signalling
9	GO:0051046 Regulation of secretion
4	GO:1900076 Regulation of cellular response to insulin stimulus
7	GO:0002218 Activation of innate immune response
5	GO:0014074 Response to purine-containing compound
5	GO:0071902 Positive regulation of protein serine/threonine kinase activity
8	GO:0016032 Viral process
4	GO:0043124 Negative regulation of canonical NF-Kappab signal transduction
5	GO:0051092 Positive regulation of NF-Kappab transcription factor activity
10	GO:0099537 Trans-synaptic signalling
7	GO:0043086 Negative regulation of catalytic activity
2	GO:0010041 Response to iron(iii) ion
3	GO:0046697 Decidualization
3	GO:2000273 Positive regulation of signaling receptor activity
3	GO:2000773 Negative regulation of cellular senescence
4	GO:0022617 Extracellular matrix disassembly
7	GO:0046942 Carboxylic acid transport
8	GO:0050863 Regulation of t cell activation
6	GO:0071222 Cellular response to lipopolysaccharide
7	GO:0015849 Organic acid transport
7	GO:0002429 Immune response-activating cell surface receptor signalling pathway
4	GO:1903578 Regulation of ATP metabolic process
6	GO:0019722 Calcium-mediated signalling
5	GO:0036294 Cellular response to decreased oxygen levels
5	GO:0001890 Placenta development
5	GO:0032612 Interleukin-1 production
5	GO:0032652 Regulation of interleukin-1 production
3	GO:0050857 Positive regulation of antigen receptor-mediated signalling pathway
3	GO:0070431 Nucleotide-binding oligomerization domain containing 2 signalling pathway
4	GO:2000772 Regulation of cellular senescence
6	GO:0071900 Regulation of protein serine/threonine kinase activity

4	GO:0045685 Regulation of glial cell differentiation
6	GO:0046434 Organophosphate catabolic process
5	GO:0033157 Regulation of intracellular protein transport
8	GO:0090066 Regulation of anatomical structure size
6	GO:0071219 Cellular response to molecule of bacterial origin
4	GO:0043406 Positive regulation of map kinase activity
5	GO:0072523 Purine-containing compound catabolic process
12	GO:0045087 Innate immune response
4	GO:0045913 Positive regulation of carbohydrate metabolic process
22	GO:0006355 Regulation of DNA-templated transcription
11	GO:0046903 Secretion
5	GO:0097696 Cell surface receptor signalling pathway via stat
6	GO:0034612 Response to tumour necrosis factor
6	GO:0071674 Mononuclear cell migration
5	GO:0038061 Non-canonical NF-Kappab signal transduction
14	GO:0022414 Reproductive process
11	GO:0034097 Response to cytokine
4	GO:0006809 Nitric oxide biosynthetic process
4	GO:0051966 Regulation of synaptic transmission glutamatergic
10	GO:0002764 Immune response-regulating signalling pathway
6	GO:0002706 Regulation of lymphocyte mediated immunity
6	GO:0042098 T cell proliferation
6	GO:0042445 Hormone metabolic process
4	GO:0032757 Positive regulation of interleukin-8 production
5	GO:0043409 Negative regulation of MAPK cascade
5	GO:0009166 Nucleotide catabolic process
3	GO:0015732 Prostaglandin transport
3	GO:0034391 Regulation of smooth muscle cell apoptotic process
3	GO:0071404 Cellular response to low-density lipoprotein particle stimulus
3	GO:1904996 Positive regulation of leukocyte adhesion to vascular endothelial cell
7	GO:0009306 Protein secretion
4	GO:0070227 Lymphocyte apoptotic process
4	GO:1900542 Regulation of purine nucleotide metabolic process
5	GO:0071453 Cellular response to oxygen levels
12	GO:0048646 Anatomical structure formation involved in morphogenesis
4	GO:0006140 Regulation of nucleotide metabolic process
7	GO:0035592 Establishment of protein localization to extracellular region
4	GO:1901224 Positive regulation of non-canonical NF-Kappab signal transduction
3	GO:0034390 Smooth muscle cell apoptotic process
3	GO:0055094 Response to lipoprotein particle
5	GO:0006633 Fatty acid biosynthetic process
14	GO:0016192 Vesicle-mediated transport
7	GO:0071692 Protein localization to extracellular region
8	GO:0051336 Regulation of hydrolase activity

4	GO:0001914 Regulation of t cell mediated cytotoxicity
4	GO:0002224 Toll-like receptor signalling pathway
4	GO:0010657 Muscle cell apoptotic process
3	GO:0033198 Response to ATP
6	GO:0051896 Regulation of phosphatidylinositol 3-kinase/protein kinase b signal transduction
10	GO:0032940 Secretion by cell
5	GO:0007584 Response to nutrient
12	GO:0060429 Epithelium development
5	GO:1905952 Regulation of lipid localization
6	GO:0071216 Cellular response to biotic stimulus
3	GO:0034405 Response to fluid shear stress
3	GO:0060907 Positive regulation of macrophage cytokine production
3	GO:0090594 Inflammatory response to wounding
9	GO:0045596 Negative regulation of cell differentiation
4	GO:0034644 Cellular response to UV
4	GO:0046209 Nitric oxide metabolic process
4	GO:2001057 Reactive nitrogen species metabolic process
13	GO:0015031 Protein transport
3	GO:0010574 Regulation of vascular endothelial growth factor production
3	GO:0060603 Mammary gland duct morphogenesis
2	GO:1900122 Positive regulation of receptor binding
4	GO:0046635 Positive regulation of alpha-beta t cell activation
6	GO:0070372 Regulation of erk1 and erk2 cascade
4	GO:0046364 Monosaccharide biosynthetic process
11	GO:0034220 Monoatomic ion transmembrane transport
3	GO:0001893 Maternal placenta development
3	GO:0032743 Positive regulation of interleukin-2 production
22	GO:0006351 Dna-templated transcription
5	GO:0050671 Positive regulation of lymphocyte proliferation
6	GO:0007611 Learning or memory
4	GO:1904892 Regulation of receptor signalling pathway via stat
4	GO:0010906 Regulation of glucose metabolic process
4	GO:0043255 Regulation of carbohydrate biosynthetic process
4	GO:0061515 Myeloid cell development
4	GO:0090316 Positive regulation of intracellular protein transport
5	GO:2001242 Regulation of intrinsic apoptotic signalling pathway
6	GO:0050670 Regulation of lymphocyte proliferation
3	GO:0001975 Response to amphetamine
3	GO:0071295 Cellular response to vitamin
3	GO:1903580 Positive regulation of ATP metabolic process
3	GO:1990806 Ligand-gated ion channel signalling pathway
5	GO:0007254 Jnk cascade
5	GO:0032946 Positive regulation of mononuclear cell proliferation
5	GO:0050806 Positive regulation of synaptic transmission

5	GO:0097553 Calcium ion transmembrane import into cytosol
4	GO:0006096 Glycolytic process
5	GO:0008361 Regulation of cell size
5	GO:0006006 Glucose metabolic process
5	GO:1901292 Nucleoside phosphate catabolic process
6	GO:0032944 Regulation of mononuclear cell proliferation
3	GO:0048873 Homeostasis of number of cells within a tissue
3	GO:0071402 Cellular response to lipoprotein particle stimulus
3	GO:1904994 Regulation of leukocyte adhesion to vascular endothelial cell
5	GO:0046777 Protein autophosphorylation
10	GO:0040007 Growth
4	GO:0046032 ADP catabolic process
6	GO:0048511 Rhythmic process
8	GO:0019221 Cytokine-mediated signalling pathway
15	GO:0071705 Nitrogen compound transport
4	GO:0010469 Regulation of signalling receptor activity
4	GO:0048708 Astrocyte differentiation
7	GO:0002768 Immune response-regulating cell surface receptor signalling pathway
2	GO:0060745 Mammary gland branching involved in pregnancy
4	GO:0019364 Pyridine nucleotide catabolic process
25	GO:0141187 Nucleic acid biosynthetic process
5	GO:0002708 Positive regulation of lymphocyte mediated immunity
4	GO:0009137 Purine nucleoside diphosphate catabolic process
4	GO:0009181 Purine ribonucleoside diphosphate catabolic process
5	GO:0009266 Response to temperature stimulus
3	GO:0045981 Positive regulation of nucleotide metabolic process
3	GO:1900544 Positive regulation of purine nucleotide metabolic process
4	GO:2000106 Regulation of leukocyte apoptotic process
6	GO:0051054 Positive regulation of DNA metabolic process
5	GO:0060759 Regulation of response to cytokine stimulus
4	GO:0001912 Positive regulation of leukocyte mediated cytotoxicity
4	GO:0043535 Regulation of blood vessel endothelial cell migration
4	GO:0046330 Positive regulation of JNK cascade
12	GO:0045892 Negative regulation of DNA-templated transcription
3	GO:2000108 Positive regulation of leukocyte apoptotic process
14	GO:0045184 Establishment of protein localization
4	GO:0009191 Ribonucleoside diphosphate catabolic process
6	GO:0043491 Phosphatidylinositol 3-kinase/protein kinase b signal transduction
6	GO:0070371 Erk1 and erk2 cascade
4	GO:0072526 Pyridine-containing compound catabolic process
8	GO:0072521 Purine-containing compound metabolic process
4	GO:0046031 Adp metabolic process
3	GO:1902894 Negative regulation of mirna transcription
8	GO:0009611 Response to wounding

4	GO:0042100 B cell proliferation
10	GO:0000122 Negative regulation of transcription by rna polymerase ii
12	GO:1902679 Negative regulation of rna biosynthetic process
8	GO:0051604 Protein maturation
5	GO:0001659 Temperature homeostasis
2	GO:0032071 Regulation of endodeoxyribonuclease activity
2	GO:0070427 Nucleotide-binding oligomerization domain containing 1 signalling pathway
2	GO:0070945 Neutrophil-mediated killing of gram-negative bacterium
5	GO:0070665 Positive regulation of leukocyte proliferation
4	GO:0009134 Nucleoside diphosphate catabolic process
7	GO:0006163 Purine nucleotide metabolic process
7	GO:0051960 Regulation of nervous system development
3	GO:0033280 Response to vitamin d
3	GO:2000629 Negative regulation of mirna metabolic process
5	GO:0016051 Carbohydrate biosynthetic process
5	GO:0032869 Cellular response to insulin stimulus
6	GO:0070663 Regulation of leukocyte proliferation
11	GO:0044248 Cellular catabolic process
4	GO:0014013 Regulation of gliogenesis
7	GO:0022411 Cellular component disassembly
7	GO:1903706 Regulation of hemopoiesis
3	GO:0010573 Vascular endothelial growth factor production
4	GO:0031343 Positive regulation of cell killing
4	GO:0051099 Positive regulation of binding
5	GO:0007623 Circadian rhythm
6	GO:0002758 Innate immune response-activating signalling pathway
7	GO:0015711 Organic anion transport
4	GO:0032602 Chemokine production
4	GO:0032642 Regulation of chemokine production
8	GO:0007169 Cell surface receptor protein tyrosine kinase signalling pathway
5	GO:0002705 Positive regulation of leukocyte mediated immunity
3	GO:0014911 Positive regulation of smooth muscle cell migration
3	GO:0045687 Positive regulation of glial cell differentiation
5	GO:0048469 Cell maturation
2	GO:0002246 Wound healing involved in inflammatory response
2	GO:0019371 Cyclooxygenase pathway
6	GO:0046394 Carboxylic acid biosynthetic process
3	GO:0002369 T cell cytokine production
3	GO:0002724 Regulation of t cell cytokine production
3	GO:0010934 Macrophage cytokine production
3	GO:0010935 Regulation of macrophage cytokine production
10	GO:0000902 Cell morphogenesis
7	GO:0031329 Regulation of cellular catabolic process
6	GO:0016053 Organic acid biosynthetic process

4	GO:0009135 Purine nucleoside diphosphate metabolic process
4	GO:0009179 Purine ribonucleoside diphosphate metabolic process
8	GO:0120035 Regulation of plasma membrane bounded cell projection organization
9	GO:0060322 Head development
3	GO:0030890 Positive regulation of b cell proliferation
5	GO:0007626 Locomotory behaviour
4	GO:2001056 Positive regulation of cysteine-type endopeptidase activity
3	GO:0060443 Mammary gland morphogenesis
6	GO:0002263 Cell activation involved in immune response
6	GO:0006913 Nucleocytoplasmic transport
6	GO:0051169 Nuclear transport
6	GO:0070588 Calcium ion transmembrane transport
4	GO:0002526 Acute inflammatory response
6	GO:0040013 Negative regulation of locomotion
3	GO:0006111 Regulation of gluconeogenesis
3	GO:0033028 Myeloid cell apoptotic process
8	GO:0048589 Developmental growth
3	GO:1990776 Response to angiotensin
6	GO:0030198 Extracellular matrix organization
4	GO:0008286 Insulin receptor signalling pathway
2	GO:0031392 Regulation of prostaglandin biosynthetic process
2	GO:0032070 Regulation of deoxyribonuclease activity
2	GO:0035701 Hematopoietic stem cell migration
6	GO:0043062 Extracellular structure organization
6	GO:0050890 Cognition
2	GO:0070391 Response to lipoteichoic acid
2	GO:0071223 Cellular response to lipoteichoic acid
2	GO:1901984 Negative regulation of protein acetylation
2	GO:1904179 Positive regulation of adipose tissue development
8	GO:0031344 Regulation of cell projection organization
6	GO:0045229 External encapsulating structure organization
6	GO:0051235 Maintenance of location
3	GO:0030195 Negative regulation of blood coagulation
3	GO:0032309 Icosanoid secretion
4	GO:0071482 Cellular response to light stimulus
7	GO:0006091 Generation of precursor metabolites and energy
5	GO:0019318 Hexose metabolic process
17	GO:0008104 Protein localization
4	GO:0032755 Positive regulation of interleukin-6 production
3	GO:0090279 Regulation of calcium ion import
3	GO:1900047 Negative regulation of hemostasis
6	GO:0008202 Steroid metabolic process
4	GO:0006090 Pyruvate metabolic process
4	GO:0072676 Lymphocyte migration

5	GO:0051651 Maintenance of location in cell
6	GO:0060326 Cell chemotaxis
17	GO:0070727 Cellular macromolecule localization
4	GO:0009185 Ribonucleoside diphosphate metabolic process
4	GO:0042303 Molting cycle
4	GO:0042633 Hair cycle
4	GO:0010821 Regulation of mitochondrion organization
4	GO:0033209 Tumour necrosis factor-mediated signalling pathway
5	GO:0043542 Endothelial cell migration
12	GO:0051253 Negative regulation of RNA metabolic process
9	GO:0061024 Membrane organization
2	GO:0006265 Dna topological change
2	GO:0019065 Receptor-mediated endocytosis of virus by host cell
4	GO:0046683 Response to organophosphorus
3	GO:0014075 Response to amine
9	GO:0022603 Regulation of anatomical structure morphogenesis
5	GO:0071356 Cellular response to tumour necrosis factor
6	GO:0031346 Positive regulation of cell projection organization
4	GO:1990748 Cellular detoxification
6	GO:0031647 Regulation of protein stability
6	GO:0031400 Negative regulation of protein modification process
3	GO:0042771 Intrinsic apoptotic signalling pathway in response to dna damage by p53 class mediator
4	GO:0043534 Blood vessel endothelial cell migration
3	GO:0050819 Negative regulation of coagulation
3	GO:0061756 Leukocyte adhesion to vascular endothelial cell
3	GO:0140353 Lipid export from cell
6	GO:0032535 Regulation of cellular component size
6	GO:1902105 Regulation of leukocyte differentiation
5	GO:0031331 Positive regulation of cellular catabolic process
7	GO:0050804 Modulation of chemical synaptic transmission
7	GO:0008015 Blood circulation
6	GO:0019058 Viral life cycle
3	GO:0031648 Protein destabilization
3	GO:0031670 Cellular response to nutrient
3	GO:0032092 Positive regulation of protein binding
3	GO:0043368 Positive t cell selection
3	GO:0045332 Phospholipid translocation
7	GO:0099177 Regulation of trans-synaptic signalling
4	GO:1901222 Regulation of non-canonical NF-Kappab signal transduction
5	GO:0046631 Alpha-beta t cell activation
4	GO:0009132 Nucleoside diphosphate metabolic process
6	GO:0042742 Defence response to bacterium
7	GO:0009117 Nucleotide metabolic process
3	GO:0042743 Hydrogen peroxide metabolic process

3	GO:0043536 Positive regulation of blood vessel endothelial cell migration
2	GO:0008291 Acetylcholine metabolic process
2	GO:1900619 Acetate ester metabolic process
2	GO:1990535 Neuron projection maintenance
2	GO:2001279 Regulation of unsaturated fatty acid biosynthetic process
3	GO:1904645 Response to amyloid-beta
4	GO:0001101 Response to acid chemical
5	GO:0043523 Regulation of neuron apoptotic process
4	GO:0032388 Positive regulation of intracellular transport
13	GO:0007049 Cell cycle
4	GO:0043467 Regulation of generation of precursor metabolites and energy
4	GO:0045834 Positive regulation of lipid metabolic process
6	GO:0050767 Regulation of neurogenesis
4	GO:0097237 Cellular response to toxic substance
3	GO:0060612 Adipose tissue development
3	GO:0070542 Response to fatty acid
4	GO:0045598 Regulation of fat cell differentiation
5	GO:0005996 Monosaccharide metabolic process
2	GO:0045793 Positive regulation of cell size
2	GO:0075509 Endocytosis involved in viral entry into host cell
2	GO:0097067 Cellular response to thyroid hormone stimulus
2	GO:1903054 Negative regulation of extracellular matrix organization
2	GO:1903238 Positive regulation of leukocyte tethering or rolling
4	GO:0010950 Positive regulation of endopeptidase activity
5	GO:0045444 Fat cell differentiation
3	GO:0034204 Lipid translocation
3	GO:1990000 Amyloid fibril formation
3	GO:2001239 Regulation of extrinsic apoptotic signalling pathway in absence of ligand
5	GO:1903320 Regulation of protein modification by small protein conjugation or removal
5	GO:0016485 Protein processing
3	GO:0010543 Regulation of platelet activation
3	GO:0051341 Regulation of oxidoreductase activity
7	GO:0080135 Regulation of cellular response to stress
3	GO:1904705 Regulation of vascular associated smooth muscle cell proliferation
4	GO:0017015 Regulation of transforming growth factor beta receptor signalling pathway
5	GO:0031669 Cellular response to nutrient levels
5	GO:0002685 Regulation of leukocyte migration
3	GO:0032735 Positive regulation of interleukin-12 production
14	GO:0043933 Protein-containing complex organization
3	GO:0070228 Regulation of lymphocyte apoptotic process
5	GO:1901873 Regulation of post-translational protein modification
4	GO:0046328 Regulation of jnk cascade
2	GO:0051918 Negative regulation of fibrinolysis
2	GO:0070944 Neutrophil-mediated killing of bacterium

2	GO:0071888 Macrophage apoptotic process
3	GO:0070231 T cell apoptotic process
3	GO:1990874 Vascular associated smooth muscle cell proliferation
5	GO:0009636 Response to toxic substance
4	GO:0007259 Cell surface receptor signalling pathway via JAK-stat
4	GO:1903844 Regulation of cellular response to transforming growth factor beta stimulus
6	GO:0019693 Ribose phosphate metabolic process
6	GO:0050878 Regulation of body fluid levels
7	GO:0001525 Angiogenesis
3	GO:0031663 Lipopolysaccharide-mediated signalling pathway
3	GO:0046686 Response to cadmium ion
5	GO:0071560 Cellular response to transforming growth factor beta stimulus
8	GO:0001568 Blood vessel development
7	GO:0034655 Nucleobase-containing compound catabolic process
3	GO:0002931 Response to ischemia
9	GO:0071345 Cellular response to cytokine stimulus
5	GO:0032868 Response to insulin
5	GO:0032386 Regulation of intracellular transport
4	GO:0010952 Positive regulation of peptidase activity
2	GO:0032308 Positive regulation of prostaglandin secretion
2	GO:0045348 Positive regulation of MHC class ii biosynthetic process
2	GO:0060766 Negative regulation of androgen receptor signalling pathway
2	GO:0070943 Neutrophil-mediated killing of symbiont cell
2	GO:1900120 Regulation of receptor binding
2	GO:1904177 Regulation of adipose tissue development
3	GO:0097035 Regulation of membrane lipid distribution
5	GO:0001894 Tissue homeostasis
5	GO:0060249 Anatomical structure homeostasis
3	GO:0046626 Regulation of insulin receptor signalling pathway
5	GO:0071559 Response to transforming growth factor beta
12	GO:0120036 Plasma membrane bounded cell projection organization
3	GO:0030888 Regulation of b cell proliferation
12	GO:0045934 Negative regulation of nucleobase-containing compound metabolic process
4	GO:0098754 Detoxification
23	GO:0032774 Rna biosynthetic process
5	GO:0006898 Receptor-mediated endocytosis
5	GO:0090092 Regulation of transmembrane receptor protein serine/threonine kinase signaling pathway
5	GO:0097305 Response to alcohol
7	GO:1901615 Organic hydroxy compound metabolic process
3	GO:0015909 Long-chain fatty acid transport
3	GO:0030193 Regulation of blood coagulation
2	GO:0032306 Regulation of prostaglandin secretion
2	GO:0043518 Negative regulation of dna damage response signal transduction by p53 class mediator

2	GO:0060546 Negative regulation of necroptotic process
2	GO:0071316 Cellular response to nicotine
3	GO:0071715 Icosanoid transport
2	GO:2000321 Positive regulation of t-helper 17 cell differentiation
4	GO:0007612 Learning
7	GO:0005975 Carbohydrate metabolic process
4	GO:0046634 Regulation of alpha-beta t cell activation
5	GO:0003018 Vascular process in circulatory system
3	GO:0001774 Microglial cell activation
8	GO:0007420 Brain development
5	GO:0071695 Anatomical structure maturation
8	GO:0001944 Vasculature development
4	GO:0120161 Regulation of cold-induced thermogenesis
3	GO:0006473 Protein acetylation
3	GO:0032623 Interleukin-2 production
3	GO:0032663 Regulation of interleukin-2 production
3	GO:0050850 Positive regulation of calcium-mediated signalling
3	GO:0051289 Protein homotetramerization
3	GO:1900046 Regulation of hemostasis
4	GO:0001910 Regulation of leukocyte mediated cytotoxicity
4	GO:0018205 Peptidyl-lysine modification
4	GO:0030183 B cell differentiation
4	GO:0031960 Response to corticosteroid
6	GO:0030900 Forebrain development
2	GO:0000712 Resolution of meiotic recombination intermediates
3	GO:0014910 Regulation of smooth muscle cell migration
2	GO:0035729 Cellular response to hepatocyte growth factor stimulus
2	GO:0070234 Positive regulation of t cell apoptotic process
2	GO:0070942 Neutrophil mediated cytotoxicity
2	GO:0072224 Metanephric glomerulus development
2	GO:1902166 Negative regulation of intrinsic apoptotic signalling pathway in response to dna damage by p53 class mediator
2	GO:2000343 Positive regulation of chemokine (C-X-C motif) ligand 2 production
4	GO:0106106 Cold-induced thermogenesis
3	GO:0002269 Leukocyte activation involved in inflammatory response
12	GO:0030030 Cell projection organization
3	GO:0061180 Mammary gland epithelium development
3	GO:0071260 Cellular response to mechanical stimulus
4	GO:0050728 Negative regulation of inflammatory response
4	GO:1903169 Regulation of calcium ion transmembrane transport
2	GO:0010544 Negative regulation of platelet activation
2	GO:0050872 White fat cell differentiation
2	GO:0062099 Negative regulation of programmed necrotic cell death
3	GO:0071496 Cellular response to external stimulus
2	GO:1903236 Regulation of leukocyte tethering or rolling

6	GO:0001503 Ossification
6	GO:0050673 Epithelial cell proliferation
9	GO:0098660 Inorganic ion transmembrane transport
4	GO:0006606 Protein import into nucleus
3	GO:0014015 Positive regulation of gliogenesis
3	GO:0050818 Regulation of coagulation
6	GO:0043434 Response to peptide hormone
4	GO:0050852 T cell receptor signalling pathway
2	GO:0035728 Response to hepatocyte growth factor
2	GO:0045346 Regulation of MHC class ii biosynthetic process
2	GO:0050862 Positive regulation of t cell receptor signalling pathway
2	GO:0051917 Regulation of fibrinolysis
2	GO:1902165 Regulation of intrinsic apoptotic signalling pathway in response to dna damage by p53 class mediator
4	GO:0045807 Positive regulation of endocytosis
4	GO:0071897 DNA biosynthetic process
10	GO:0033043 Regulation of organelle organization
4	GO:0051170 Import into nucleus
7	GO:0002683 Negative regulation of immune system process
3	GO:0061900 Glial cell activation
3	GO:1904427 Positive regulation of calcium ion transmembrane transport
5	GO:0040029 Epigenetic regulation of gene expression
5	GO:0002221 Pattern recognition receptor signalling pathway
4	GO:0120254 Olefinic compound metabolic process
4	GO:0031341 Regulation of cell killing
2	GO:0045342 MHC class ii biosynthetic process
2	GO:0055070 Copper ion homeostasis
2	GO:0070230 Positive regulation of lymphocyte apoptotic process
2	GO:0071498 Cellular response to fluid shear stress
2	GO:0098543 Detection of other organism
2	GO:1900034 Regulation of cellular response to heat
4	GO:0010594 Regulation of endothelial cell migration
3	GO:0061045 Negative regulation of wound healing
9	GO:0048609 Multicellular organismal reproductive process
5	GO:0045765 Regulation of angiogenesis
4	GO:0032635 Interleukin-6 production
4	GO:0032675 Regulation of interleukin-6 production
3	GO:0055013 Cardiac muscle cell development
4	GO:1990845 Adaptive thermogenesis
6	GO:0042391 Regulation of membrane potential
3	GO:0010660 Regulation of muscle cell apoptotic process
3	GO:0014909 Smooth muscle cell migration
3	GO:0019233 Sensory perception of pain
6	GO:0042060 Wound healing
5	GO:1901342 Regulation of vasculature development

2	GO:0032305 Positive regulation of icosanoid secretion
2	GO:0045091 Regulation of single stranded viral RNA replication via double stranded dna intermediate
4	GO:0001959 Regulation of cytokine-mediated signalling pathway
3	GO:0097192 Extrinsic apoptotic signalling pathway in absence of ligand
4	GO:0007009 Plasma membrane organization
4	GO:0046718 Symbiont entry into host cell
4	GO:0016052 Carbohydrate catabolic process
4	GO:0046890 Regulation of lipid biosynthetic process
7	GO:0048514 Blood vessel morphogenesis
3	GO:0009620 Response to fungus
3	GO:0038034 Signal transduction in absence of ligand
2	GO:0043249 Erythrocyte maturation
2	GO:1903978 Regulation of microglial cell activation
3	GO:0032615 Interleukin-12 production
3	GO:0032655 Regulation of interleukin-12 production
3	GO:0046425 Regulation of receptor signalling pathway via jak-stat
9	GO:0006259 Dna metabolic process
5	GO:0071375 Cellular response to peptide hormone stimulus
5	GO:0090287 Regulation of cellular response to growth factor stimulus
4	GO:0044409 Symbiont entry into host
3	GO:0006094 Gluconeogenesis
3	GO:0055006 Cardiac cell development
4	GO:0032409 Regulation of transporter activity
4	GO:0071478 Cellular response to radiation
6	GO:0043603 Amide metabolic process
3	GO:0001960 Negative regulation of cytokine-mediated signalling pathway
3	GO:0032722 Positive regulation of chemokine production
3	GO:0070509 Calcium ion import
3	GO:0072678 T cell migration
2	GO:0032069 Regulation of nuclease activity
2	GO:0032303 Regulation of icosanoid secretion
2	GO:0035235 Ionotropic glutamate receptor signalling pathway
2	GO:0039692 Single stranded viral RNA replication via double stranded DNA intermediate
2	GO:0060444 Branching involved in mammary gland duct morphogenesis
4	GO:0045664 Regulation of neuron differentiation
3	GO:0032481 Positive regulation of type i interferon production
3	GO:0050871 Positive regulation of B cell activation
4	GO:0071248 Cellular response to metal ion
3	GO:0019319 Hexose biosynthetic process
3	GO:0048145 Regulation of fibroblast proliferation
3	GO:0072332 Intrinsic apoptotic signaling pathway by p53 class mediator
2	GO:0072567 Chemokine (C-X-C motif) ligand 2 production

Table S2. GO enrichment cellular components.

Number of genes	Pathway
20	GO:0009986 Cell surface
23	GO:0043230 Extracellular organelle
23	GO:0065010 Extracellular membrane-bounded organelle
23	GO:0070062 Extracellular exosome
23	GO:1903561 Extracellular vesicle
11	GO:0043235 Receptor complex
31	GO:0031982 Vesicle
11	GO:0009897 External side of plasma membrane
13	GO:0048471 Perinuclear region of cytoplasm
13	GO:0098552 Side of membrane
25	GO:0005615 Extracellular space
10	GO:0043025 Neuronal cell body
7	GO:0005775 Vacuolar lumen
9	GO:0062023 Collagen-containing extracellular matrix
12	GO:0036477 Somatodendritic compartment
10	GO:0044297 Cell body
4	GO:0031904 Endosome lumen
21	GO:0031410 Cytoplasmic vesicle
8	GO:0034774 Secretory granule lumen
21	GO:0097708 Intracellular vesicle
19	GO:0120025 Plasma membrane bounded cell projection
8	GO:0031983 Vesicle lumen
8	GO:0060205 Cytoplasmic vesicle lumen
12	GO:0030141 Secretory granule
14	GO:0043005 Neuron projection
19	GO:0042995 Cell projection
13	GO:0099503 Secretory vesicle
11	GO:0000323 Lytic vacuole
11	GO:0005764 Lysosome
9	GO:0030312 External encapsulating structure
9	GO:0031012 Extracellular matrix
7	GO:0045121 Membrane raft
7	GO:0098857 Membrane microdomain
8	GO:0045177 Apical part of cell
13	GO:0098590 Plasma membrane region
11	GO:0005773 Vacuole
25	GO:0005576 Extracellular region
3	GO:0036019 Endolysosome
2	GO:0036021 Endolysosome lumen

7	GO:0016324 Apical plasma membrane
2	GO:0032059 Bleb
4	GO:0043202 Lysosomal lumen
8	GO:0030425 Dendrite
8	GO:0097447 Dendritic tree
11	GO:0005768 Endosome
7	GO:0005667 Transcription regulator complex
4	GO:1904813 Ficolin-1-rich granule lumen
4	GO:0098862 Cluster of actin-based cell projections
3	GO:0031594 Neuromuscular junction
3	GO:0000792 Heterochromatin
22	GO:0005654 Nucleoplasm
5	GO:0005788 Endoplasmic reticulum lumen
15	GO:0030054 Cell junction
3	GO:0005901 Caveola
7	GO:0030424 Axon
2	GO:0071682 Endocytic vesicle lumen
3	GO:0035578 Azurophil granule lumen
7	GO:0098797 Plasma membrane protein complex
4	GO:0101002 Ficolin-1-rich granule
5	GO:0031253 Cell projection membrane
3	GO:0005903 Brush border
8	GO:0070161 Anchoring junction
12	GO:0005739 Mitochondrion
3	GO:0044853 Plasma membrane raft
11	GO:0045202 Synapse
5	GO:0031252 Cell leading edge
3	GO:0072562 Blood microparticle
5	GO:0030139 Endocytic vesicle
12	GO:0005694 Chromosome
2	GO:0043209 Myelin sheath
8	GO:0005730 Nucleolus
5	GO:0005925 Focal adhesion
1	GO:0030485 Smooth muscle contractile fiber
19	GO:0031090 Organelle membrane
1	GO:0031379 RNA-directed RNA polymerase complex
1	GO:0061773 Enosc complex
3	GO:0043204 Perikaryon
5	GO:0030055 Cell-substrate junction
3	GO:0030134 Copii-coated er to golgi transport vesicle
3	GO:0001650 Fibrillar center
3	GO:0005766 Primary lysosome
3	GO:0042582 Azurophil granule
1	GO:0005899 Insulin receptor complex

1	GO:0031905 Early endosome lumen
1	GO:1902937 Inward rectifier potassium channel complex
2	GO:0031526 Brush border membrane
6	GO:0010008 Endosome membrane
2	GO:0005637 Nuclear inner membrane
9	GO:0000785 Chromatin
2	GO:0000791 Euchromatin
12	GO:0005783 Endoplasmic reticulum
3	GO:0031256 Leading edge membrane
1	GO:0032449 CBM complex
1	GO:0033553 rDNA heterochromatin
1	GO:0046696 Lipopolysaccharide receptor complex
1	GO:0071159 NF-Kappab complex
2	GO:1904724 Tertiary granule lumen
2	GO:0005771 Multivesicular body
2	GO:0031093 Platelet alpha granule lumen
1	GO:0097226 Sperm mitochondrial sheath
1	GO:0097342 Ripoptosome
1	GO:0098591 External side of apical plasma membrane
3	GO:0098802 Plasma membrane signalling receptor complex
2	GO:0017053 Transcription repressor complex
1	GO:0030056 Hemidesmosome
1	GO:0046581 Intercellular canaliculus
4	GO:0005770 Late endosome
2	GO:0035580 Specific granule lumen
2	GO:0000118 Histone deacetylase complex
1	GO:0017146 NMDA selective glutamate receptor complex
1	GO:0031264 Death-inducing signalling complex
1	GO:0044294 Dendritic growth cone
1	GO:1990712 HFE-transferrin receptor complex
2	GO:0008076 Voltage-gated potassium channel complex
2	GO:0031091 Platelet alpha granule
2	GO:0034705 Potassium channel complex
1	GO:1904090 Peptidase inhibitor complex
6	GO:0005815 Microtubule organizing center
8	GO:0098796 Membrane protein complex
3	GO:0016323 Basolateral plasma membrane
1	GO:0000782 Telomere cap complex
1	GO:0000783 Nuclear telomere cap complex
1	GO:0005677 Chromatin silencing complex
8	GO:0030659 Cytoplasmic vesicle membrane
1	GO:0035253 Ciliary rootlet
1	GO:0044292 Dendrite terminus
8	GO:0012506 Vesicle membrane

3	GO:0150034 Distal axon
1	GO:0042583 Chromaffin granule
1	GO:0140445 Chromosome telomeric repeat region
2	GO:0005796 Golgi lumen
1	GO:0016581 Nurd complex
2	GO:0016605 Pml body
1	GO:0035098 Esc/e(z) complex
2	GO:0042470 Melanosome
2	GO:0048770 Pigment granule
1	GO:0090545 Chd-type complex
3	GO:0009925 Basal plasma membrane
4	GO:0005769 Early endosome
1	GO:0043034 Costamere
1	GO:0097524 Sperm plasma membrane
1	GO:0005641 Nuclear envelope lumen
4	GO:0005911 Cell-cell junction
5	GO:0005813 Centrosome
3	GO:0045178 Basal part of cell
4	GO:0005635 Nuclear envelope
1	GO:0031258 Lamellipodium membrane
11	GO:0098588 Bounding membrane of organelle
1	GO:0005697 Telomerase holoenzyme complex
1	GO:0070822 Sin3-type complex
7	GO:0005789 Endoplasmic reticulum membrane
1	GO:0042824 Mhc class i peptide loading complex
1	GO:0043083 Synaptic cleft
4	GO:0098978 Glutamatergic synapse
7	GO:0031967 Organelle envelope
7	GO:0098827 Endoplasmic reticulum subcompartment
7	GO:0140513 Nuclear protein-containing complex
2	GO:0009898 Cytoplasmic side of plasma membrane
2	GO:0030426 Growth cone
7	GO:0042175 Nuclear outer membrane-endoplasmic reticulum membrane network
1	GO:0042612 Mhc class i protein complex
1	GO:0044295 Axonal growth cone
4	GO:0019866 Organelle inner membrane
2	GO:0030427 Site of polarized growth
8	GO:0031984 Organelle subcompartment
1	GO:0032809 Neuronal cell body membrane
3	GO:0034702 Monoatomic ion channel complex
2	GO:0043197 Dendritic spine
1	GO:0044298 Cell body membrane
2	GO:0044309 Neuron spine
2	GO:0000781 Chromosome telomeric region

2	GO:0001726 Ruffle
7	GO:0015630 Microtubule cytoskeleton
3	GO:0030135 Coated vesicle
2	GO:0042734 Presynaptic membrane
4	GO:0098793 Presynapse
1	GO:0005640 Nuclear outer membrane
1	GO:0005790 Smooth endoplasmic reticulum
1	GO:0051233 Spindle midzone
2	GO:0045335 Phagocytic vesicle
1	GO:0001891 Phagocytic cup
8	GO:0005794 Golgi apparatus
2	GO:0070820 Tertiary granule
1	GO:0008328 Ionotropic glutamate receptor complex
1	GO:0032590 Dendrite membrane
2	GO:0042581 Specific granule
3	GO:0098687 Chromosomal region
1	GO:0043198 Dendritic shaft
1	GO:0002102 Podosome
2	GO:0098562 Cytoplasmic side of membrane
1	GO:0098878 Neurotransmitter receptor complex
1	GO:0016235 Aggresome
1	GO:0009295 Nucleoid
1	GO:0042645 Mitochondrial nucleoid
8	GO:1902494 Catalytic complex
2	GO:0034703 Cation channel complex
2	GO:0008021 Synaptic vesicle

Table S3. GO enrichment molecular function.

Number of genes	Pathway
31	GO:0042802 Identical protein binding
26	GO:0019899 Enzyme binding
15	GO:0042803 Protein homodimerization activity
25	GO:0140096 Catalytic activity acting on a protein
16	GO:0046983 Protein dimerization activity
7	GO:0001221 Transcription coregulator binding
5	GO:0001223 Transcription coactivator binding
8	GO:0017171 Serine hydrolase activity
13	GO:0019900 Kinase binding
17	GO:0044877 Protein-containing complex binding
10	GO:0004175 Endopeptidase activity
12	GO:0019901 Protein kinase binding
22	GO:0016787 Hydrolase activity
7	GO:0004252 Serine-type endopeptidase activity

11	GO:0008233 Peptidase activity
7	GO:0008236 Serine-type peptidase activity
10	GO:0008134 Transcription factor binding
28	GO:0043169 Cation binding
27	GO:0046872 Metal ion binding
2	GO:0003918 DNA topoisomerase type ii (double strand cut ATP-hydrolyzing) activity
2	GO:0051425 PTB domain binding
4	GO:0004879 Nuclear receptor activity
4	GO:0098531 Ligand-activated transcription factor activity
3	GO:0004407 Histone deacetylase activity
18	GO:0097367 Carbohydrate derivative binding
4	GO:0004714 Transmembrane receptor protein tyrosine kinase activity
3	GO:0033558 Protein lysine deacetylase activity
5	GO:0016922 Nuclear receptor binding
3	GO:0016702 Oxidoreductase activity acting on single donors with incorporation of molecular oxygen incorporation of two atoms of oxygen
7	GO:0042277 Peptide binding
7	GO:0061629 RNA polymerase ii-specific DNA-binding transcription factor binding
3	GO:0016701 Oxidoreductase activity acting on single donors with incorporation of molecular oxygen
9	GO:0003682 Chromatin binding
5	GO:0004713 Protein tyrosine kinase activity
4	GO:0005518 Collagen binding
9	GO:0019904 Protein domain specific binding
3	GO:0051059 NF-Kappab binding
2	GO:0160008 Protein decrotonylase activity
2	GO:0160009 Histone decrotonylase activity
5	GO:0002020 Protease binding
4	GO:0019199 Transmembrane receptor protein kinase activity
4	GO:0004197 Cysteine-type endopeptidase activity
14	GO:0005102 Signaling receptor binding
15	GO:0038023 Signaling receptor activity
15	GO:0060089 Molecular transducer activity
4	GO:0001540 Amyloid-beta binding
3	GO:0043394 Proteoglycan binding
2	GO:0030235 Nitric-oxide synthase regulator activity
5	GO:0008201 Heparin binding
3	GO:0019213 Deacetylase activity
2	GO:0003916 DNA topoisomerase activity
3	GO:0001046 Core promoter sequence-specific DNA binding
3	GO:0001530 Lipopolysaccharide binding
3	GO:0048156 Tau protein binding
4	GO:0005496 Steroid binding
3	GO:0043028 Cysteine-type endopeptidase regulator activity involved in apoptotic process
12	GO:0030234 Enzyme regulator activity

11	GO:0046914 Transition metal ion binding
2	GO:0008239 Dipeptidyl-peptidase activity
2	GO:0034056 Estrogen response element binding
2	GO:0097677 Stat family protein binding
7	GO:0140297 DNA-binding transcription factor binding
3	GO:0001222 Transcription corepressor binding
9	GO:0008289 Lipid binding
3	GO:0140296 General transcription initiation factor binding
11	GO:0140677 Molecular function activator activity
13	GO:0030554 Adenyl nucleotide binding
4	GO:0042826 Histone deacetylase binding
4	GO:0099094 Ligand-gated monoatomic cation channel activity
2	GO:0043560 Insulin receptor substrate binding
2	GO:0120283 Protein serine/threonine kinase binding
5	GO:0033218 Amide binding
5	GO:0005539 Glycosaminoglycan binding
16	GO:0043168 Anion binding
4	GO:0031490 Chromatin DNA binding
4	GO:0020037 Heme binding
15	GO:0098772 Molecular function regulator activity
2	GO:0046965 Nuclear retinoid x receptor binding
4	GO:0046906 Tetrapyrrole binding
5	GO:1901681 Sulfur compound binding
12	GO:0004888 Transmembrane signaling receptor activity
3	GO:0016811 Hydrolase activity acting on carbon-nitrogen (but not peptide) bonds in linear amides
2	GO:0000979 RNA polymerase ii core promoter sequence-specific DNA binding
3	GO:0002039 P53 binding
2	GO:0030506 Ankyrin binding
7	GO:0008047 Enzyme activator activity
12	GO:0043565 Sequence-specific DNA binding
15	GO:0016740 Transferase activity
2	GO:0043027 Cysteine-type endopeptidase inhibitor activity involved in apoptotic process
4	GO:0015276 Ligand-gated monoatomic ion channel activity
4	GO:0022834 Ligand-gated channel activity
2	GO:0016505 Peptidase activator activity involved in apoptotic process
14	GO:0000166 Nucleotide binding
4	GO:0008234 Cysteine-type peptidase activity
3	GO:0016209 Antioxidant activity
2	GO:0017025 TBP-class protein binding
5	GO:0031625 Ubiquitin protein ligase binding
3	GO:0051213 Dioxygenase activity
14	GO:1901265 Nucleoside phosphate binding
4	GO:0003714 Transcription corepressor activity
2	GO:0042974 Nuclear retinoic acid receptor binding

3	GO:0008238 Exopeptidase activity
5	GO:0044389 Ubiquitin-like protein ligase binding
4	GO:0019902 Phosphatase binding
15	GO:0003677 DNA binding
4	GO:0140993 Histone modifying activity
11	GO:0005524 ATP binding
13	GO:0017076 Purine nucleotide binding
3	GO:0008013 Beta-catenin binding
2	GO:0003707 Nuclear steroid receptor activity
8	GO:0008270 Zinc ion binding
11	GO:0032559 Adenyl ribonucleotide binding
14	GO:1901363 Heterocyclic compound binding
9	GO:0000978 RNA polymerase ii cis-regulatory region sequence-specific dna binding
4	GO:0061134 Peptidase regulator activity
4	GO:0016747 Acyltransferase activity transferring groups other than amino-acyl groups
11	GO:0003690 Double-stranded DNA binding
7	GO:0016491 Oxidoreductase activity
2	GO:0042805 Actinin binding
9	GO:0000987 Cis-regulatory region sequence-specific DNA binding
4	GO:0140097 Catalytic activity acting on DNA
2	GO:0004177 Aminopeptidase activity
2	GO:0042162 Telomeric DNA binding
6	GO:0004672 Protein kinase activity
2	GO:0016799 Hydrolase activity hydrolyzing n-glycosyl compounds
2	GO:0030331 Nuclear estrogen receptor binding
3	GO:0051087 Protein-folding chaperone binding
7	GO:0016746 Acyltransferase activity
10	GO:0000976 Transcription cis-regulatory region binding
10	GO:0001067 Transcription regulatory region nucleic acid binding
3	GO:0019209 Kinase activator activity
1	GO:0004104 Cholinesterase activity
1	GO:0004699 Diacylglycerol-dependent calcium-independent serine/threonine kinase activity
1	GO:0004992 Platelet activating factor receptor activity
1	GO:0005009 Insulin receptor activity
1	GO:0005017 Platelet-derived growth factor receptor activity
1	GO:0008458 Carnitine o-octanoyltransferase activity
1	GO:0015225 Biotin transmembrane transporter activity
3	GO:0016810 Hydrolase activity acting on carbon-nitrogen (but not peptide) bonds
1	GO:0016890 Site-specific endodeoxyribonuclease activity specific for altered base
1	GO:0032129 Histone h3k9 deacetylase activity
1	GO:0032554 Purine deoxyribonucleotide binding
1	GO:0032557 Pyrimidine ribonucleotide binding
1	GO:0042903 Tubulin deacetylase activity
1	GO:0043398 Hlh domain binding

1	GO:0043993 Histone h3k18 acetyltransferase activity
1	GO:0046970 Nad-dependent histone h4k16 deacetylase activity
1	GO:0051021 Gdp-dissociation inhibitor binding
1	GO:0051022 Rho GDP-dissociation inhibitor binding
2	GO:0051879 Hsp90 protein binding
1	GO:0070644 Vitamin d response element binding
1	GO:0106130 Purine phosphoribosyltransferase activity
1	GO:0140431 DNa-(abasic site) binding
1	GO:1902271 D3 vitamins binding
10	GO:1990837 Sequence-specific double-stranded dna binding
3	GO:0019903 Protein phosphatase binding
1	GO:0001093 TFIIB-class transcription factor binding
1	GO:0004095 Carnitine o-palmitoyltransferase activity
1	GO:0004833 Tryptophan 2,3-dioxygenase activity
1	GO:0016286 Small conductance calcium-activated potassium channel activity
2	GO:0016504 Peptidase activator activity
1	GO:0030284 Nuclear estrogen receptor activity
1	GO:0032552 Deoxyribonucleotide binding
1	GO:0033695 Oxidoreductase activity acting on ch or ch2 groups quinone or similar compoun as acceptor
1	GO:0034739 Histone h4k16 deacetylase activity
1	GO:0034875 Caffeine oxidase activity
1	GO:0038085 Vascular endothelial growth factor binding
1	GO:0050692 DNA binding domain binding
1	GO:0070053 Thrombospondin receptor activity
2	GO:0070888 E-box binding
1	GO:0141050 Histone h3k deacetylase activity
1	GO:0141051 Histone h4k deacetylase activity
1	GO:0160185 Phospholipase c activator activity
2	GO:0004601 Peroxidase activity
2	GO:0016684 Oxidoreductase activity acting on peroxide as acceptor
6	GO:0016773 Phosphotransferase activity alcohol group as acceptor
4	GO:0022836 Gated channel activity
9	GO:0000977 RNA polymerase ii transcription regulatory region sequence-specific dna bindin
11	GO:0035639 Purine ribonucleoside triphosphate binding
1	GO:0004167 Dopachrome isomerase activity
1	GO:0004844 Uracil dna n-glycosylase activity
4	GO:0005261 Monoatomic cation channel activity
1	GO:0016416 O-palmitoyltransferase activity
3	GO:0016705 Oxidoreductase activity acting on paired donors with incorporation or reduction of molecular oxygen
1	GO:0019103 Pyrimidine nucleotide binding
1	GO:0030882 Lipid antigen binding
1	GO:0030883 Endogenous lipid antigen binding
1	GO:0030884 Exogenous lipid antigen binding

1	GO:0043559 Insulin binding
1	GO:0097506 Deaminated base DNA n-glycosylase activity
1	GO:0101021 Estrogen 2-hydroxylase activity
5	GO:0003712 Transcription coregulator activity
7	GO:0016772 Transferase activity transferring phosphorus-containing groups
11	GO:0032555 Purine ribonucleotide binding
3	GO:0003823 Antigen binding
2	GO:0097110 Scaffold protein binding
3	GO:0016853 Isomerase activity
1	GO:0001875 Lipopolysaccharide immune receptor activity
1	GO:0016406 Carnitine o-acyltransferase activity
1	GO:0017136 NAD-dependent histone deacetylase activity
1	GO:0030298 Receptor signaling protein tyrosine kinase activator activity
11	GO:0032553 Ribonucleotide binding
1	GO:0050178 Phenylpyruvate tautomerase activity
1	GO:0071532 Ankyrin repeat binding
1	GO:1990239 Steroid hormone binding
2	GO:1990841 Promoter-specific chromatin binding
7	GO:0008092 Cytoskeletal protein binding
2	GO:0005230 Extracellular ligand-gated monoatomic ion channel activity
2	GO:0008374 O-acyltransferase activity
2	GO:0003684 Damaged DNA binding
1	GO:0003720 Telomerase activity
1	GO:0004528 Phosphodiesterase i activity
1	GO:0004791 Thioredoxin-disulfide reductase (NADPH) activity
1	GO:0004931 Extracellularly ATP-gated monoatomic cation channel activity
1	GO:0004972 NMDA glutamate receptor activity
1	GO:0005499 Vitamin d binding
1	GO:0008311 Double-stranded DNA 3-prime-5-prime DNA exonuclease activity
1	GO:0030911 TPR domain binding
1	GO:0032217 Riboflavin transmembrane transporter activity
2	GO:0033293 Monocarboxylic acid binding
1	GO:0050543 Icosatetraenoic acid binding
1	GO:0050544 Arachidonate binding
1	GO:0050693 LBD domain binding
1	GO:0106256 Hydroperoxy icosatetraenoate dehydratase activity
1	GO:1990254 Keratin filament binding
1	GO:1990405 Protein antigen binding
6	GO:0016301 Kinase activity
1	GO:0004301 Epoxide hydrolase activity
1	GO:0031995 Insulin-like growth factor ii binding
1	GO:0034979 NAD-dependent protein lysine deacetylase activity
1	GO:0050542 Icosanoid binding
1	GO:0101020 Estrogen 16-alpha-hydroxylase activity

18	GO:0003676 Nucleic acid binding
1	GO:0003964 RNA-directed dna polymerase activity
1	GO:0008309 Double-stranded DNA exodeoxyribonuclease activity
1	GO:0015143 Urate transmembrane transporter activity
2	GO:0023026 MHC class ii protein complex binding
1	GO:0035381 ATP-gated ion channel activity
1	GO:0047134 Protein-disulfide reductase (NAD(p)h) activity
1	GO:1901702 Salt transmembrane transporter activity
1	GO:0003691 Double-stranded telomeric DNA binding
1	GO:0004523 RNA-DNA hybrid ribonuclease activity
1	GO:0004955 Prostaglandin receptor activity
2	GO:0008081 Phosphoric diester hydrolase activity
1	GO:0016413 O-acetyltransferase activity
1	GO:0016801 Hydrolase activity acting on ether bonds
1	GO:0016803 Ether hydrolase activity
1	GO:0022849 Glutamate-gated calcium ion channel activity
1	GO:0031727 Ccr2 chemokine receptor binding
1	GO:0071723 Lipopeptide binding
2	GO:0042562 Hormone binding
1	GO:0002162 Dystroglycan binding
1	GO:0004706 Jun kinase kinase kinase activity
1	GO:0004954 Prostanoid receptor activity
1	GO:0032052 Bile acid binding
1	GO:0048407 Platelet-derived growth factor binding
1	GO:0004468 Lysine n-acetyltransferase activity acting on acetyl phosphate as donor
1	GO:0005536 D-glucose binding
2	GO:0016407 Acetyltransferase activity
1	GO:0016668 Oxidoreductase activity acting on a sulfur group of donors NAD(p) as acceptor
1	GO:0016725 Oxidoreductase activity acting on ch or ch2 groups
1	GO:0016862 Intramolecular oxidoreductase activity interconverting keto- and enol-groups
1	GO:0019215 Intermediate filament binding
1	GO:0042166 Acetylcholine binding
1	GO:1902282 Voltage-gated potassium channel activity involved in ventricular cardiac muscle cell action potential repolarization
2	GO:0023023 Mhc protein complex binding
4	GO:0005216 Monoatomic ion channel activity
1	GO:0015269 Calcium-activated potassium channel activity
1	GO:0031994 Insulin-like growth factor i binding
1	GO:0042301 Phosphate ion binding
1	GO:0070513 Death domain binding
2	GO:0043130 Ubiquitin binding
1	GO:0005161 Platelet-derived growth factor receptor binding
1	GO:0031404 Chloride ion binding
1	GO:0086008 Voltage-gated potassium channel activity involved in cardiac muscle cell action potential repolarization
3	GO:0001227 DNA-binding transcription repressor activity rna polymerase ii-specific

10	GO:0140110 Transcription regulator activity
1	GO:0003906 DNA-(apurinic or apyrimidinic site) endonuclease activity
1	GO:0008559 ABC-type xenobiotic transporter activity
3	GO:0001217 DNA-binding transcription repressor activity
4	GO:0001228 DNA-binding transcription activator activity rna polymerase ii-specific
1	GO:0004697 Diacylglycerol-dependent serine/threonine kinase activity
1	GO:0004970 Glutamate-gated receptor activity
1	GO:0005159 Insulin-like growth factor receptor binding
1	GO:0008142 Oxysterol binding
1	GO:0010484 Histone h3 acetyltransferase activity
1	GO:0015562 Efflux transmembrane transporter activity
1	GO:0016888 Endodeoxyribonuclease activity producing 5-prime-phosphomonoesters
1	GO:0019104 DNA n-glycosylase activity
1	GO:0061809 NAD+ nucleotidase cyclic adp-ribose generating
1	GO:0097157 Pre-MRNA intronic binding
4	GO:0001216 DNA-binding transcription activator activity
1	GO:0004953 Icosanoid receptor activity
1	GO:0016805 Dipeptidase activity
3	GO:0019207 Kinase regulator activity
1	GO:0036041 Long-chain fatty acid binding
1	GO:0070403 NAD+ binding
2	GO:0009055 Electron transfer activity
2	GO:0005267 Potassium channel activity
3	GO:0003713 Transcription coactivator activity
1	GO:0008296 3-prime-5-prime-dna exonuclease activity
1	GO:0030881 Beta-2-microglobulin binding
2	GO:0032182 Ubiquitin-like protein binding
1	GO:0005001 Transmembrane receptor protein tyrosine phosphatase activity
2	GO:0008094 ATP-dependent activity acting on DNA
4	GO:0015267 Channel activity
1	GO:0016863 Intramolecular oxidoreductase activity transposing c=c bonds
1	GO:0019198 Transmembrane receptor protein phosphatase activity
4	GO:0022803 Passive transmembrane transporter activity
1	GO:0043395 Heparan sulfate proteoglycan binding
1	GO:0046624 Sphingolipid transporter activity
1	GO:0005520 Insulin-like growth factor binding
1	GO:0008301 DNA binding bending
1	GO:0034185 Apolipoprotein binding
1	GO:0070182 DNA polymerase binding
2	GO:0030295 Protein kinase activator activity
1	GO:0005123 Death receptor binding
3	GO:0005126 Cytokine receptor binding
1	GO:0008656 Cysteine-type endopeptidase activator activity involved in apoptotic process
1	GO:0010485 Histone h4 acetyltransferase activity
2	GO:0019838 Growth factor binding

2	GO:0044325 Transmembrane transporter binding
1	GO:0001614 Purinergic nucleotide receptor activity
1	GO:0016502 Nucleotide receptor activity
2	GO:0016798 Hydrolase activity acting on glycosyl bonds
2	GO:0031072 Heat shock protein binding
1	GO:0016500 Protein-hormone receptor activity
1	GO:0016493 C-c chemokine receptor activity
1	GO:0001091 RNA polymerase ii general transcription initiation factor binding
1	GO:0016004 Phospholipase activator activity
1	GO:0019957 C-c chemokine binding
1	GO:0050699 WW domain binding
1	GO:0004709 Map kinase kinase kinase activity
1	GO:0008066 Glutamate receptor activity
1	GO:0043548 Phosphatidylinositol 3-kinase binding
1	GO:0051787 Misfolded protein binding
1	GO:0070006 Metalloaminopeptidase activity
1	GO:0070034 Telomerase RNA binding
1	GO:0070412 R-SMAD binding
1	GO:0070840 Dynein complex binding
1	GO:0001637 G protein-coupled chemoattractant receptor activity
1	GO:0004950 Chemokine receptor activity
1	GO:0005227 Calcium-activated cation channel activity
1	GO:0016895 DNA exonuclease activity producing 5-prime-phosphomonoesters
1	GO:0030507 Spectrin binding
6	GO:0030674 Protein-macromolecule adaptor activity
1	GO:0004529 DNA exonuclease activity
1	GO:0005242 Inward rectifier potassium channel activity
2	GO:0019955 Cytokine binding
1	GO:0140033 Acetylation-dependent protein binding
2	GO:0015079 Potassium ion transmembrane transporter activity
1	GO:0004435 Phosphatidylinositol phospholipase c activity
1	GO:0005251 Delayed rectifier potassium channel activity
1	GO:0043236 Laminin binding
1	GO:0045499 Chemorepellent activity
1	GO:0051019 Mitogen-activated protein kinase binding
1	GO:0051393 Alpha-actinin binding
1	GO:0060229 Lipase activator activity
4	GO:0048018 Receptor ligand activity
1	GO:0050681 Nuclear androgen receptor binding
1	GO:0070330 Aromatase activity
1	GO:0004181 Metallocarboxypeptidase activity
4	GO:0030546 Signaling receptor activator activity
1	GO:0001968 Fibronectin binding
1	GO:0004629 Phospholipase c activity

1	GO:0008395 Steroid hydroxylase activity
1	GO:0022839 Monoatomic ion-gated channel activity
1	GO:0034061 DNA polymerase activity
1	GO:0043425 BHLH transcription factor binding
1	GO:0099604 Ligand-gated calcium channel activity
1	GO:0042910 Xenobiotic transmembrane transporter activity
1	GO:0097718 Disordered domain specific binding
2	GO:0004866 Endopeptidase inhibitor activity
2	GO:0005244 Voltage-gated monoatomic ion channel activity
2	GO:0005342 Organic acid transmembrane transporter activity
1	GO:0019956 Chemokine binding
2	GO:0031406 Carboxylic acid binding
3	GO:0042578 Phosphoric ester hydrolase activity
2	GO:0046943 Carboxylic acid transmembrane transporter activity
1	GO:0015035 Protein-disulfide reductase activity
1	GO:0030296 Protein tyrosine kinase activator activity
2	GO:0022832 Voltage-gated channel activity
1	GO:0004869 Cysteine-type endopeptidase inhibitor activity
2	GO:0030414 Peptidase inhibitor activity
2	GO:0008237 Metallopeptidase activity
1	GO:0016409 Palmitoyltransferase activity
2	GO:0043177 Organic acid binding
1	GO:0071889 14-3-3 protein binding
4	GO:0140657 ATP-dependent activity
1	GO:0001848 Complement binding
1	GO:0005231 Excitatory extracellular ligand-gated monoatomic ion channel activity
1	GO:0016891 RNA endonuclease activity producing 5-prime-phosphomonoesters
4	GO:0030545 Signaling receptor regulator activity
1	GO:0042887 Amide transmembrane transporter activity
2	GO:0140030 Modification-dependent protein binding
8	GO:0003723 RNA binding
1	GO:0032451 Demethylase activity
1	GO:0038187 Pattern recognition receptor activity
2	GO:0061135 Endopeptidase regulator activity
1	GO:0015036 Disulfide oxidoreductase activity
1	GO:0036002 Pre-mRNA binding
1	GO:0034062 5-prime-3-prime RNA polymerase activity
1	GO:0097747 RNA polymerase activity
1	GO:0004520 DNA endonuclease activity
2	GO:0005516 Calmodulin binding
4	GO:0008324 Monoatomic cation transmembrane transporter activity
1	GO:0048487 Beta-tubulin binding
1	GO:0090482 Vitamin transmembrane transporter activity
4	GO:0140640 Catalytic activity acting on a nucleic acid

1	GO:0008307 Structural constituent of muscle
1	GO:0140662 Atp-dependent protein folding chaperone
1	GO:0043014 Alpha-tubulin binding
1	GO:0004402 Histone acetyltransferase activity
1	GO:0005504 Fatty acid binding
2	GO:0052689 Carboxylic ester hydrolase activity
6	GO:0060090 Molecular adaptor activity
1	GO:0004180 Carboxypeptidase activity
1	GO:0016893 Endonuclease activity active with either ribo- or deoxyribonucleic acids and producing 5-prime-phosphomonoesters
1	GO:0004715 Non-membrane spanning protein tyrosine kinase activity
3	GO:0046873 Metal ion transmembrane transporter activity
1	GO:0016712 Oxidoreductase activity acting on paired donors with incorporation or reduction of molecular oxygen reduced flavin or flavoprotein as one donor and incorporation of one atom of oxygen
2	GO:0000287 Magnesium ion binding
1	GO:0050661 Nadp binding
1	GO:0005080 Protein kinase c binding
1	GO:0017046 Peptide hormone binding
1	GO:1904315 Transmitter-gated monoatomic ion channel activity involved in regulation of postsynaptic membrane potential
5	GO:0004930 G protein-coupled receptor activity
1	GO:0016763 Pentosyltransferase activity
1	GO:0042056 Chemoattractant activity
1	GO:0061733 Peptide-lysine-n-acetyltransferase activity
1	GO:0016667 Oxidoreductase activity acting on a sulfur group of donors
1	GO:0016836 Hydro-lyase activity
1	GO:0051287 NAD binding
1	GO:0099529 Neurotransmitter receptor activity involved in regulation of postsynaptic membrane potential
1	GO:0001098 Basal transcription machinery binding
1	GO:0001099 Basal RNA polymerase ii transcription machinery binding
1	GO:0016597 Amino acid binding
2	GO:0042393 Histone binding
2	GO:0019887 Protein kinase regulator activity
1	GO:0034212 Peptide n-acetyltransferase activity
1	GO:0016860 Intramolecular oxidoreductase activity
1	GO:0050840 Extracellular matrix binding
1	GO:0004536 DNA nuclease activity
1	GO:0008408 3-prime-5-prime exonuclease activity
1	GO:0016796 Exonuclease activity active with either ribo- or deoxyribonucleic acids and producing 5-prime-phosphomonoesters
1	GO:0022824 Transmitter-gated monoatomic ion channel activity
1	GO:0022835 Transmitter-gated channel activity
1	GO:0140359 ABC-type transporter activity
3	GO:0004842 Ubiquitin-protein transferase activity

1	GO:0032934 Sterol binding
1	GO:0008235 Metalloexopeptidase activity
2	GO:0008514 Organic anion transmembrane transporter activity
4	GO:0015075 Monoatomic ion transmembrane transporter activity
1	GO:0030145 Manganese ion binding
1	GO:0031593 Polyubiquitin modification-dependent protein binding
1	GO:0031492 Nucleosomal dna binding
1	GO:0032813 Tumour necrosis factor receptor superfamily binding

Table S4. KEGG pathway enrichment.

Number of genes	Pathway
16	Pathways in cancer
11	NOD-like receptor signalling pathway
7	NF-kappa B signalling pathway
7	Apoptosis
6	TNF signalling pathway
7	Chemical carcinogenesis-receptor activation
6	Fluid shear stress and atherosclerosis
7	Salmonella infection
5	Prostate cancer
6	Transcriptional misregulation in cancer
6	Diabetic cardiomyopathy
6	Lipid and atherosclerosis
4	Platinum drug resistance
5	Necroptosis
4	Antigen processing and presentation
4	Longevity regulating pathway
4	IL-17 signalling pathway
5	Tuberculosis
4	Small cell lung cancer
5	Neutrophil extracellular trap formation
4	C-type lectin receptor signalling pathway
6	MicroRNAs in cancer
4	HIF-1 signalling pathway
4	Toxoplasmosis
6	Human papillomavirus infection
5	Human T-cell leukemia virus 1 infection
5	Chemical carcinogenesis-reactive oxygen species
6	PI3K-Akt signalling pathway
5	Shigellosis
4	Estrogen signalling pathway
3	Regulation of lipolysis in adipocytes
4	Alcoholic liver disease
3	Longevity regulating pathway-multiple species

3	Inflammatory bowel disease
3	Amphetamine addiction
3	Chemical carcinogenesis-DNA adducts
4	Hepatitis B
3	Central carbon metabolism in cancer
3	Leishmaniasis
4	Influenza A
3	Complement and coagulation cascades
3	PD-L1 expression and PD-1 checkpoint pathway in cancer
4	Pathogenic Escherichia coli infection
4	Epstein-Barr virus infection
4	Proteoglycans in cancer
4	Human immunodeficiency virus 1 infection
3	Amoebiasis
5	Neuroactive ligand-receptor interaction
3	Th17 cell differentiation
4	Human cytomegalovirus infection
3	Toll-like receptor signalling pathway
3	Insulin resistance
3	Serotonergic synapse
4	Ras signaling pathway
2	Apoptosis-multiple species
5	Alzheimer disease
4	Coronavirus disease-COVID-19
3	Thyroid hormone signalling pathway
3	T cell receptor signalling pathway
3	AMPK signalling pathway
4	Calcium signalling pathway
2	Thyroid cancer
3	FoxO signalling pathway
3	Lysosome
2	Tryptophan metabolism
3	Phospholipase D signalling pathway
2	Type II diabetes mellitus
2	Cocaine addiction
4	MAPK signalling pathway
3	Efferocytosis
2	Ovarian steroidogenesis
3	Non-alcoholic fatty liver disease
2	Endocrine and other factor-regulated calcium reabsorption
2	Legionellosis
3	Autophagy-animal
2	Notch signalling pathway
2	Arachidonic acid metabolism

3	Herpes simplex virus 1 infection
2	Long-term potentiation
1	Caffeine metabolism
3	Chemokine signalling pathway
3	Kaposi sarcoma-associated herpesvirus infection
2	Prolactin signalling pathway
2	RIG-I-like receptor signalling pathway
2	Non-small cell lung cancer
3	Focal adhesion
2	Metabolism of xenobiotics by cytochrome P450
2	PPAR signalling pathway
3	Viral carcinogenesis
2	Chronic myeloid leukemia
2	Pertussis
3	Rap1 signalling pathway
2	Cytosolic DNA-sensing pathway
2	Polycomb repressive complex
2	B cell receptor signalling pathway
3	cAMP signaling pathway
2	Bile secretion
2	Adherens junction
2	Rheumatoid arthritis
2	Staphylococcus aureus infection
2	Endocrine resistance
2	Fc gamma R-mediated phagocytosis
2	AGE-RAGE signalling pathway in diabetic complications
2	Glycerophospholipid metabolism
2	Protein digestion and absorption
2	Chagas disease
2	Glucagon signalling pathway
2	TGF-beta signalling pathway
2	Cholinergic synapse
2	Parathyroid hormone synthesis secretion and action
4	Pathways of neurodegeneration-multiple diseases
2	Neurotrophin signalling pathway
1	Phenylalanine metabolism
2	Platelet activation
2	Relaxin signalling pathway
3	Huntington disease
8	Metabolic pathways
2	Osteoclast differentiation
2	Yersinia infection
2	Systemic lupus erythematosus
2	Measles

2	Ubiquitin mediated proteolysis
2	Phagosome
2	Cellular senescence
2	Cell cycle
2	Hepatitis C
2	Signalling pathway
2	Hepatocellular carcinoma
2	Protein processing in endoplasmic reticulum
1	Linoleic acid metabolism
1	Antifolate resistance
2	WNT signalling pathway
1	Pentose phosphate pathway
1	Circadian rhythm
2	Alcoholism
1	Tyrosine metabolism
1	African trypanosomiasis
1	Nicotinate and nicotinamide metabolism
1	Aldosterone-regulated sodium reabsorption
1	Primary immunodeficiency
1	Nicotine addiction
1	Bladder cancer
1	Fatty acid degradation
1	Base excision repair
2	Hormone signalling
1	ABC transporters
1	Virion-Hepatitis viruses
2	Regulation of actin cytoskeleton
1	Malaria
2	Thermogenesis
1	Fatty acid metabolism
1	Glutathione metabolism
1	VEGF signalling pathway
1	Mineral absorption

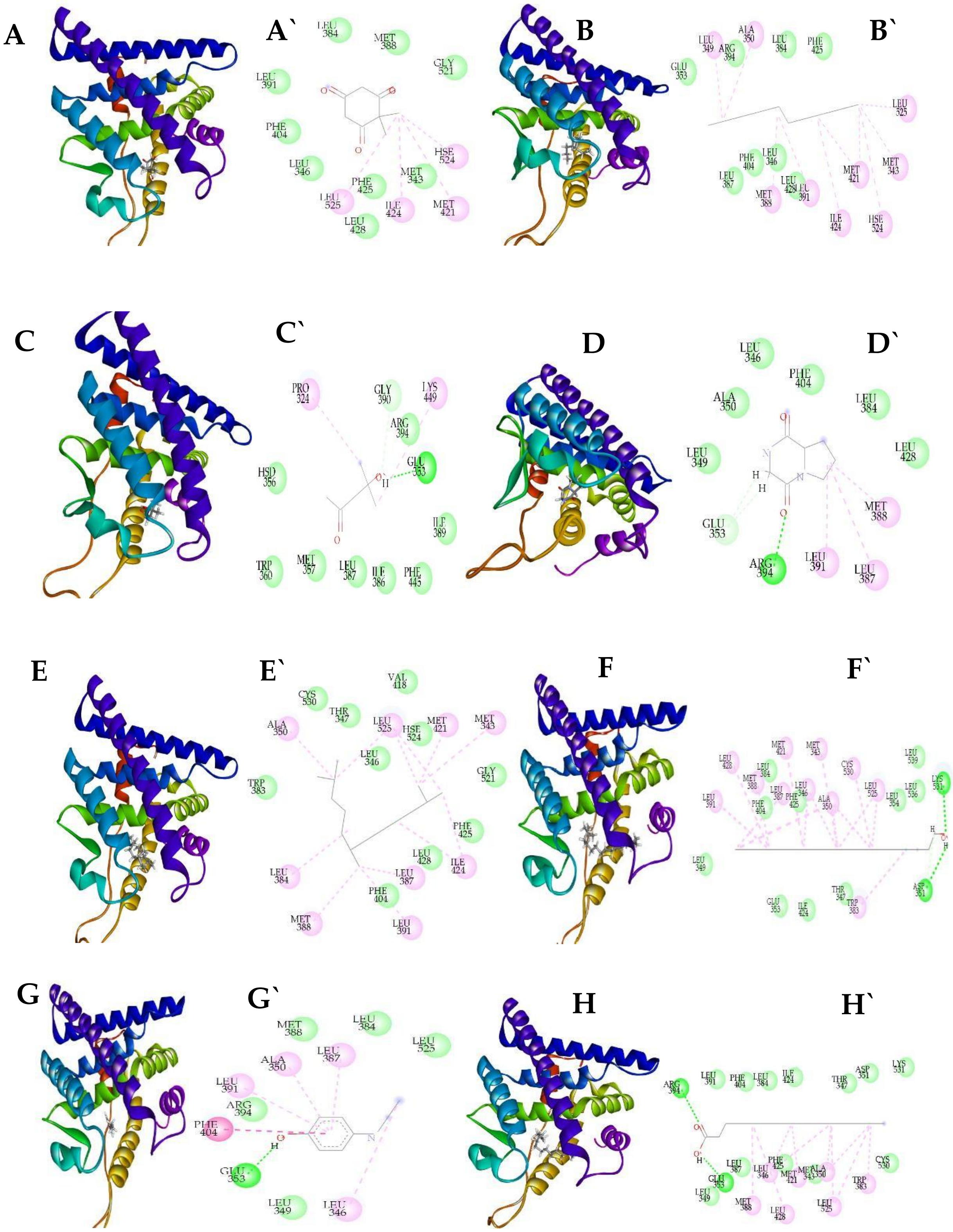

A
A`
B
B`
C
C`
D
D`
E
E`
F
F`
G
G`
H
H`

I

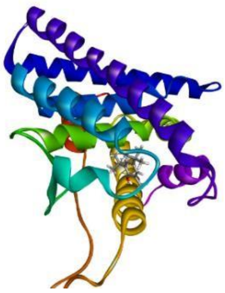

I`

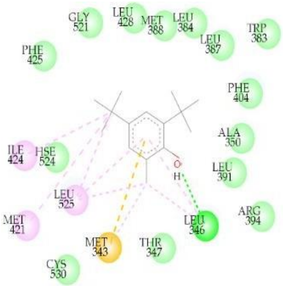

J

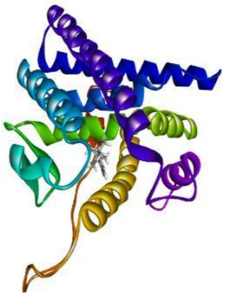

J`

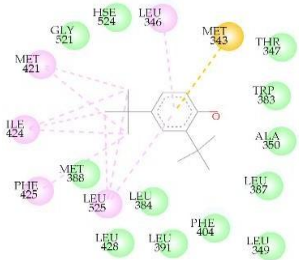

K

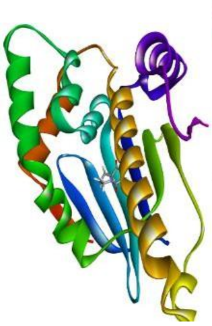

K`

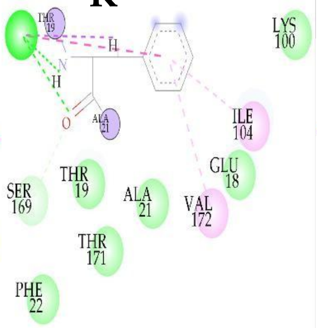

L

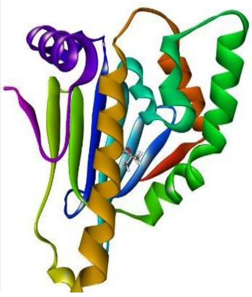

L`

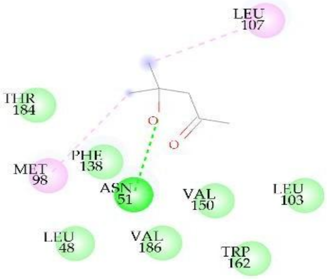

M

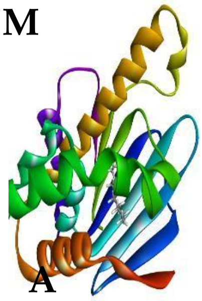

M`

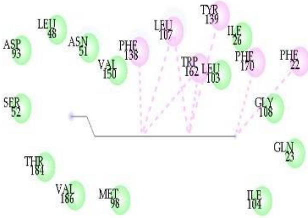

N

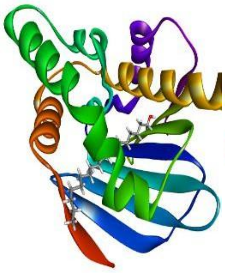

N`

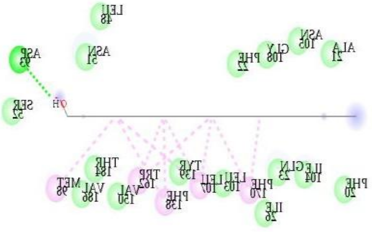

O

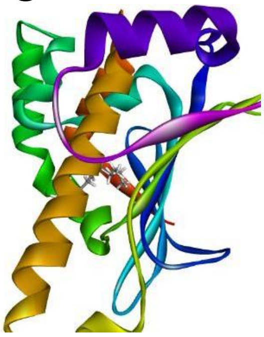

O`

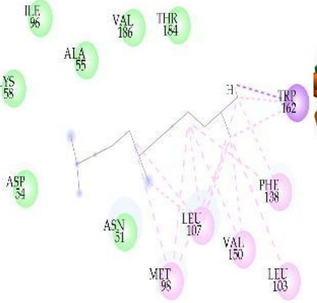

P

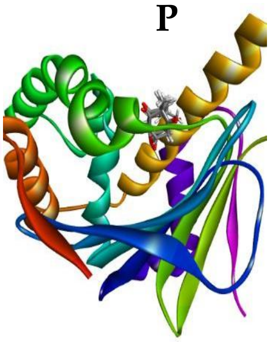

P`

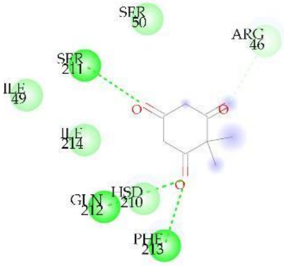

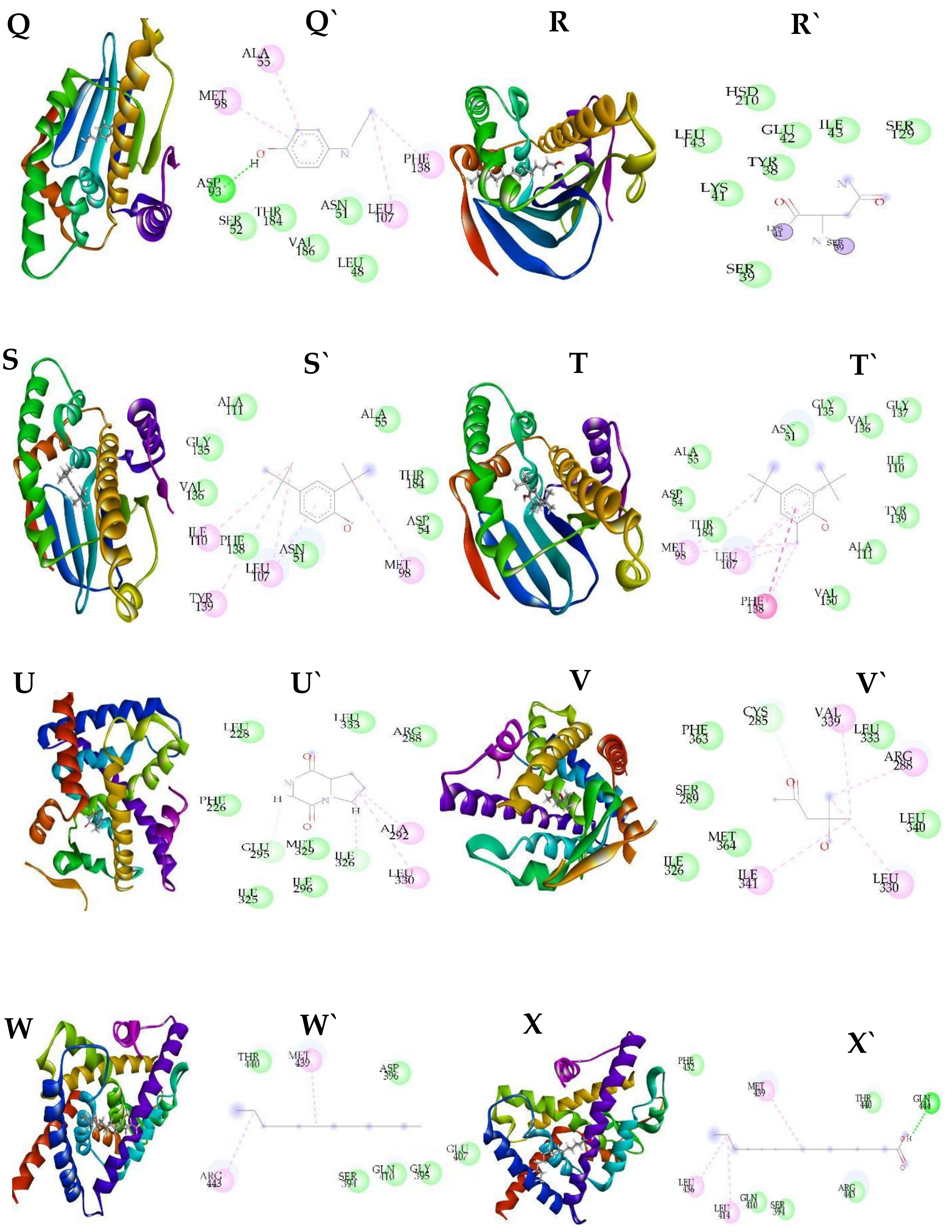
Q
Q`
ALA 55
MET 98
ASP 93
PHE 138
THR 184
ASN 51
LEU 107
SER 52
VAL 186
LEU 48
R
R`
HSD 210
LEU 143
GLU 42
ILE 43
SER 129
TYR 38
LYS 41
SER 39
S
S`
ALA 111
ALA 55
GLY 135
THR 184
VAL 136
ASP 54
ILE 110
PHE 138
ASN 51
LEU 107
MET 98
TYR 139
T
T`
GLY 135
GLY 137
VAL 136
ASN 51
ALA 55
ILE 110
ASP 54
TYR 139
THR 184
MET 98
LEU 107
ALA 111
PHE 138
VAL 150
U
U`
LEU 228
LEU 333
ARG 288
PHE 226
ALA 292
GLU 295
MET 329
ILE 326
LEU 330
ILE 296
ILE 325
V
V`
CYS 285
VAL 339
PHE 363
LEU 333
ARG 288
SER 289
LEU 340
MET 364
ILE 326
ILE 341
LEU 330
W
W`
THR 440
MET 439
ASP 396
ARG 443
SER 391
GLN 410
GLY 395
X
X`
PHE 432
GLU 407
MET 439
THR 440
GLN 444
LEU 436
ARG 443
GLN 410
SER 394
LEU 414

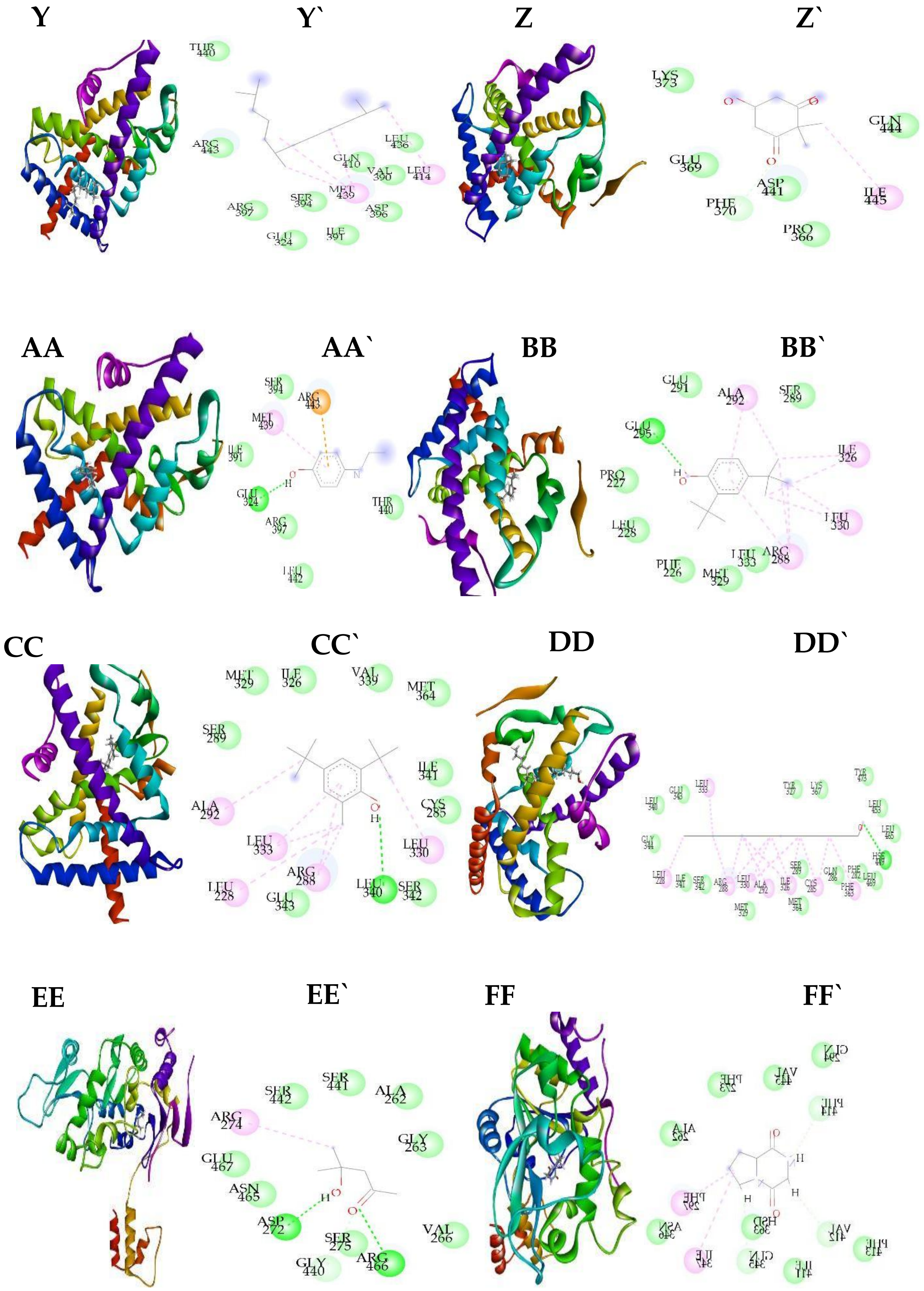
Y
Y`
Z
Z`
AA
AA`
BB
BB`
CC
CC`
DD
DD`
EE
EE`
FF
FF`

GG

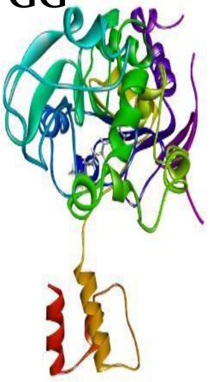

GG`

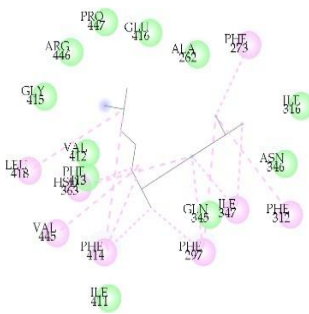

HH

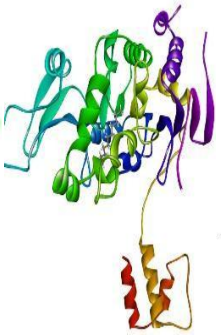

HH`

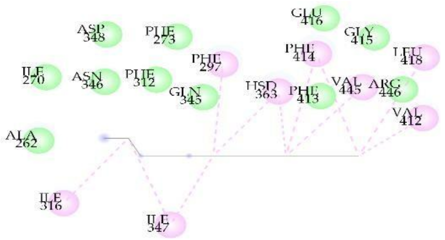

II`

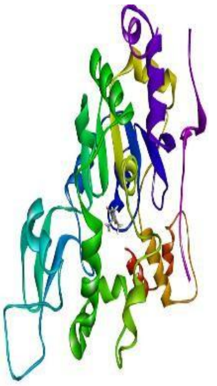

II`

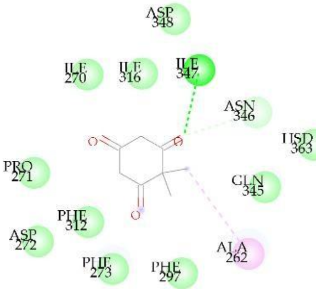

JJ

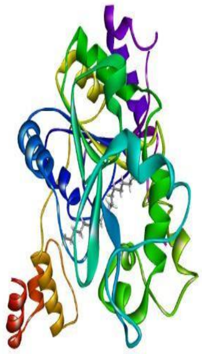

JJ`

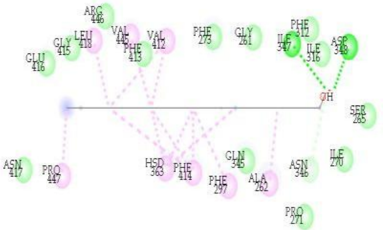

KK`

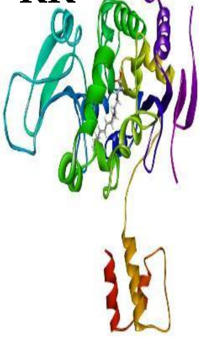

KK`

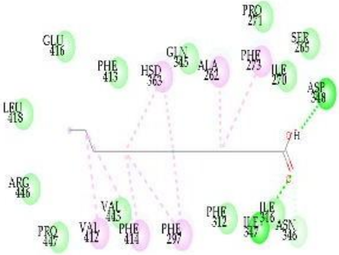

LL

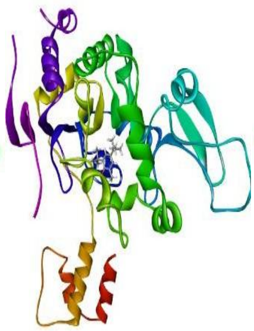

LL`

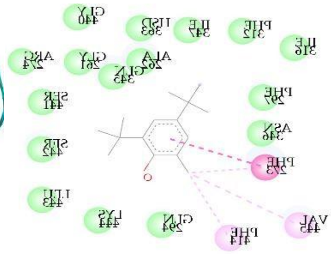

MM

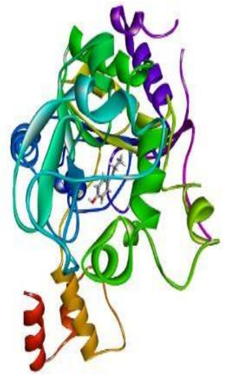

MM`

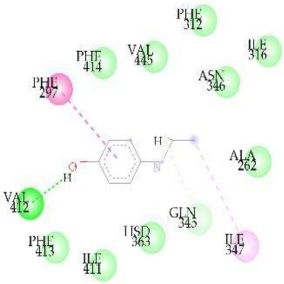

NN

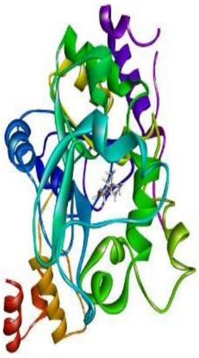

NN`

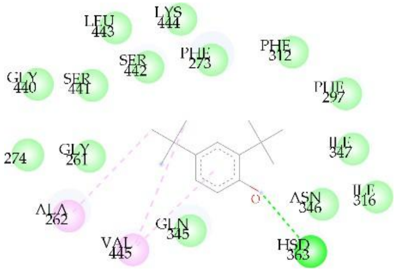

OO

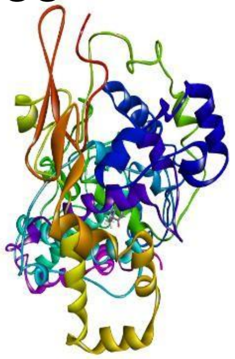

OO`

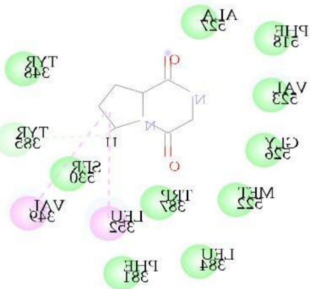

PP

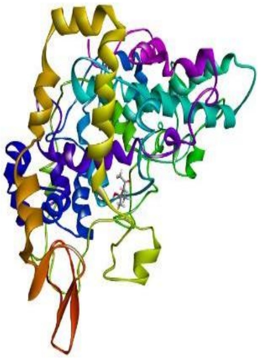

PP`

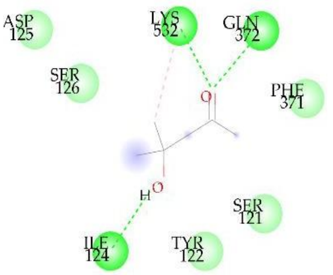

QQ

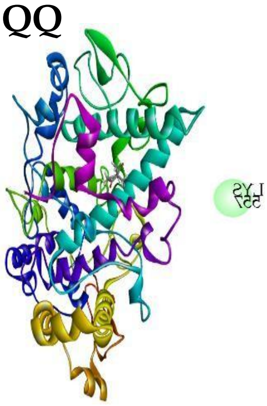

QQ`

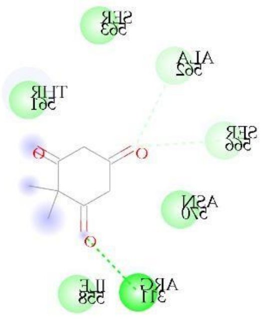

RR

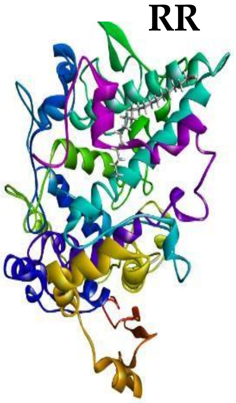

RR`

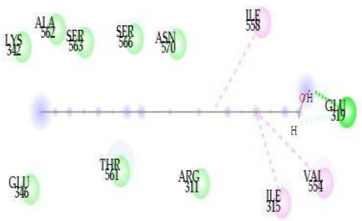

SS

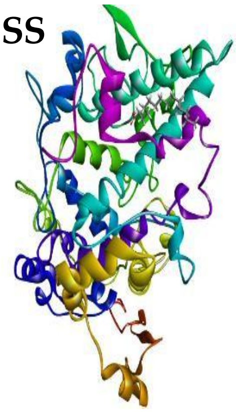

SS`

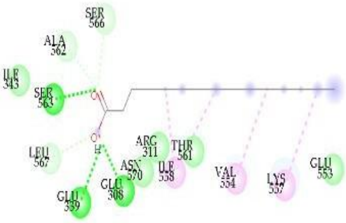

TT

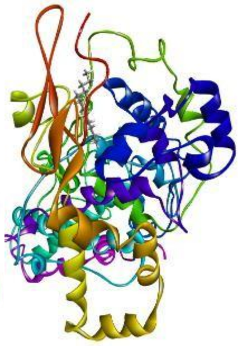

TT`

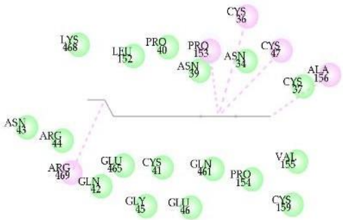

UU

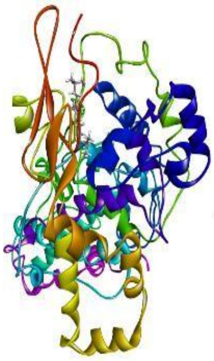

UU`

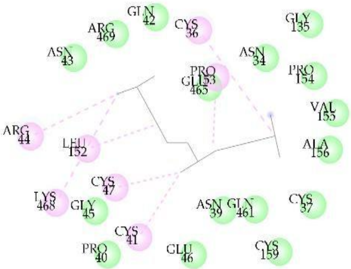

VV

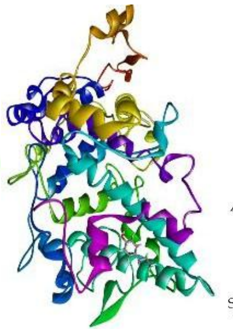

VV`

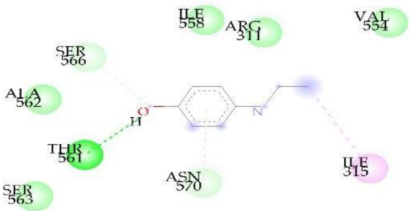

WW

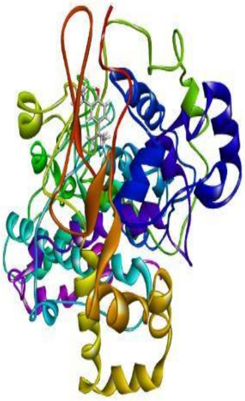

WW`

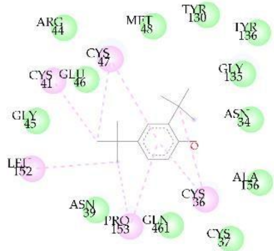

XX

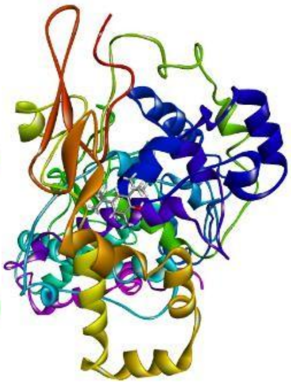

XX`

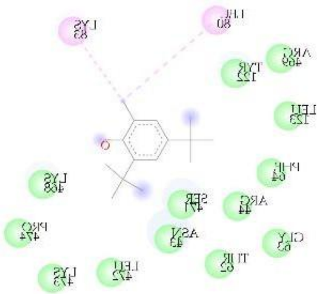

YY

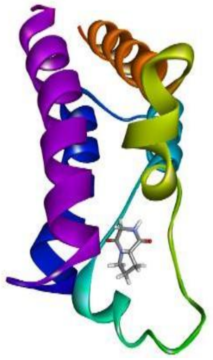

YY`

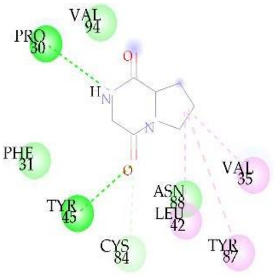

ZZ

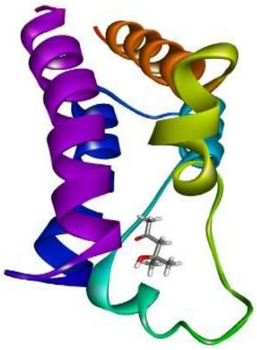

ZZ`

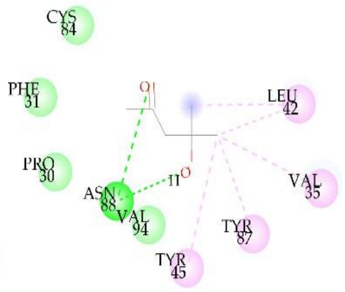

AAA

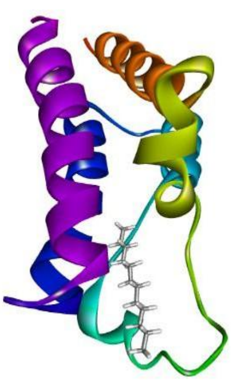

AAA`

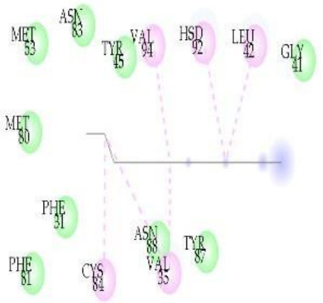

BBB

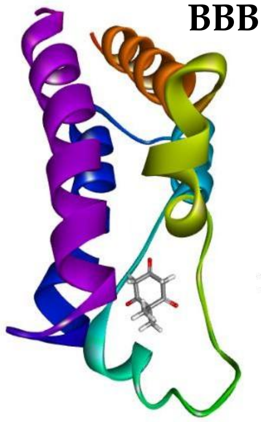

BBB`

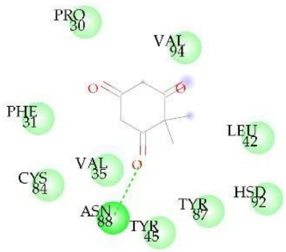

CCC

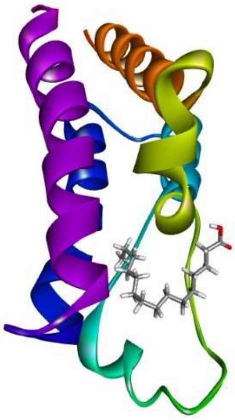

CCC`

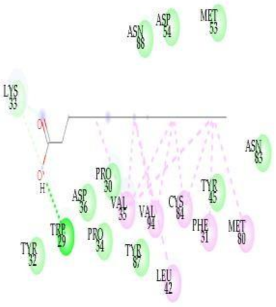

DDD

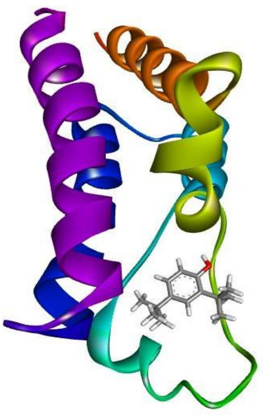

DDD`

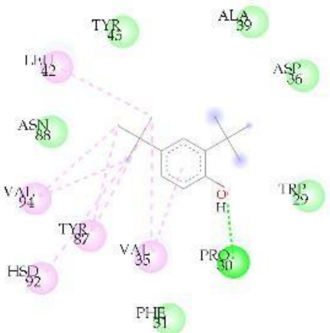

EEE

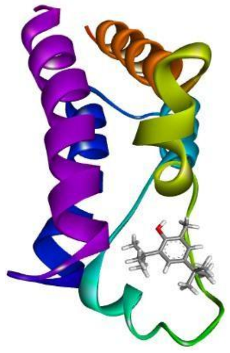

EEE`

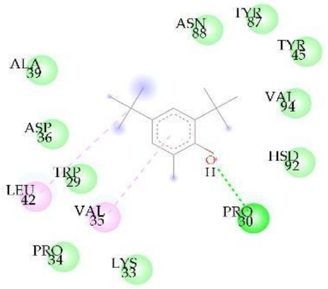

FFF

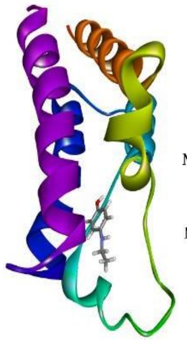

FFF`

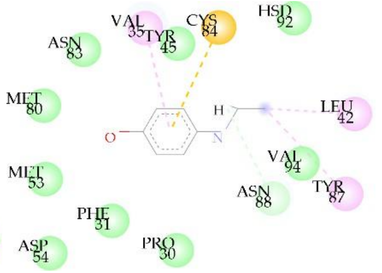

GGG

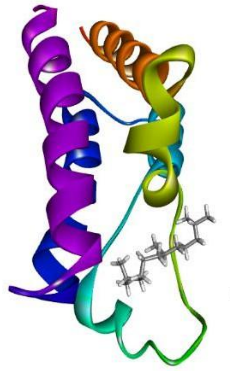

GGG`

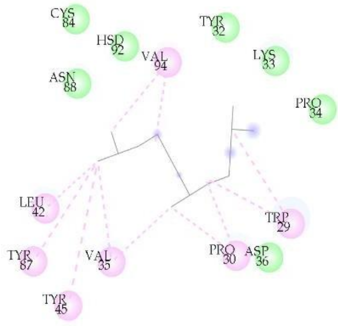

HHH

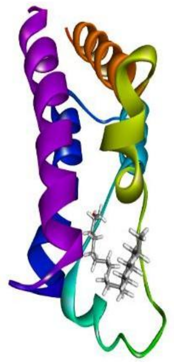

HHH`

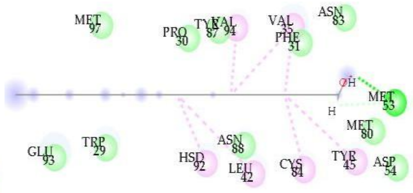

III

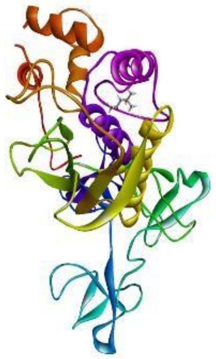

III`

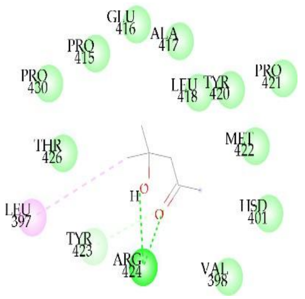

JJJ

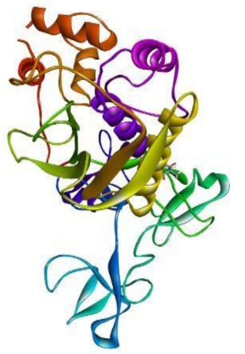

JJJ`

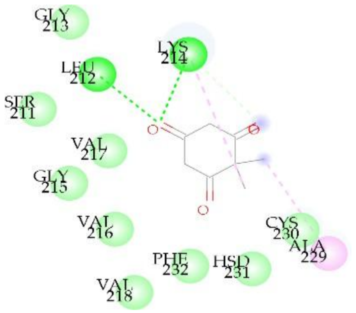

KKK

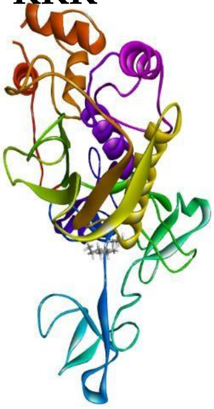

KKK`

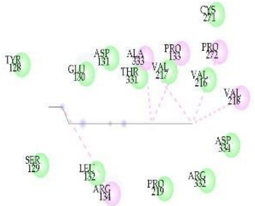

LLL

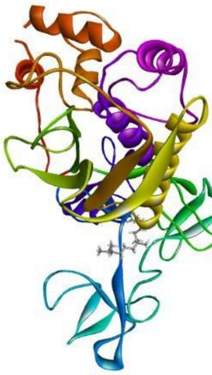

LLL`

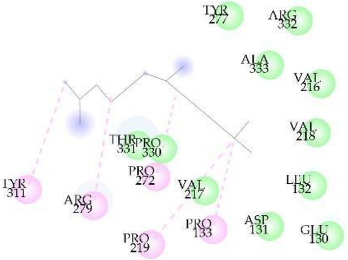

MMM

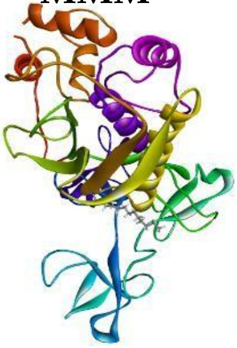

MMM`

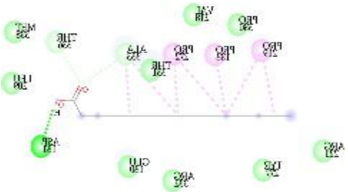

NNN

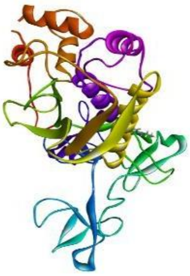

NNN`

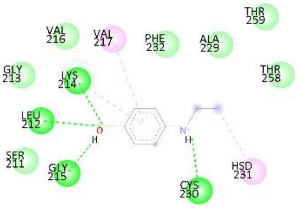

OOO

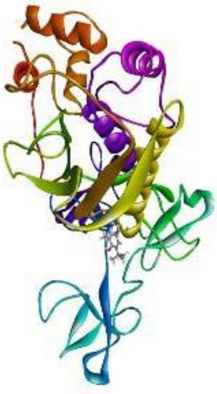

OOO`

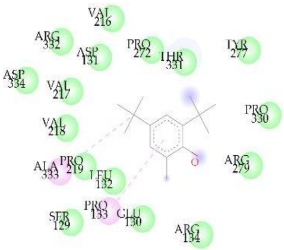

PPP

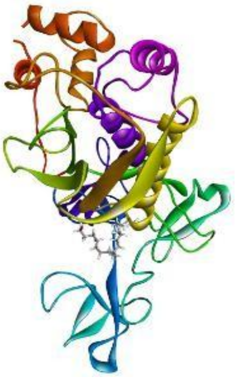

PPP`

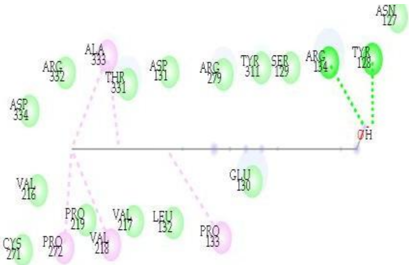

QQQ

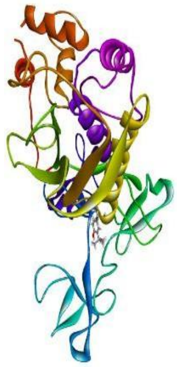

QQQ`

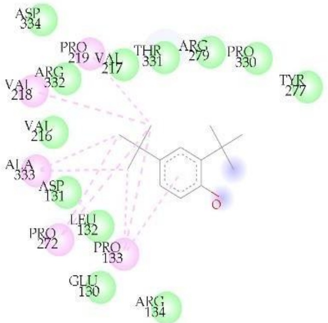

RRR

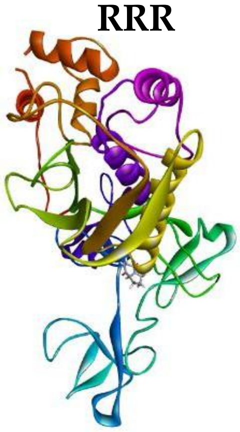

RRR`

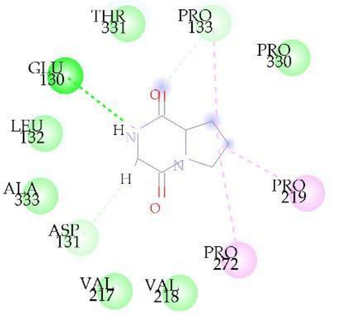

SSS

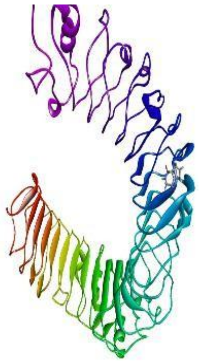

SSS`

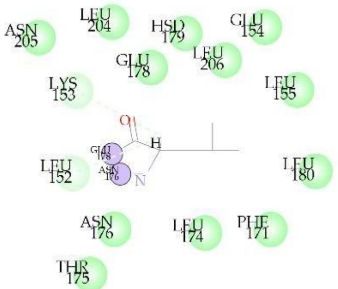

TTT

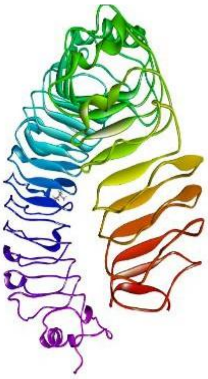

TTT`

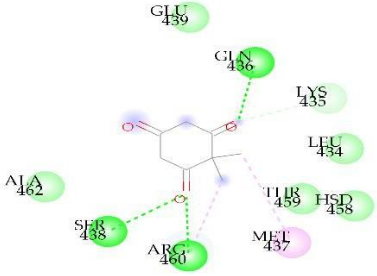

UUU

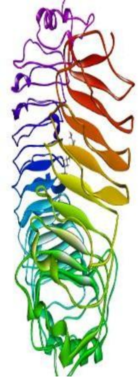

UUU`

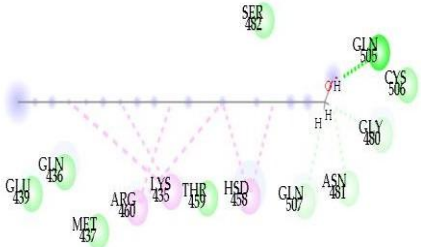

VVV

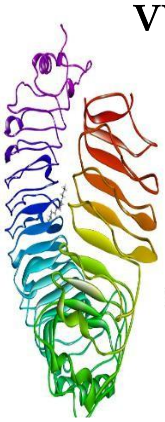

VVV`

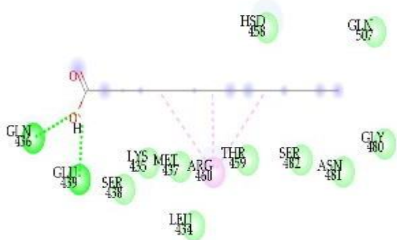

WWW

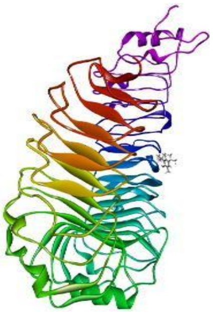

WWW`

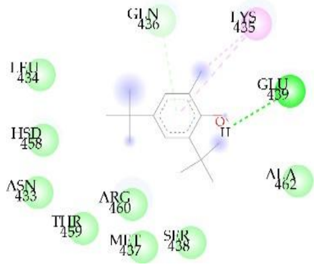

XXX

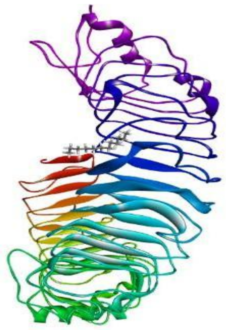

XXX`

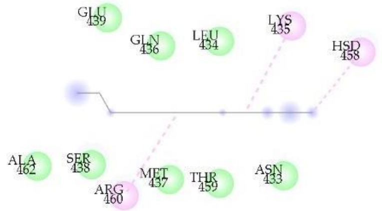

YYY

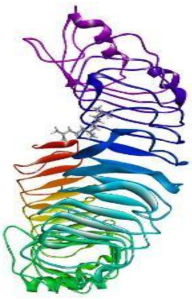

YYY`

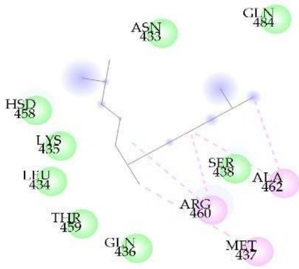

ZZZ

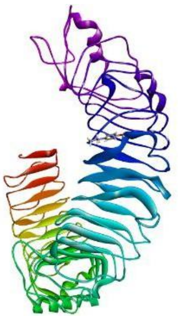

ZZZ`

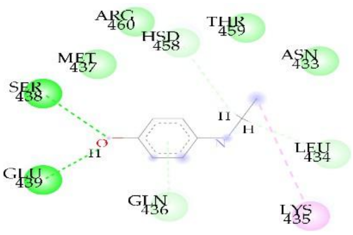

AAAA

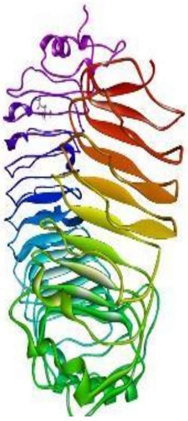

AAAA`

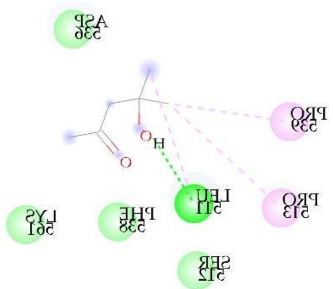

BBBB

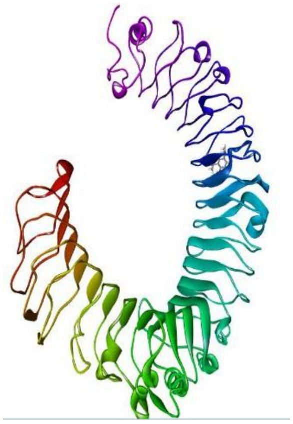

BBBB`

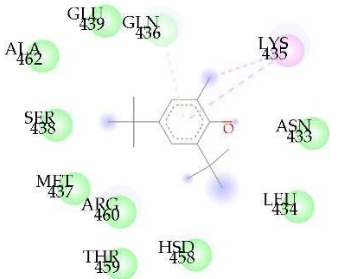

CCCC

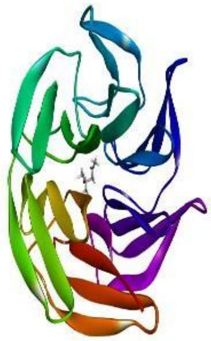

CCCC`

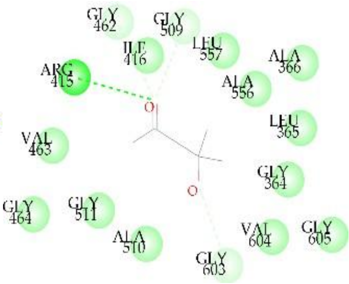

DDDD

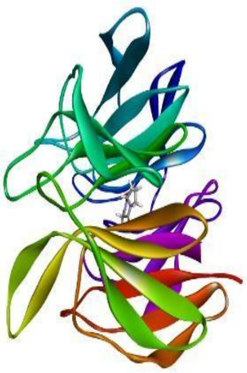

DDDD`

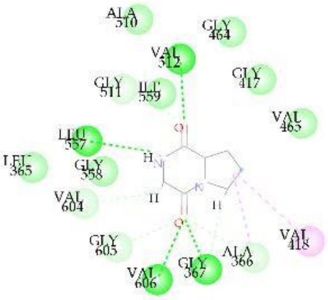

EEEE

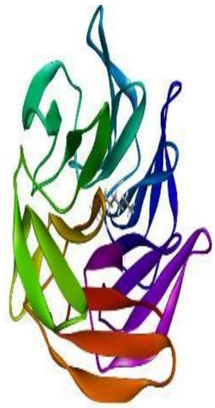

EEEE`

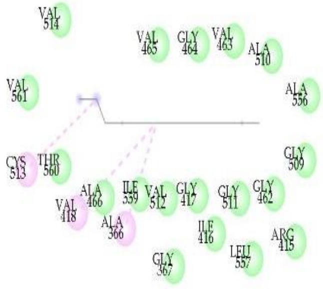

FFFF

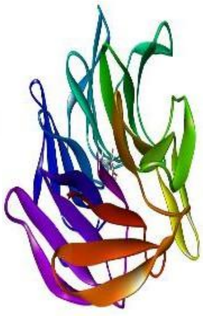

FFFF`

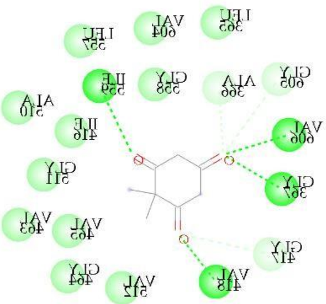

GGGG

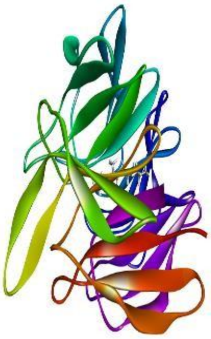

GGGG`

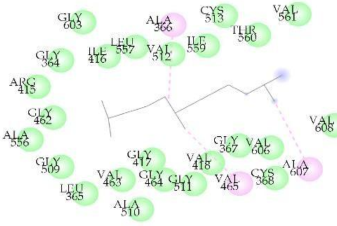

HHHH

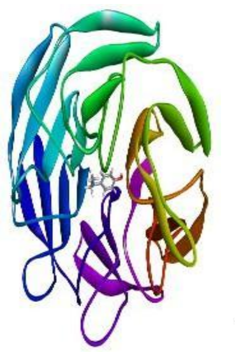

HHHH`

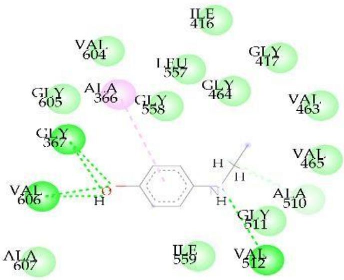

IIII

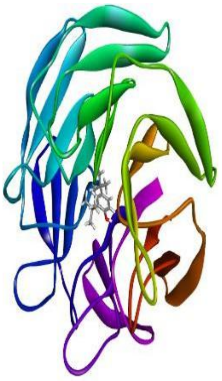

IIII`

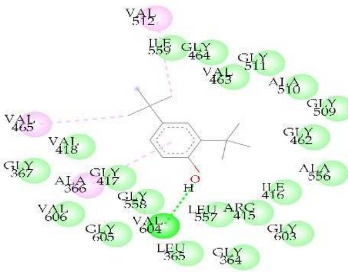

JJJJ

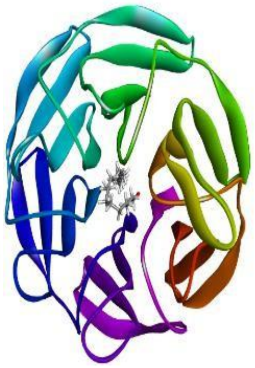

JJJJ`

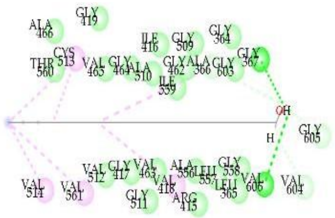

KKKK

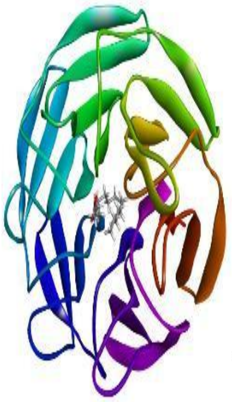

KKKK`

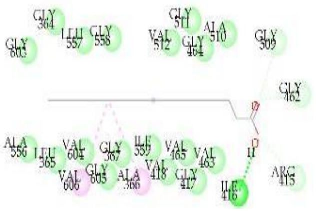

LLLL

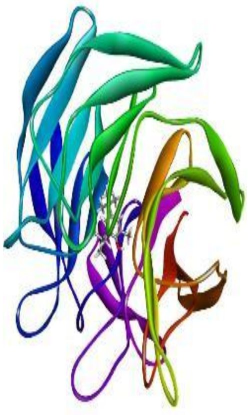

LLLL`

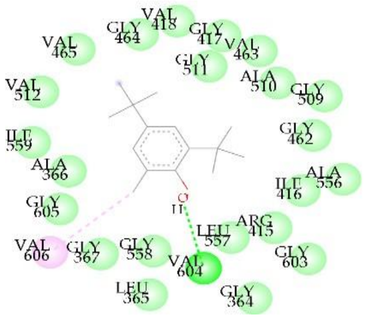

MMMM

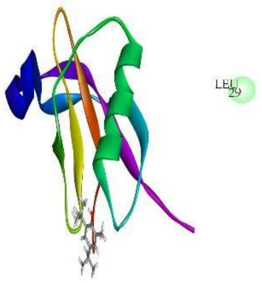

MMMM`

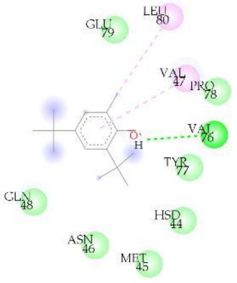

NNNN

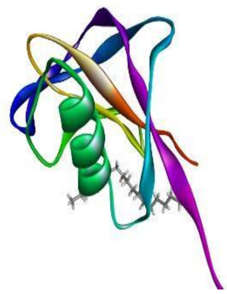

NNNN`

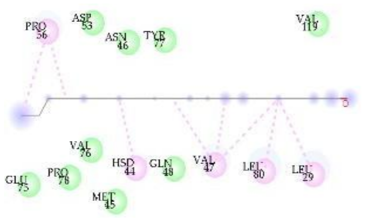

OOOO

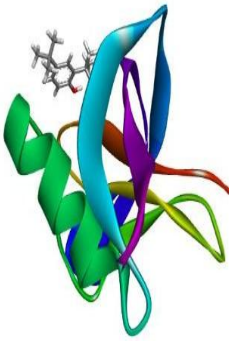

OOOO`

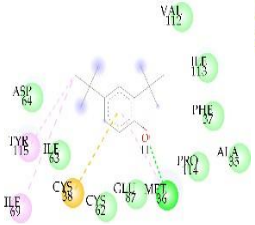

PPPP

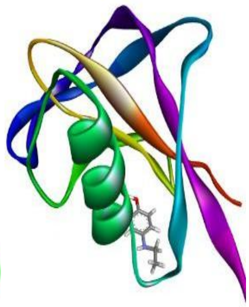

PPPP`

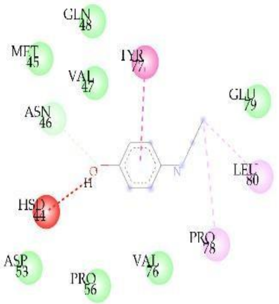

QQQQ

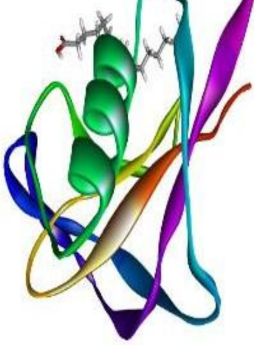

QQQQ`

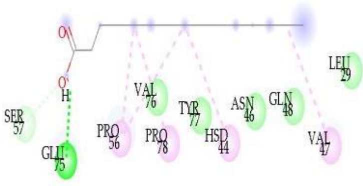

RRRR

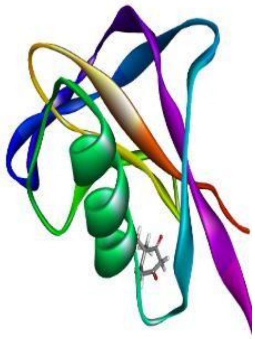

RRRR`

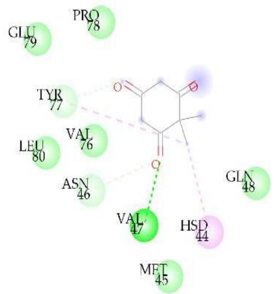

SSSS

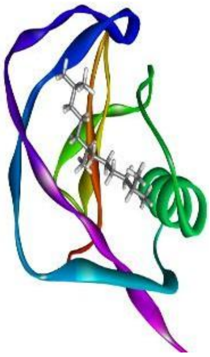

SSSS`

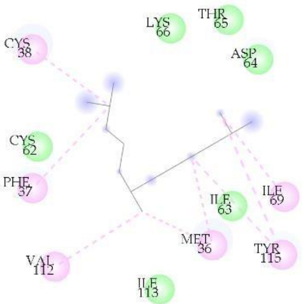

TTTT

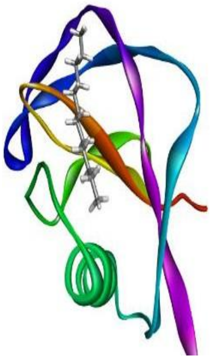

TTTT`

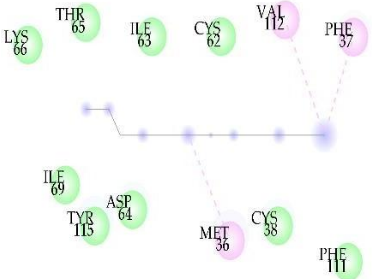

UUUU **UUUU`** **VVVV** **VVVV`**

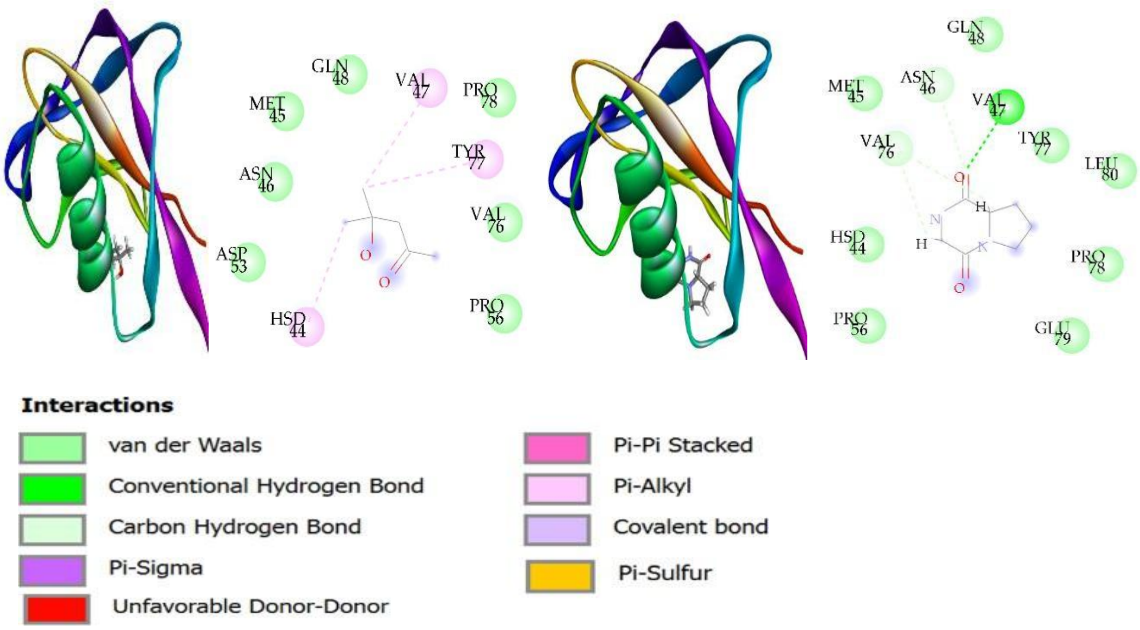

Figure S1. The formed interactions between the compounds and the key targets. (A) Filicilic acid–ERS1 (3D), (A`) filicilic acid–ERS1 (2D), (B) dodecane–ERS1 (3D), (B`) dodecane–ERS1 (2D), (C) 2-pentanone, 4-hydroxy-4-methyl–ERS1 (3D), (C) 2-pentanone, 4-hydroxy-4-methyl–ERS1 (2D), (D) pyrrolo[1,2-a]pyrazine-1,4-dione, hexahydro–ERS1 (3D), (D`) pyrrolo[1,2-a]pyrazine-1,4-dione, hexahydro–ERS1 (2D), (E) dodecane, 2,6,11-trimethyl–ERS1 (3D), (E`) dodecane, 2,6,11-trimethyl –ERS1 (2D), (F) n-nonadecanol–ERS1 (3D), (F`) n-nonadecanol–ERS1 (2D), (G) phenol, 4-(ethylamino)–ERS1 (3D), (G`) phenol, 4-(ethylamino)–ERS1 (2D), (H) pentadecanoic acid –ERS1 (3D), (H`) pentadecanoic acid–ERS1 (2D), (I) phenol, 4,6-di(1,1-dimethylethyl)-2-methyl–ERS1 (3D), (I`) phenol, 4,6-di(1,1-dimethylethyl)-2-methyl–ERS1 (2D), (J) phenol, 2,4-bis(1,1-dimethylethyl)–ERS1 (3D), (J`) phenol, 2,4-bis(1,1-dimethylethyl)–ERS1 (2D), (K) pyrrolo[1,2-a]pyrazine-1,4-dione, hexahydro–HSP90AA1 (3D), (K`) pyrrolo[1,2-a]pyrazine-1,4-dione, hexahydro–HSP90AA1 (2D), (L) pentadecanoic acid–HSP90AA1 (3D), (L`) pentadecanoic acid–HSP90AA1 (2D), (M) dodecane–HSP90AA1 (3D), (M`) dodecane–HSP90AA1 (2D), (N) n-nonadecanol–HSP90AA1 (3D), (N`) n-nonadecanol–HSP90AA1 (2D), (O) dodecane, 2,6,11-trimethyl–HSP90AA1 (3D), (O`) dodecane, 2,6,11-trimethyl–HSP90AA1 (2D), (P) filicilic acid–HSP90AA1 (3D), (P`) filicilic acid–HSP90AA1 (2D), (Q) phenol, 4-(ethylamino)–HSP90AA1 (3D), (Q`) phenol, 4-(ethylamino)–HSP90AA1 (2D), (R) pentadecanoic acid–HSP90AA1 (3D), (R`) pentadecanoic acid–HSP90AA1 (2D), (S) phenol, 2,4-bis(1,1-dimethylethyl)–HSP90AA1 (3D), (S`) phenol, 2,4-bis(1,1-dimethylethyl)–HSP90AA1 (2D), (T) phenol, 4,6-di(1,1-dimethylethyl)-2-methyl–HSP90AA1 (3D), (T`) phenol, 4,6-di(1,1-dimethylethyl)-2-methyl –HSP90AA1 (2D), (U) pyrrolo[1,2-a]pyrazine-1,4-dione, hexahydro–PPARG (3D), (U`) pyrrolo[1,2-a]pyrazine-1,4-dione, hexahydro–PPARG (2D), (V) 2-pentanone, 4-hydroxy-4-methyl–PPARG (3D), (V`) 2-pentanone, 4-hydroxy-4-methyl–PPARG (2D), (W) dodecane–PPARG (3D), (W`) dodecane–PPARG (2D), (X) pentadecanoic acid–PPARG (3D), (X`) pentadecanoic acid–PPARG (2D), (Y) dodecane, 2,6,11-trimethyl–PPARG (3D), (Y`) dodecane, 2,6,11-trimethyl–PPARG (2D), (Z) filicilic acid–PPARG (3D), (Z`) filicilic acid–PPARG (2D), (AA) phenol, 4-(ethylamino)–PPARG (3D), (AA`)

phenol, 4-(ethylamino)–PPARG (2D), (BB) phenol, 2,4-bis(1,1-dimethylethyl)–PPARG (3D), (BB`) phenol, 2,4-bis(1,1-dimethylethyl)–PPARG (2D), (CC) phenol, 4,6-di(1,1-dimethylethyl)-2-methyl –PPARG (3D), (CC`) phenol, 4,6-di(1,1-dimethylethyl)-2-methyl–PPARG (2D), (DD) n-Nonadecanol-1–PPARG (3D), (DD`) n-Nonadecanol-1–PPARG (2D), (EE) 2-pentanone, 4-hydroxy-4-methyl–SIRT1 (3D), (EE`) 2-pentanone, 4-hydroxy-4-methyl–SIRT1 (2D), (FF) pyrrolo[1,2-a]pyrazine-1,4-dione, hexahydro–SIRT1 (3D), (FF`) pyrrolo[1,2-a]pyrazine-1,4-dione, hexahydro–SIRT1 (2D), (GG) dodecane, 2,6,11-trimethyl–SIRT1 (3D), (GG`) dodecane, 2,6,11-trimethyl–SIRT1 (2D), (HH) dodecane–SIRT1 (3D), (HH`) dodecane–SIRT1 (2D), (II) filicinic acid–SIRT1 (3D), (II`) filicinic acid–SIRT1 (2D), (JJ) n-nonadecanol-1–SIRT1 (3D), (JJ`) n-nonadecanol-1–SIRT1 (2D), (KK) pentadecanoic acid–SIRT1 (3D), (KK`), pentadecanoic acid–SIRT1 (2D), (LL) phenol, 4,6-di(1,1-dimethylethyl)-2-methyl–SIRT1 (3D), (LL`) phenol, 4,6-di(1,1-dimethylethyl)-2-methyl–SIRT1 (2D), (MM) phenol, 4-(ethylamino)–SIRT1 (3D), (MM`) phenol, 4-(ethylamino)–SIRT1 (2D), (NN) phenol, 2,4-bis(1,1-dimethylethyl)–SIRT1 (3D), (NN`) phenol, 2,4-bis(1,1-dimethylethyl)–SIRT1 (2D), (OO), pyrrolo[1,2-a]pyrazine-1,4-dione, hexahydro–PTGS2 (3D), (OO`) pyrrolo[1,2-a]pyrazine-1,4-dione, hexahydro–PTGS2 (2D), (PP) 2-pentanone, 4-hydroxy-4-methyl–PTGS2 (3D), (PP`) 2-pentanone, 4-hydroxy-4-methyl–PTGS2 (2D), (QQ) filicinic acid–PTGS2 (3D), (QQ`) filicinic acid–PTGS2 (2D), (RR) n-nonadecanol-1–PTGS2 (3D), (RR`) n-nonadecanol-1–PTGS2 (2D), (SS) pentadecanoic acid–PTGS2 (3D), (SS`) pentadecanoic acid–PTGS2 (2D), (TT) dodecane –PTGS2 (3D), (TT`) Dodecane –PTGS2 (2D), (UU) Dodecane, 2,6,11-trimethyl – PTGS2 (3D), (UU`), dodecane, 2,6,11-trimethyl–PTGS2 (2D), (VV) phenol, 4-(ethylamino)–PTGS2 (3D), (VV`) phenol, 4-(ethylamino)–PTGS2 (2D), (WW) phenol, 2,4-bis(1,1-dimethylethyl)-–PTGS2 (3D), (WW`) phenol, 2,4-bis(1,1-dimethylethyl)–PTGS2 (2D), (XX) phenol, 4,6-di(1,1-dimethylethyl)-2-methyl, (XX`) phenol, 4,6-di(1,1-dimethylethyl)-2-methyl–PTGS2 (2D), (YY) pyrrolo[1,2-a]pyrazine-1,4-dione, hexahydro–RELA (3D), (YY`) pyrrolo[1,2-a]pyrazine-1,4-dione, hexahydro–RELA (2D), (ZZ) 2-pentanone, 4-hydroxy-4-methyl–RELA (3D), (ZZ`) 2-pentanone, 4-hydroxy-4-methyl–RELA (2D), (AAA) dodecane–RELA (3D), (AAA`) dodecane–RELA (2D), (BBB) filicinic acid–RELA (3D), (BBB`) filicinic acid–RELA (2D), (CCC) pentadecanoic acid–RELA (3D), (CCC`) pentadecanoic acid–RELA (2D), (DDD) phenol, 2,4-bis(1,1-dimethylethyl)–RELA (3D), (DDD`) phenol, 2,4-bis(1,1-dimethylethyl)-1–RELA (2D), (EEE) phenol, 4,6-di(1,1-dimethylethyl)-2-methyl–RELA (3D), (EEE`), phenol, 4,6-di(1,1-dimethylethyl)-2-methyl–RELA (2D), (FFF) phenol, 4-(ethylamino)–RELA (3D), (FFF`) phenol, 4-(ethylamino)–RELA (2D), (GGG) dodecane, 2,6,11-trimethyl–RELA (3D), (GGG`) dodecane, 2,6,11-trimethyl–RELA (2D), (HHH) n-nonadecanol-1–RELA (3D), (HHH`) n-nonadecanol-1–RELA (2D), (III) 2-pentanone, 4-hydroxy-4-methyl–MMP9 (3D), (III`) 2-pentanone, 4-hydroxy-4-methyl–MMP9 (2D), (JJJ) filicinic acid–MMP9 (3D), (JJJ`) filicinic acid–MMP9 (2D), (KKK) dodecane–MMP9 (3D), (KKK`) dodecane–MMP9 (2D), (LLL) dodecane, 2,6,11-trimethyl–MMP9 (3D), (LLL`) dodecane, 2,6,11-trimethyl–MMP9 (2D), (MMM) pentadecanoic acid–MMP9 (3D), (MMM`) pentadecanoic acid– MMP9 (2D), (NNN) phenol, 4-(ethylamino)– MMP9 (3D), (NNN`) phenol, 4-(ethylamino)–MMP9 (2D), (OOO) phenol, 4,6-di(1,1-dimethylethyl)-2-methyl–MMP9 (3D), (OOO`), phenol, 4,6-di(1,1-dimethylethyl)-2-methyl–MMP9 (2D), (PPP) n-nonadecanol-1–MMP9 (3D), (PPP`) n-nonadecanol-1–MMP9 (2D), (QQQ) phenol, 2,4-bis(1,1-dimethylethyl)–MMP9 (3D), (QQQ`) phenol, 2,4-bis(1,1-dimethylethyl)–MMP9 (2D), (RRR), pyrrolo[1,2-a]pyrazine-1,4-dione, hexahydro–MMP9 (3D), (RRR`) pyrrolo[1,2-a]pyrazine-1,4-dione, hexahydro–MMP9 (2D), (SSS) pyrrolo[1,2-a]pyrazine-1,4-dione, hexahydro–TLR4 (3D), (SSS`) pyrrolo[1,2-a]pyrazine-1,4-dione, hexahydro–TLR4 (2D), (TTT) filicinic acid–TLR4 (3D), (TTT`) filicinic acid–TLR4 (2D), (UUU) pentadecanoic acid–TLR4 (3D), (UUU`) pentadecanoic acid–TLR4 (2D), (VVV) n-nonadecanol-1–TLR4 (3D), (VVV`) n-nonadecanol-1–TLR4 (2D), (WWW) phenol, 4,6-di(1,1-dimethylethyl)-2-methyl –TLR4 (3D), (WWW`) phenol, 4,6-di(1,1-dimethylethyl)-2-methyl–TLR4 (2D), (XXX) dodecane–TLR4 (3D), (XXX`) dodecane–TLR4 (2D), (YYY) dodecane, 2,6,11-trimethyl–TLR4 (3D), (YYY`), dodecane, 2,6,11-trimethyl–TLR4 (2D), (ZZZ) phenol, 4-(ethylamino)–TLR4 (3D), (ZZZ`) phenol, 4-(ethylamino)–TLR4

(2D), (AAAA) 2-pentanone, 4-hydroxy-4-methyl–TLR4 (3D), (AAAA`) 2-pentanone, 4-hydroxy-4-methyl–TLR4 (2D), (BBBB) phenol, 4,6-di(1,1-dimethylethyl)-2-methyl, (BBBB`) phenol, 4,6-di(1,1-dimethylethyl)-2-methyl–TLR4 (2D), (CCCC) 2-pentanone, 4-hydroxy-4-methyl–NFE2L2 (3D), (CCCC`) 2-pentanone, 4-hydroxy-4-methyl–NFE2L2 (2D), (DDDD) pyrrolo[1,2-a]pyrazine-1,4-dione, hexahydro–NFE2L2 (3D), (DDDD`) pyrrolo[1,2-a]pyrazine-1,4-dione, hexahydro–NFE2L2 (2D), (EEEE) dodecane–NFE2L2 (3D), (EEEE`) dodecane–TLR4 (2D), (FFFF) filicinic acid–NFE2L2 (3D), (FFFF`) filicinic acid–NFE2L2 (2D), (GGGG) dodecane, 2,6,11-trimethyl–NFE2L2 (3D), (GGGG`) dodecane, 2,6,11-trimethyl–NFE2L2 (2D), (HHHH) phenol, 4-(ethylamino)–NFE2L2 (3D), (HHHH`) phenol, 4-(ethylamino)–NFE2L2 (2D), (IIII) phenol, 2,4-bis(1,1-dimethylethyl)–NFE2L2 (3D), (IIII`), phenol, 2,4-bis(1,1-dimethylethyl)–NFE2L2 (2D), (JJJJ) n-nonadecanol-1–NFE2L2 (3D), (JJJJ`) n-nonadecanol-1–NFE2L2 (2D), (KKKK) pentadecanoic acid–NFE2L2 (3D), (KKKK`) pentadecanoic acid–NFE2L2 (2D), (LLLL) phenol, 4,6-di(1,1-dimethylethyl)-2-methyl, (LLLL`) phenol, 4,6-di(1,1-dimethylethyl)-2-methyl–NFE2L2 (2D), (MMMM) phenol, 4,6-di(1,1-dimethylethyl)-2-methyl–APP (3D), (MMMM`) phenol, 4,6-di(1,1-dimethylethyl)-2-methyl–APP (2D), (NNNN) n-nonadecanol-1–APP (3D), (NNNN`) n-nonadecanol-1– APP (2D), (OOOO) phenol, 2,4-bis(1,1-dimethylethyl)–APP (3D), (OOOO`) phenol, 2,4-bis(1,1-dimethylethyl)–APP (2D), (PPPP) phenol, 4-(ethylamino)–APP (3D), (PPPP`) phenol, 4-(ethylamino)–APP (2D), (QQQQ) pentadecanoic acid–APP (3D), (QQQQ`) pentadecanoic acid–APP (2D), (VVVV) filicinic acid–APP (3D), (VVVV`) filicinic acid–APP (2D), (WWWW) dodecane, 2,6,11-trimethyl–APP (3D), (WWWW`), dodecane, 2,6,11-trimethyl–APP (2D), (XXXX) dodecane–APP (3D), (XXXX`) dodecane–APP (2D), (YYYY) 2-pentanone, 4-hydroxy-4-methyl–APP (3D), (YYYY`) 2-pentanone, 4-hydroxy-4-methyl–APP (2D), (ZZZZ) pyrrolo[1,2-a]pyrazine-1,4-dione, hexahydro–APP (ZZZZ`) pyrrolo[1,2-a]pyrazine-1,4-dione, hexahydro–APP (2D).
